# Trigonal Planar Heteroleptic Lanthanide(III) Bis(silyl)amide Complexes Containing Aminoxyl Radicals and Anions

**DOI:** 10.1021/acs.inorgchem.4c03281

**Published:** 2024-11-12

**Authors:** Gemma K. Gransbury, Hannah M. Nicholas, Siobhan R. Murphy, Jack Emerson-King, Michele Vonci, Conrad A. P. Goodwin, Richard E. P. Winpenny, Nicholas F. Chilton, Marcus J. Giansiracusa, David P. Mills

**Affiliations:** † Department of Chemistry, The University of Manchester, Oxford Road, Manchester M13 9PL, U.K.; ‡ Research School of Chemistry, Australian National University, Building 137, Sullivans Creek Road, Canberra, ACT 2601, Australia; § School of Chemistry, The University of Melbourne, Parkville, Victoria 3010, Australia

## Abstract

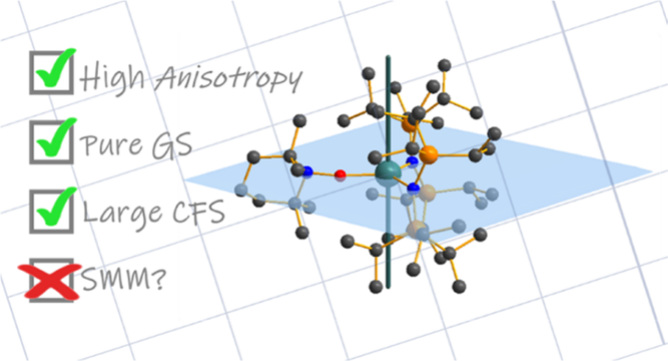

Modulation of the crystal field (CF) in lanthanide (Ln) complexes can enhance optical and magnetic properties, and large CF splitting can be achieved with low coordination numbers in specific geometries. We previously reported that the homoleptic near-linear Sm^2+^ complex [Sm^II^{N(Si^i^Pr_3_)_2_}_2_] (**1-Sm**) is oxidized by the 2,2,6,6-tetramethylpiperidinyl-1-oxy (TEMPO^•^) radical to give the heteroleptic, approximately trigonal planar Sm^3+^ complex, [Sm^III^{N(Si^i^Pr_3_)_2_}_2_(TEMPO^–^)] (**2-Sm**). Here, we report the synthesis of homologous [Ln^III^{N(Si^i^Pr_3_)_2_}_2_(TEMPO^–^)] (**2-Ln**; Ln = Tm, Yb) complexes by the oxidation of the parent [Ln{N(Si^i^Pr_3_)_2_}_2_] (**1-Ln**; Ln = Tm, Yb) with TEMPO^•^; complexes **2-Ln** all contain TEMPO^–^ anions. The homoleptic bent Ln^3+^ complexes [Ln^III^{N(Si^i^Pr_3_)_2_}_2_][B(C_6_F_5_)_4_] (**3-Ln**; Ln = Sm, Tm, Yb) were also treated with TEMPO^•^ to yield the heteroleptic, approximately trigonal planar Ln^3+^ complexes [Ln^III^{N(Si^i^Pr_3_)_2_}_2_(TEMPO^•^)][B(C_6_F_5_)_4_] (**4-Ln**; Ln = Sm, Tm, Yb); the cations of **4-Ln** all contain TEMPO^•^ radicals. We have compared the electronic structures of the two geometrically similar families of Ln^3+^ complexes with the TEMPO^–^ anion (**2-Ln**) or TEMPO^•^ radical (**4-Ln**) using a combination of UV–vis-NIR and EPR spectroscopy, magnetic measurements, and *ab initio* calculations. These studies revealed no single-molecule magnet behavior for **2-Yb** despite evidence for sizable CF splitting and a high degree of purity of the ground stabilized *m*_J_ = |±7/2⟩ state, while the radical TEMPO^•^ in **4-Yb** did not significantly improve performance.

## Abstract 2

We report the synthesis and characterization of a series of lanthanide(III) bis-amide aminoxyl complexes, where the aminoxyls are either anionic or neutral radical ligands. We find high anisotropy, large crystal field splitting, and pure ground states in Yb^3+^ examples, but no single-molecule magnet behavior.

## Introduction

Lanthanide (Ln) compounds often exhibit fascinating properties,^1^ and low-coordinate Ln complexes with precise geometries can display enhanced optical^2^ and magnetic behavior^3−5^ due to the influence of the crystal field (CF) on electronic structures. Coordinatively unsaturated Ln complexes are often challenging to synthesize as large Ln cations exhibit predominantly electrostatic bonding with ligands, promoting high coordination numbers.^1^ Sterically demanding ligands can be used to saturate Ln coordination spheres; however, heteroleptic low-coordinate Ln complexes are still relatively scarce due to facile Schlenk-type equilibria, therefore advances in ligand design are required to prepare larger families of these complexes to define their electronic structures.^6,7^ Bis(silyl)amides have been proven to be particularly adept at stabilizing low-coordinate Ln complexes as they combine relatively hard N-donor atoms with numerous additional Ln···Cγ–Siβ electrostatic interactions.^8^

The near-linear Ln^2+^ complexes [Ln^II^{N(Si^i^Pr_3_)_2_}_2_] (**1-Ln**, Ln = Sm, Eu, Tm, Yb) were synthesized by salt metathesis methods;^9,10^ the single electron transfer (SET) reaction of **1-Sm** with 2,2,6,6-tetramethylpiperidinyl-1-oxy radical (TEMPO^•^) gave the approximately trigonal planar Sm^3+^ complex [Sm^III^{N(Si^i^Pr_3_)_2_}_2_(TEMPO^–^)] (**2-Sm**), where the resultant TEMPO^–^ anion exhibits a κ^1^-*O* binding mode.^10^ Lanthanide radical compounds and their magnetic properties have been studied for many years,^11−14^ including as single-molecule magnets (SMMs),^15,16^ and an early family of isostructural single chain magnets, where the anisotropy and exchange coupling are tuned by the identity of Ln.^17,18^ However, there are only a handful of structurally characterized TEMPO^•^ radicals coordinated to Ln^3+^ centers.^19−25^ TEMPO^•^ has been most commonly employed in low oxidation state f-block chemistry as an oxidant to deliver O^2–^^26−31^ or TEMPO^–^ anions.^10,32−36^ Of most relevance here, in 2012 Evans and co-workers reported that the oxidation of [{Y^III^[N(SiMe_3_)_2_]_2_(THF)}_2_(μ,η^2^:η^2^-N_2_)]_2_ with TEMPO^•^ yielded the Y^3+^ bis(silyl)amide complex [Y^III^{N(SiMe_3_)_2_}_2_(η^2^-TEMPO^–^)(THF)], which exhibits a TEMPO^–^ anion binding side-on *via* both oxygen and nitrogen atoms.^33^ More recently, some of us disclosed the syntheses of the bent Ln^3+^ complexes [Ln^III^{N(Si^i^Pr_3_)_2_}_2_][B(C_6_F_5_)_4_] (**3-Ln**; Ln = Sm, Dy, Tm, Yb).^37,38^ We envisaged that **3-Ln** would be bound more readily by Lewis bases than **1-Ln** due to a combination of more accessible reactive sites provided by their bent geometries and more Lewis acidic Ln^3+^ centers.^1^

Here, we report the synthesis of approximately trigonal planar Ln^3+^ complexes, [Ln^III^{N(Si^i^Pr_3_)_2_}_2_(TEMPO^–^)] (**2-Ln**, Ln = Tm, Yb), by the SET reactions of TEMPO^•^ with the parent near-linear Ln^2+^ complexes **1-Ln**; analogously to **2-Sm**, the Tm and Yb analogues contain TEMPO^–^ anions. The separate reactions of bent Ln^3+^ complexes **3-Ln** with TEMPO^•^ provided the approximately trigonal planar Ln^3+^ complexes [Ln^III^{N(Si^i^Pr_3_)_2_}_2_(TEMPO^•^)][B(C_6_F_5_)_4_] (**4-Ln**; Ln = Sm, Tm, Yb), which contain TEMPO^•^ radicals. The electronic structures of structurally similar **2-Ln** and **4-Ln** cations were analyzed by UV–vis-NIR and EPR spectroscopy, magnetic measurements, and *ab initio* calculations. We find evidence of large CF splitting in Yb and Tm complexes but no slow relaxation behavior for **2-Yb** despite the highly pure ground state. Exchange interactions with TEMPO^•^ radicals are estimated as no more than tens of wavenumbers, with **4-Yb** having exchange states split by ∼0.32 cm^–1^ and spindle-shaped hysteresis.

## Results

### Synthesis

**Scheme 1:**
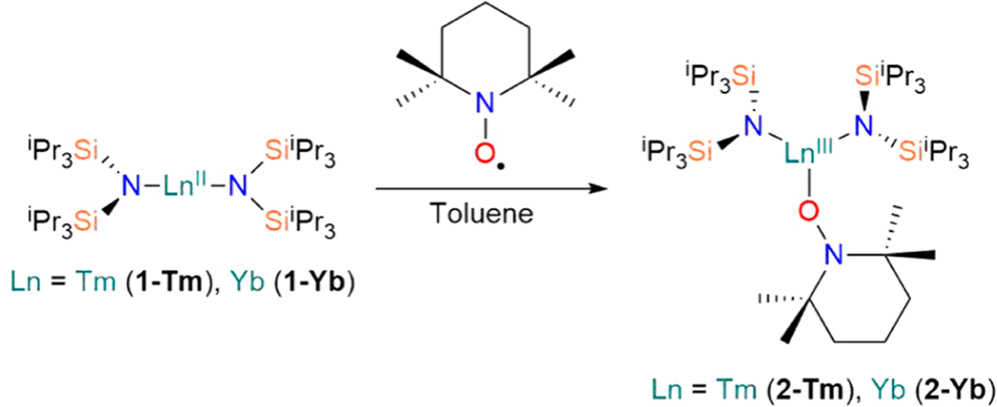
Synthesis of [Ln^III^{N(Si^i^Pr_3_)_2_}_2_(TEMPO^–^)] (**2-Ln**; Ln = Tm, Yb); the Synthesis of **2-Sm** Has Previously Been Reported in Ref (10)

Analogously to the synthesis of [Sm^III^{N(Si^i^Pr_3_)_2_}_2_(TEMPO^–^)],^10^ the heteroleptic three-coordinate Ln^3+^ complexes [Ln^III^{N(Si^i^Pr_3_)_2_}_2_(TEMPO^–^)] (Ln = Tm, **2-Tm**; Yb, **2-Yb**) were synthesized by SET reactions between homoleptic near-linear [Ln^II^{N(Si^i^Pr_3_)_2_}_2_] (Ln = Tm, **1-Tm**; Yb, **1-Yb**)^10^ and TEMPO^•^ in toluene (Scheme 1); **2-Ln** was recrystallized from hexane at −35 °C, and subsequently isolated in yields of 55% for **2-Tm** and 52% for **2-Yb**. Immediate color changes were observed after the addition of TEMPO^•^ to **1-Ln**: a bright orange reaction mixture was observed for Tm and a dark green reaction mixture was observed for Yb. The addition of TEMPO^•^ at −78 °C was found to be crucial to prevent the formation of [Ln^III^(TEMPO^–^)_2_(μ-TEMPO^–^-κ-*O*,*N*)]_2_,^32^ which was identified by X-ray diffraction (XRD) studies of crystals obtained following reactions that were performed at room temperature. The Eu^3+^ analogue **2-Eu** could not be prepared from the reaction of **1-Eu** with TEMPO^•^, with the starting materials identified in the reaction mixture by single crystal XRD; this was attributed to the less negative reduction potential of Eu^2+^ (*E*^θ^ Eu^3+^ → Eu^2+^ = −0.35 V) compared with the other Ln^2+^ ions investigated herein: *E*^θ^ Ln^3+^ → Ln^2+^: −1.15 V (Yb), −1.55 V (Sm), −2.3 V (Tm).^39^

**Scheme 2:**
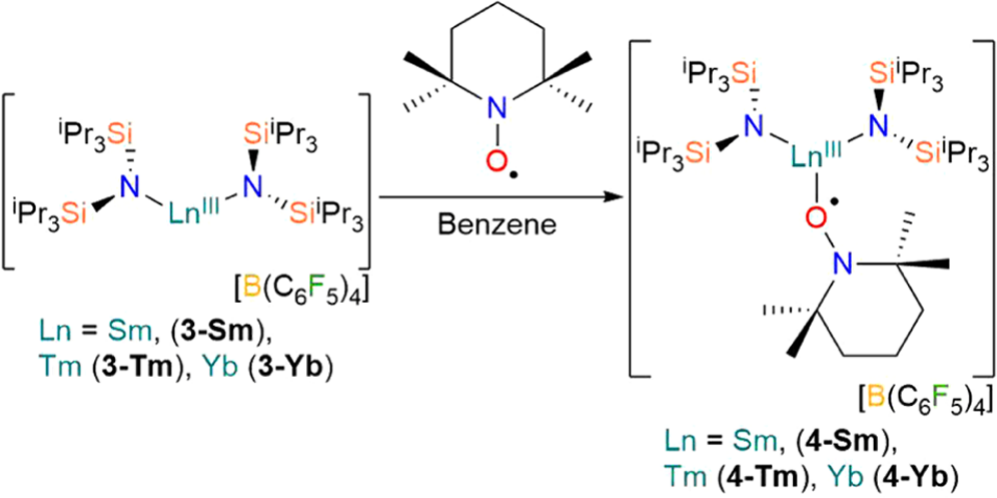
Synthesis of [Ln^III^{N(Si^i^Pr_3_)_2_}_2_(TEMPO^•^)][B(C_6_F_5_)_4_] (**4-Ln**; Ln = Sm, Tm, Yb)

The homoleptic bent Ln^3+^ complexes [Ln^III^{N(Si^i^Pr_3_)_2_}_2_][B(C_6_F_5_)_4_] (**3-Ln**, Ln = Sm, Tm, Yb)^37^ were treated with TEMPO^•^ in benzene to give the heteroleptic three-coordinate Ln^3+^ complexes [Ln^III^{N(Si^i^Pr_3_)_2_}_2_(TEMPO^•^)][B(C_6_F_5_)_4_] (**4-Ln**, Ln = Sm, Tm, Yb) in low yields following recrystallization from DCM (9–66%) (Scheme 2). The elemental analysis results obtained for **2-Tm** and **4-Ln** consistently gave low carbon values; this has frequently been observed for Ln complexes containing {N(Si*^i^*Pr_3_)_2_}, including complexes from the **1-Ln**, **2-Sm**, and **3-Ln** families,^9,10,37^ and as with previous work we attribute this observation to incomplete combustion resulting from the formation of silicon carbides. The FTIR spectra of **2-Ln** and **4-Ln** differ due to the presence of TEMPO^•^ radicals in the latter series; the O–N bond stretching frequency in these complexes are all found to be 1642 cm^–1^, *ca*. 60 cm^–1^ lower than the free radical but comparable to that observed for other Ln complexes containing bound TEMPO^•^ radicals, *e.g.*, [La^III^(hfac)_3_(TEMPO^•^)_2_] (hfac = 1,1,1,5,5,5-hexafluoropentane-2,4-dionate; ṽ = 1649 cm^–1^).^9^

### NMR Spectroscopy

Complexes **2-Ln** and **4-Ln** were probed by multinuclear NMR spectroscopy as *d*_6_-benzene and *d*_2_-DCM solutions, respectively (see Supporting Information (SI) for all spectra). In all cases, ^13^C{^1^H} and ^29^Si DEPT NMR spectra only showed signals from minor diamagnetic impurities due to the paramagnetism of these samples. ^1^H NMR spectra were recorded for **2-Ln** and **4-Ln** from −200 to +200 ppm due to large paramagnetic shifts; the spectra of Tm^3+^ and Yb^3+^ derivatives could not be fully interpreted, though signals for the methyl protons on Si^i^Pr_3_ groups were tentatively assigned in each case due to the large integration of these peaks (72 H) compared to those expected for other proton environments (≤12 H). The assignment of the ^1^H NMR spectra of Sm^3+^ complexes was more straightforward as **4-Sm** gave similar resonances to those reported for **2-Sm** previously^10^ (δ_H_ for **2-Sm**: −5.21 (12 H, C*H*(CH_3_)_2_), −2.68 (2 H, *p*-C*H*_2_), 0.36 (72 H, CH(C*H*_3_)_2_), 2.97 (12 H, C_5_H_6_N*Me*_4_), 3.36 (4 H, *m*-CH_2_)). The [B(C_6_F_5_)_4_]^−^ counterions in **4-Ln** were observed by ^11^B (δ_B_: −16.76, **4-Sm**; −13.25, **4-Tm**; −15.14, **4-Yb**) and ^19^F NMR spectroscopy (δ_F_: −132.80, **4-Sm**; −128.78, **4-Tm**; −130.95, **4-Yb**); the ^11^B resonances of **4-Ln** are similar to those of respective **3-Ln** (δ_B_: −16.76, **3-Sm**; −12.35, **3-Tm**; −14.67, **3-Yb**),^37^ with identical chemical shifts for **3-Sm** and **4-Sm**. Although three signals are expected by ^19^F NMR spectroscopy for the three different fluorine environments only one peak was observed for each **4-Ln** due to paramagnetic broadening; only one signal was previously observed in the ^19^F NMR spectrum of **3-Tm** (δ_F_: −128.51).^37^

### Solid-State Structural Characterization

**Figure 1:**
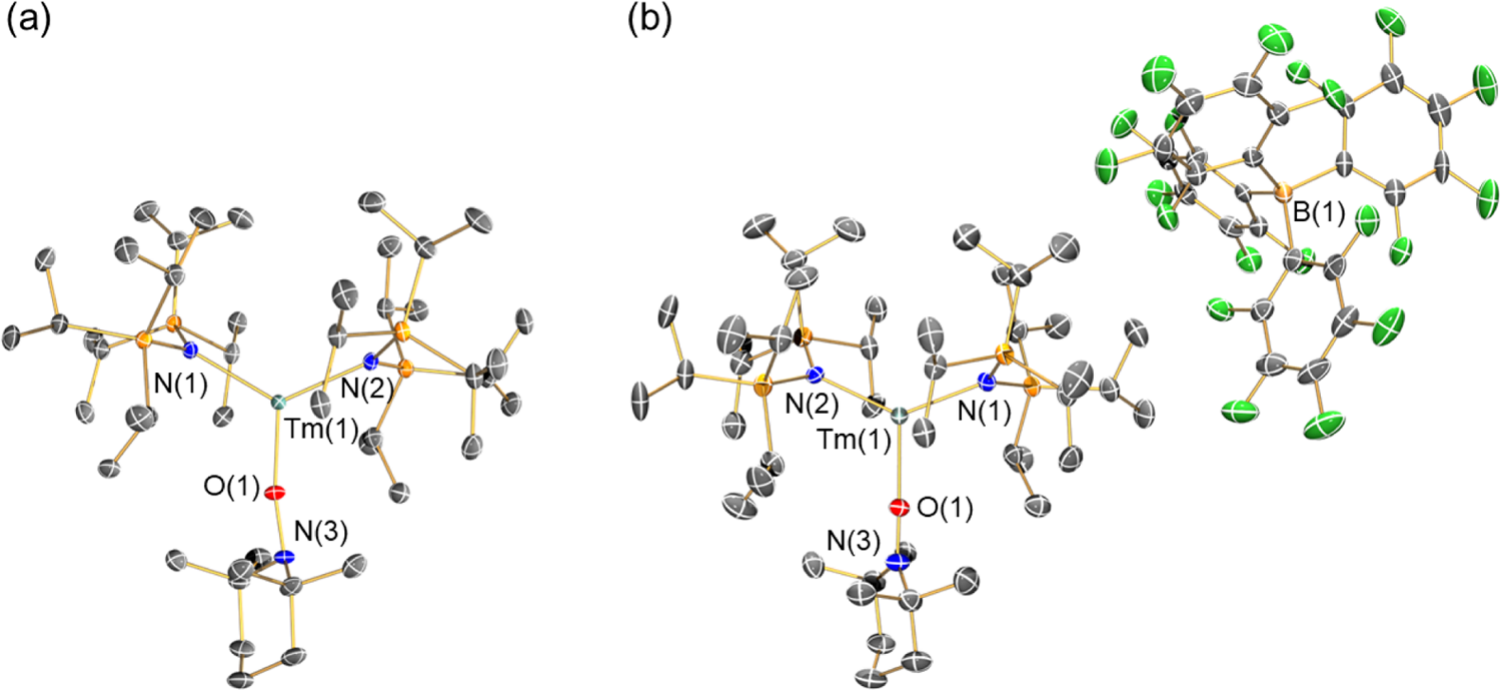
Molecular structures of (a) [Tm^III^{N(Si^i^Pr_3_)_2_}_2_(TEMPO^–^)] (**2-Tm**) and (b) [Tm^III^{N(Si^i^Pr_3_)_2_}_2_(TEMPO^•^)][B(C_6_F_5_)_4_] (**4-Tm**), with selected atom labeling. Displacement ellipsoids are set at a 50% probability level, and hydrogen atoms are omitted for clarity. C = gray, N = blue, O = red, Si = orange, B = yellow, F = green.

The solid-state structures of **2-Ln** and **4-Ln** were determined by single crystal X-ray diffraction; **2-Tm** and **4-Tm** are depicted in Figure 1 (as the **2-Ln** and **4-Ln** families are structurally analogous with each other we focus the discussion on these two complexes for brevity; the other examples are included in the SI and selected metrical parameters are compiled in Table 1). Complexes **2-Tm** and **2-Yb** are isostructural with **2-Sm**;^10^ in the asymmetric unit there is one complete molecule with no local symmetry and half of a molecule with a *C*_2_ axis along the Ln–O bond and the TEMPO moiety disordered around the symmetry axis. We will focus the discussion on molecules without local symmetry. These complexes exhibit approximately trigonal planar geometries, with Ln^3+^ centers located close to the plane defined by the three donor atoms (for **2-Tm**, Tm···N_2_O mean plane: 0.0816(12) Å). The {N(Si^i^Pr_3_)_2_} ligands bend toward each other compared to **1-Ln** precursors to accommodate the coordination of the TEMPO^–^ anion with an oxygen atom: the N1–Ln–N2 bond angles are 125.64(7) and 125.69(10)° in **2-Tm** and **2-Yb**, respectively (*c.f*., 125.07(9)° for **2-Sm**),^10^ and 168.63(6) and 166.01(14)° in **1-Tm** and **1-Yb**, respectively. The N–Tm–O angles for **2-Tm** are 117.84(7) and 116.11(7)°, and the sum of angles about the Tm^3+^ center is 359.6(2)°. The Ln–O1–N3 angles in **2-Ln** (*e.g.*, 167.87(13)° for **2-Tm**) are far closer to linearity than that exhibited in [Y^III^{N(SiMe_3_)_2_}_2_(η^2^-TEMPO^–^)(THF)] (87.60(6)°)^33^ due to the difference in the TEMPO^–^ anion binding mode, which we attribute to the larger steric requirements of the {N(Si^i^Pr_3_)_2_} framework.

**Table 1:** Selected Bond Distances and Angles of [Ln^III^{N(Si^i^Pr_3_)_2_}_2_(TEMPO^–/•^)]^*n*+^ (*n* = 0, 1) Moieties in **2-Ln** and **4-Ln**

complex	Ln–N1/Å	Ln–N2/Å	Ln–O1/Å	O1–N3/Å	N1–Ln–N2/°	N1–Ln–O1/°	N2–Ln–O1/°	Ln–O1–N3/°
**2-Sm**(10)^a^	2.392(3)	2.355(3)	2.086(2)	1.450(4)	125.07(9)	119.62(9)	115.26(10)	169.3(2)
**2-Tm**^a^	2.300(2)	2.268(2)	2.020(2)	1.451(2)	125.64(7)	117.84(7)	116.11(7)	167.87(13)
**2-Yb**^a^	2.285(3)	2.250(3)	2.010(2)	1.448(3)	125.69(10)	117.72(9)	116.23(10)	167.5(2)
**4-Sm**	2.318(2)	2.283(2)	2.338(2)	1.296(2)	130.86(7)	115.39(6)	113.74(6)	170.94(13)
**4-Tm**	2.226(4)	2.220(4)	2.208(3)	1.314(4)	133.33(12)	115.44(12)	111.22(12)	177.7(3)
**4-Yb**	2.207(2)	2.214(2)	2.206(2)	1.305(3)	134.01(9)	115.21(8)	110.77(8)	177.5(2)

a There are 1.5 molecules in the asymmetric unit, values are given for the molecule with no local symmetry.

For **2-Tm**, the mean Tm–N bond distance is 2.284(2) Å; this is shorter than the mean Tm–N bond distance in **1-Tm** (2.374(2) Å),^10^ and consistent with the increased charge of the Tm^3+^ center in **2-Tm**; *c*.*f*. [Tm^III^{N(Si^i^Pr_3_)_2_}_2_(Cl)] (mean Tm–N: 2.229(3) Å).^37^ As expected, the mean Ln–N bond lengths decrease from 2.374(4) Å (**2-Sm**)^10^ to 2.268(4) Å (**2-Yb**), in line with the contraction in Ln^3+^ ionic radii across the Ln series (six-coordinate ionic radii: Sm^3+^, 0.958 Å; Tm^3+^, 0.880 Å; Yb^3+^, 0.868 Å).^40^ Similarly, the Ln–O1 distances in **2-Ln** (2.020(2) Å for Tm and 2.010(2) Å for Yb) are shorter than those in **2-Sm** (2.086(2) Å)^10^ and [Sm^III^(TEMPO^–^)_2_(μ-TEMPO^–^-η^2^-*O*,*N*)]_2_ (2.110(5) and 2.124(6) Å).^32^ The O1–N3 bond lengths (1.451(2) Å for **2-Tm** and 1.448(3) Å for **2-Yb**) are statistically identical to those seen previously in **2-Sm** (O–N: 1.450(4) Å)^10^ and are consistent with TEMPO^–^ anion formulations (O–N covalent bond length: 1.34 Å).^41^ Numerous electrostatic interactions complete the coordination spheres of **2-Ln**; these have been discussed in detail previously for related complexes so will not be commented on further here.^9,10,37^ The anionic TEMPO^–^ ligands in **2-Ln** are further evidenced by the pyramidalization of the N atom compared to the near-planar N environments in the TEMPO^•^ radicals in **4-Ln** (see below).

Complexes **4-Ln** have one crystallographically independent molecule in the unit cell, and it has no local symmetry. In common with **2-Ln**, the Ln^3+^ centers in **4-Ln** are approximately trigonal planar and are coordinated by two N atoms and one O atom; these are even closer to planarity (*e.g.*, for **4-Tm**, Tm···N_2_O mean plane: 0.013(2) Å) but the individual bond angles deviate to a greater extent from 120° (*e.g.*, for **4-Tm**, N1–Tm–N2: 133.33(12)°, N1–Tm–O1: 115.44(12)°, N2–Tm–O1: 111.22(12)°, sum of angles about Tm^3+^: 360.0(4)°) as the Ln^3+^-containing fragments of **4-Ln** are cations and they exhibit TEMPO^•^ radicals rather than TEMPO^–^ anions. Complexes **4-Tm** and **4-Yb** have slightly less bent N1–Ln–N2 angles (133.33(12) and 134.01(9)°, respectively) than **3-Tm** (125.48(9)°) and **3-Yb** (127.7(2)°),^37^ but the N1–Ln–N2 angle for **4-Sm** (130.86(7)°) is not significantly different from that in **3-Sm** (131.02(8)°);^37^ we attribute this observation to the coordination of TEMPO^•^ to the smaller Ln^3+^ centers having a greater steric effect. This trend is matched with the Ln–O1–N3 angles, with small differences between **2-Sm** (169.3(2)°)^10^ and **4-Sm** (170.94(13)°), and larger discrepancies for the smaller Ln^3+^ cations, *e.g.*, **2-Tm** (167.88(13)°) *c.f*. **4-Tm** (177.7(3)°). Although the Ln–O1–N3 angles of **4-Tm** and **4-Yb** are remarkably close to linearity, large variations in these parameters have already been seen in Ln^3+^ TEMPO^•^ radical complexes, *e.g.*, [La^III^(hfac)_3_(TEMPO^•^)_2_] (La–O–N: 146.0(4) and 171.1(4)°),^19^ so we attribute these observations to a combination of steric effects, crystal packing, and dispersion forces, which dominate in effectively electrostatic bonding regimes.^6,7^

As expected, the Ln–O1 bond lengths are longer in **4-Ln** (Sm: 2.338(2) Å, Tm: 2.208(3) Å, Yb: 2.206(2) Å) than their **2-Ln** counterparts (see above), consistent with the reduced electrostatic attraction of TEMPO^•^ radicals vs TEMPO^–^ anions to the Ln^3+^ cations. This is compensated by shorter mean Ln–N distances in **4-Ln** (Sm: 2.301(3) Å, Tm: 2.223(5) Å, Yb: 2.211(3) Å) than those in **2-Ln** (see Table 1). However, the Ln–O bond lengths in **4-Ln** are short compared to those seen in other TEMPO^•^ radical-bound Ln^3+^ complexes, *e.g.*, [Y^III^(hfac)_3_(TEMPO^•^)_2_] (mean Y–O: 2.305(4) Å),^19^ which we attribute to the low formal coordination numbers in **4-Ln**. The O1–N3 bond lengths in **4-Ln** (Sm: 1.296(2) Å, Tm: 1.314(4) Å, Yb: 1.305(3) Å) are shorter than in **2-Ln** (see above), but are similar to those seen in [Y^III^(hfac)_3_(TEMPO^•^)_2_] (mean O–N: 1.296(7) Å),^19^ and the standard O–N covalent bond length (1.34 Å).^41^ Finally, the TEMPO^•^ radicals in **4-Ln** exhibit nearly planar N centers, in contrast to the pyramidalized N atoms in **2-Ln** (*e.g.*, distance N···C_2_O mean plane: 0.078(4) Å for **4-Tm**, 0.420(2) Å for **2-Tm**), providing further structural evidence of TEMPO^•^ radical formulations in **4-Ln**.

### Solution Phase Optical Properties

**Figure 2:**
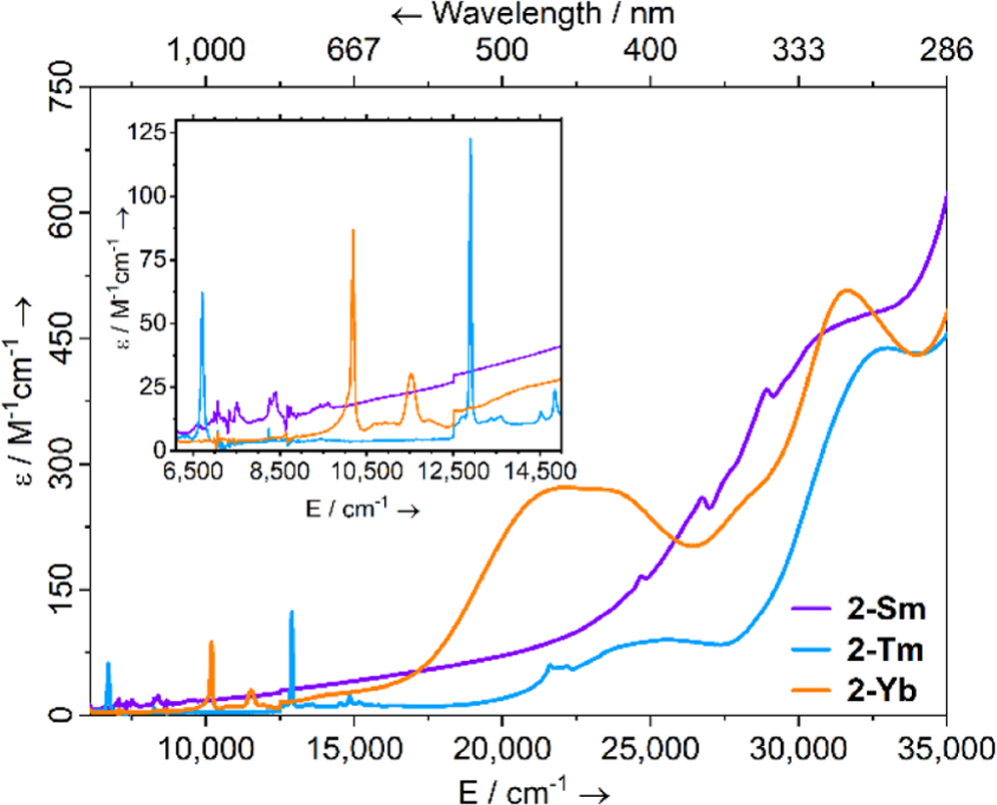
Room temperature UV–vis-NIR spectra of **2-Ln** (1 mM in DCM) from 6100 to 35,000 cm^–1^.

**Figure 3:**
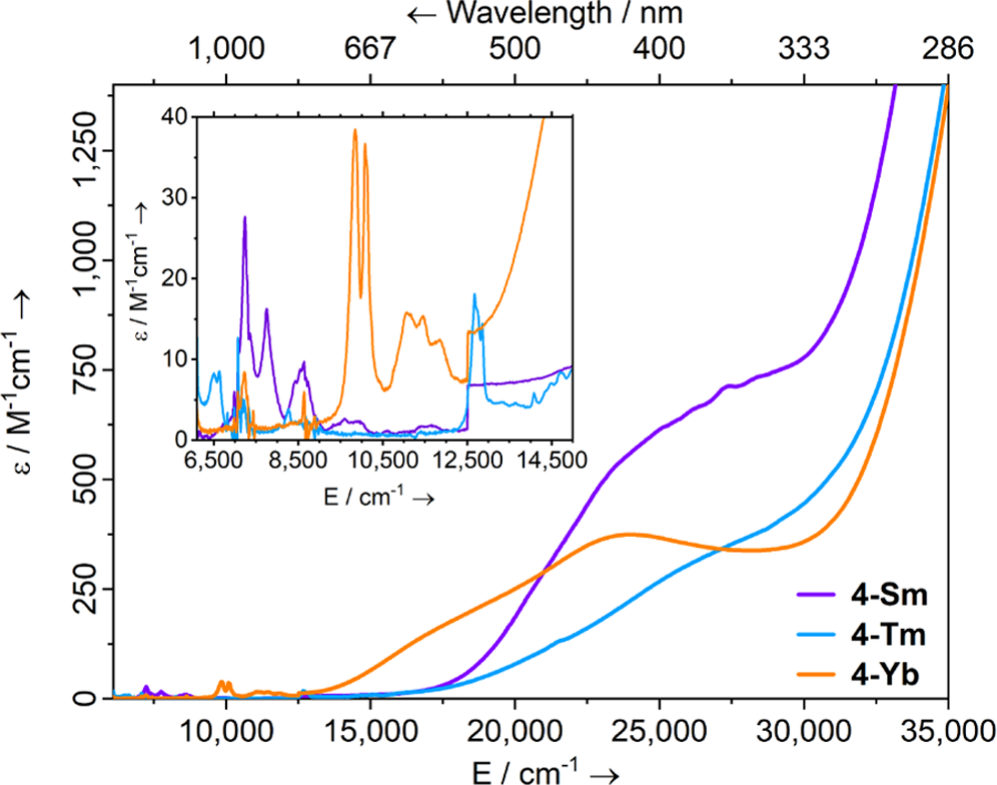
Room temperature UV–vis-NIR spectra of **4-Ln** (1 mM in DCM) from 6100 to 35,000 cm^–1^.

The UV–vis-NIR spectra of **2-Ln** and **4-Ln** were recorded as 1 mM DCM solutions at room temperature (Figures 2, 3, and S21–S26 for individual spectra). Dilute DCM solutions of **2-Sm**, **2-Tm**, and **2-Yb** are red, orange, and green in color, respectively, mainly due to the charge transfer bands tailing into the visible region of the spectra. The bulk features of the spectra are similar to those previously observed for **3-Ln** and [Ln^III^{N(Si^i^Pr_3_)_2_}_2_(X)], (X = Cl, Ln = Sm, Tm; X = F, Ln = Yb).^37,42^ For **2-Sm** one intense absorption [λ_max_ = 308 nm (32,500 cm^–1^), ε = 480 M^–1^ cm^–1^] covers most of the visible region and contains significant fine structure, whereas the visible spectra of both **2-Tm** and **2-Yb** exhibit two clear maxima [**2-Tm**: λ_max_ = 390 nm (26,000 cm^–1^), ε = 90 M^–1^ cm^–1^, λ_max_ = 303 nm (33,000 cm^–1^), ε = 440 M^–1^ cm^–1^; **2-Yb**: λ_max_ = 452 nm (22,000 cm^–1^), ε = 270 M^–1^ cm^–1^, λ_max_ = 316 nm (31,500 cm^–1^), ε = 505 M^–1^ cm^–1^]. For all **2-Ln**, weak absorptions were observed in the near-IR region corresponding to the Laporte forbidden f–f transitions; these are spin-allowed and relatively strong (ε < 200 M^–1^ cm^–1^).^2^ The spectrum of **2-Sm** has multiple peaks arising due to the ^6^H_5/2_ → ^6^F*_J_* transitions.^2^ The spectrum of **2-Tm** has two sharp peaks at λ_max_ = 1490 nm (6700 cm^–1^), ε = 60 M^–1^ cm^–1^ and λ_max_ = 775 nm (13,000 cm^–1^), ε = 120 M^–1^ cm^–1^, and two regions of smaller peaks with λ_max_ = 659–673 nm (14,500–15,200 cm^–1^), ε = 15–25 M^–1^ cm^–1^ and λ_max_ = 451–463 nm (21,600–22,200 cm^–1^), ε = 55–60 M^–1^ cm^–1^; these correspond to ^3^H_6_ → ^3^H_4_, ^3^H_6_ → ^3^F_4_, ^3^H_6_ → ^3^F_3_ and ^3^H_6_ → ^1^G_4_ transitions. **2-Yb** has two absorptions at λ_max_ = 981 nm (10,200 cm^–1^), ε = 85 M^–1^ cm^–1^ and λ_max_ = 868 nm (11,500 cm^–1^), ε = 30 M^–1^ cm^–1^ arising due to ^2^F_7/2_ → ^2^F_5/2_ transitions, which have been split by a large CF of over 1000 cm^–1^.

In common with **2-Ln**, the visible part of the electronic spectra for **4-Ln** is dominated by charge transfer bands (Figure 3). For **4-Sm** and **4-Tm** large shoulders are observed [**4-Sm**: λ_max_ = 364 nm (27,500 cm^–1^), ε = 710 M^–1^ cm^–1^; **4-Tm**: λ_max_ = 370 nm (27,000 cm^–1^), ε = 365 M^–1^ cm^–1^] that tail in from the UV region, and for **4-Yb** a broad peak at λ_max_ = 416 nm (24,000 cm^–1^), ε = 375 M^–1^ cm^–1^ is seen. Laporte forbidden f–f transitions are also observed for **4-Ln** in the near-IR region that are similar to those seen for their **2-Ln** partners: **4-Sm** exhibits three peaks at ṽ ∼ 7200, 7750, and 8600 cm^–1^ which correspond to the ^6^H_5/2_ → ^6^F*_J_* transitions, and are in good agreement with the [Sm^III^{N(Si^i^Pr_3_)_2_}_2_(X)] (X = F, Cl, Br, I) series;^42^**4-Tm** exhibits two main peaks at λ_max_ = 789 nm (12,500 cm^–1^), ε = 15 M^–1^ cm^–1^ and λ_max_ = 1508 nm (6600 cm^–1^), ε = 5 M^–1^ cm^–1^, a group of peaks 659–710 nm (14,100–15,200 cm^–1^), ε = 5–10 M^–1^ cm^–1^, and a shoulder around 462 nm (21,600 cm^–1^) that correspond to the ^3^H_6_ → ^3^H_4_, ^3^H_6_ → ^3^F_4_, ^3^H_6_ → ^3^F_3_, and ^3^H_6_ → ^1^G_4_ transitions.^2^ These transitions are weaker and more split than those in **2-Tm**. The near-IR region of the **4-Yb** spectrum has more transitions than that expected for ^2^F_7/2_ → ^2^F_5/2_. This is attributed to an equilibrium of **4-Yb** and starting material **3-Yb**, formed by the dissociation of the TEMPO^•^ radical in DCM solution, where **3-Yb** is responsible for peaks at λ_max_ = 1015 nm (9850 cm^–1^), 903 nm (11,070 cm^–1^) and 843 nm (11,860 cm^–1^; Figure S26). The intense absorption at λ_max_ = 992 nm (10,080 cm^–1^), ε = 35 M^–1^ cm^–1^, and the weaker absorption at λ_max_ = 873 nm (11,460 cm^–1^), ε = 15 M^–1^ cm^–1^, are ^2^F_7/2_ → ^2^F_5/2_ transitions belonging to **4-Yb**, split by a ^2^F_5/2_ CF of over 1000 cm^–1^. The energies of the f–f transitions in **2-Ln** and **4-Ln** are similar, indicating that the ligand fields are of comparable strength in the two series of complexes.

### EPR Spectroscopy

**Figure 4:**
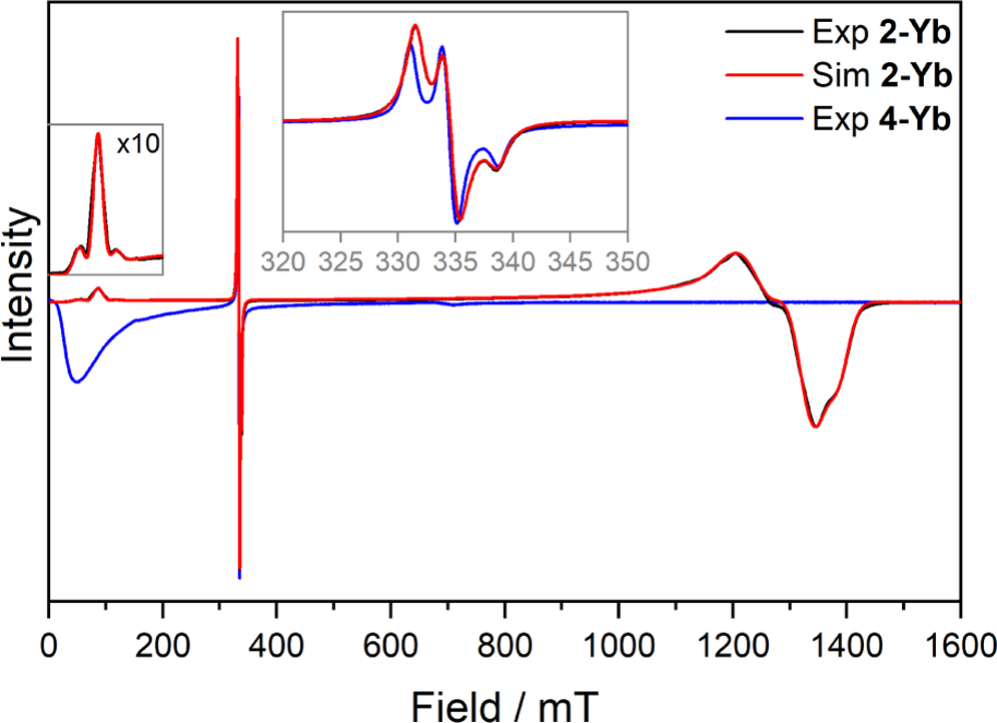
X-band EPR spectrum of **2-Yb** (black, 8 K, ν = 9.389641 GHz) with the simulated spectrum using parameters specified in the text and Table S3 (red) and spectrum of **4-Yb** (blue, 9 K, ν = 9.384739 GHz). Insets show low field region and radical region.

X-band (9.38 GHz) EPR spectra were recorded on powders of **2-Ln** and **4-Ln** at 6–9 K (see SI for full details). Complexes **2-Tm**, **2-Sm**, **4-Tm**, and **4-Sm** were EPR silent, except for a small organic radical impurity present in **2-Tm** and **4-Tm**. For **2-Yb**, a broad *S* = 1/2 spectrum was observed, including a *g*_1_ feature with a hyperfine structure at 90 mT and *g*_2_ and *g*_3_ features around 1150–1450 mT; the spectrum was overlaid with a sharp *S* = 1/2 feature, with hyperfine structure and centered at 334.5 mT, characteristic of a TEMPO^•^ radical (Figure 4). In Kramers ions such as Yb^3+^, all states are doubly degenerate, so the ground doublet can be considered as an effective spin 1/2 with three anisotropic *g*-values (*g*_1_ > *g*_2_ > *g*_3_). The spectrum was simulated in *EasySpin*,^43^ using a model of two anisotropic *S*_eff_ = 1/2 in a 2:1 ratio to represent the ratio of crystallographically unique **2-Yb** molecules in the unit cell, each with ^171^Yb/^173^Yb hyperfine splitting, and a 0.28% TEMPO^•^ radical impurity with ^14^N hyperfine splitting. The ^173^Yb hyperfine values were fixed based on their *g*-values,^44^ with ^171^Yb hyperfine values scaled from the ^173^Yb values according to their nuclear *g*-factors.^43^ Simulation determined *g*-values of 7.755, 0.540, and 0.499 for one molecule and 7.755, 0.512, and 0.484 for the second molecule (*g*_1_ restrained to be equal for both molecules as no splitting is observed within resolution). These Yb^3+^ environments are pseudoaxial, with an easy magnetization axis along *g*_1_ and a hard plane defined by *g*_2_ and *g*_3_; *g*_2_ ≈ *g*_3_ reflecting the trigonal planar coordination environment. The *g-*values are similar to those expected for a pure *m*_J_ = |±7/2⟩ (*g*_1_ = 8, *g*_2_ = *g*_3_ = 0), indicating that this is the major component of the ground state. For **4-Yb**, a very broad feature near zero field (Figure S28) suggests two exchange states separated by ∼0.32 cm^–1^ in the absence of an applied field. A sharp peak corresponding to a TEMPO^•^ radical impurity is also observed at *g* = 2.00. There are several other smaller features (Figure S29) most likely arising from decomposition products, with a peak at *g* = 7.5 similar to that of **2-Yb**, and additional peaks at 4.4 and 0.97 likely arising from ferrocenium impurity present in the powder sample.

### Magnetism

**Figure 5:**
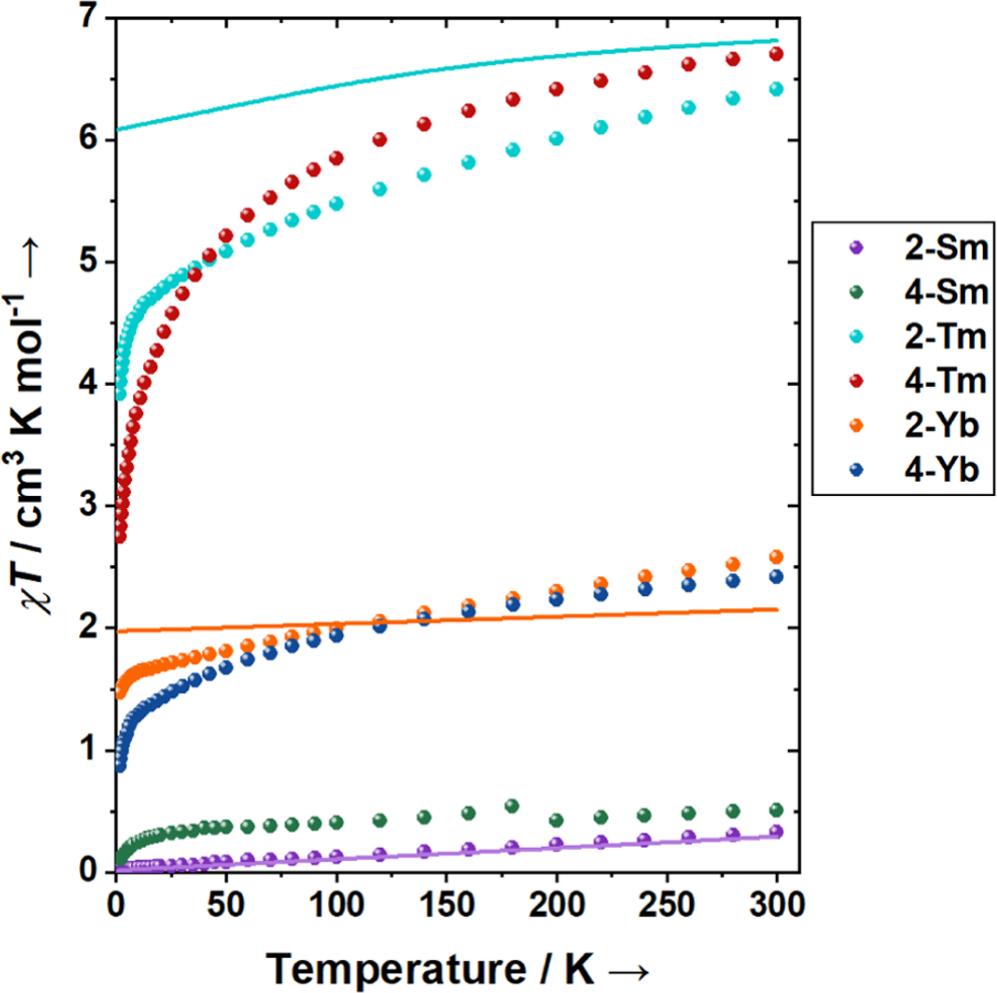
Temperature-dependence of χ*T* for **2-Ln** and **4-Ln** with calculated CASSCF (**2-Sm**, **2-Tm**) or XMS-CASPT2 (**2-Yb**) curves (solid line).

Complexes **2-Ln** and **4-Ln** were characterized by SQUID magnetometry to determine their electronic structure and the effect of switching the TEMPO^–^ anion to a TEMPO^•^ radical (see SI for full details). The samples **2-Ln** and **4-Ln** are highly sensitive to air; small errors in the diamagnetic corrections and the residual field in the instrument, as well as any remaining cocrystallized solvent, sample decomposition or impurities may affect the magnetic data (magnetization, χ*T* and Δχ*T*, see below). However, the contribution of the EPR-quantified radical impurity (∼0.28%) is too small to be observed by magnetometry. Room temperature values of the product of magnetic susceptibility and temperature (χ*T*) for **2-Ln** are within the expected ranges for their respective Ln^3+^ ions (Table 2). The χ*T* values for **2-Ln** linearly decrease with decreasing temperature (Figure 5) as the ground-term CF states, and low-lying ^6^H*_J_* and ^6^F*_J_* states for **2-Sm**, are depopulated. These ground terms are ^6^H_5/2_ (**2-Sm**), ^3^H_6_ (**2-Tm**), and ^2^F_7/2_ (**2-Yb**). Below 10 K for **2-Tm**, there is a more rapid decrease in χ*T*, suggesting a split ground pseudodoublet, which is nondegenerate as Tm^3+^ is a non-Kramers ion.

**Table 2:** Room Temperature χ*T* Values for **2-Ln** and **4-Ln** Determined by Solid-State SQUID Magnetometry, with Free-Ion Values [*g_J_*^2^*J*(*J* + 1)/8], and Values from CASSCF or XMS-CASPT2 Calculated Electronic Structures

χ*T*/cm^3^ mol^–1^ K	**2-Sm**	**2-Tm**	**2-Yb**	**4-Sm**	**4-Tm**	**4-Yb**
free-ion^a^	0.09^b^	7.15	2.57	0.46^b^	7.52	2.95
SQUID	0.32	6.41	2.58	0.51	6.70	2.41
CASSCF	0.29	6.81	2.21/2.15^c^	0.73	7.28	2.56
typical values^a^	0.28–0.32	6.30–6.85	2.42–3.00	0.66–0.70	6.68–7.22	2.80–3.38

a Values for **4-Ln** calculated as the sum of metal and radical contributions, assuming no coupling.

b Theoretical value for ground spin–orbit multiplet in the absence of a ligand field.

c XMS-CASPT2 value.

**Figure 6:**
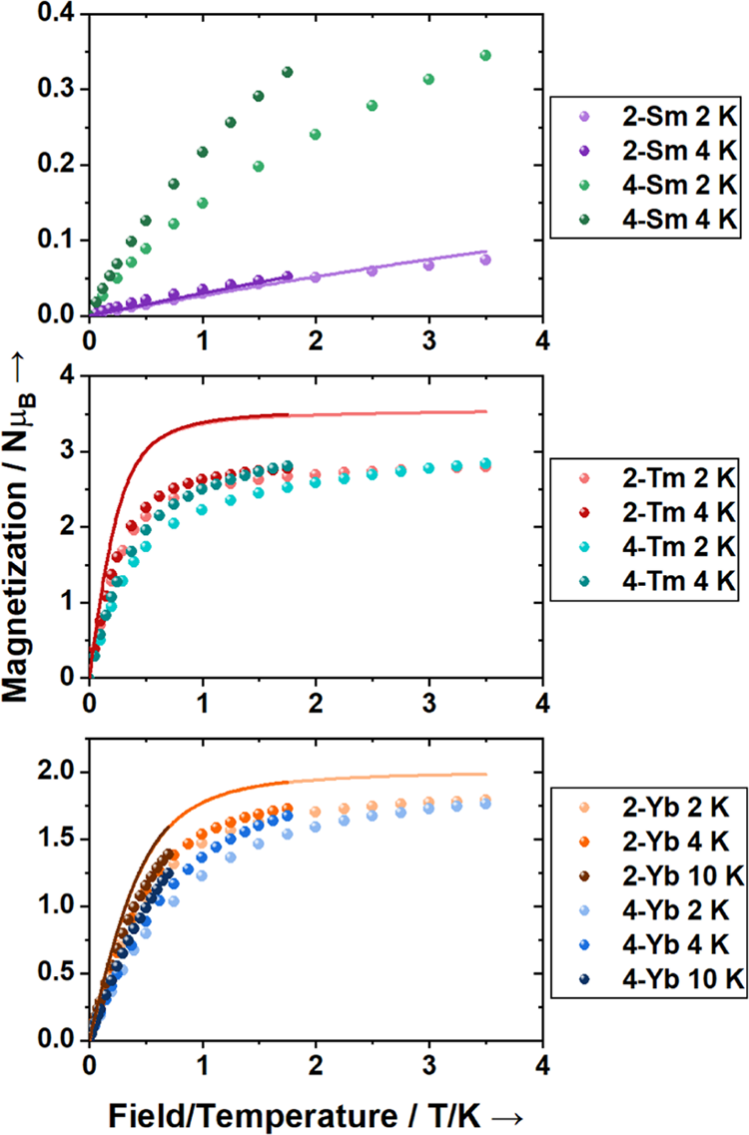
Isothermal reduced magnetization plots for **2-Ln** and **4-Ln** with CASSCF (**2-Sm**, **2-Tm**) or XMS-CASPT2 (**2-Yb**) calculated curves (solid lines).

Examination of the reduced magnetization plots (magnetization vs. field/temperature) provides information about the ground state (Figure 6). For all **2-Ln**, the isothermal reduced magnetization curves overlay, indicating a well-isolated ground state. For **2-Tm** and **2-Yb**, the ground states are highly magnetic, showing sharp rises at low fields and reaching magnetic saturation, with *M*_sat_ values of 2.80 and 1.79 Nμ_B_, respectively. The saturation values are slightly less than for pure *m*_*J*_ = |±6⟩ (3.50 Nμ_B_) or *m*_*J*_ = |±7/2⟩ (2.00 Nμ_B_) indicating some mixing in these states.

The room temperature χ*T* values for **4-Ln** are expected to be the sum of the Ln^3+^ contribution and a spin 1/2 (0.375 cm^3^ K mol^–1^), as Ln-radical exchange coupling is expected to be weak and hence negligible at room temperature.^15^ As there is a change in formal charge on the coordinating TEMPO^•/–^ oxygen atom and there are small changes in the geometry of the [Ln^III^{N(Si^i^Pr_3_)_2_}_2_(TEMPO^–/•^)]^0/+^ species, the Ln^3+^ CF is expected to be modified from **2-Ln** to **4-Ln**, so the difference in χ*T* (Δχ*T*) may differ by more or less than the radical contribution even if the effect of exchange is minor. Smaller errors, as mentioned above, may also affect Δχ*T*. For **4-Sm**, the room temperature χ*T* is slightly below what is expected for [Sm^3+^ + radical], and the Δχ*T* between **2-Sm** and **4-Sm** is +0.19 cm^3^ K mol^–1^ at 300 K (Table 2), and approximately constant from 50–300 K (Figure S29). Metal-radical coupling takes effect below 50 K, where χ*T* rapidly decreases as the exchange states are depopulated (Figure 5). The isothermal reduced magnetization curves do not overlay for **4-Sm**, confirming the presence of low energy exchange states from the coupling of Sm^3+^ with the TEMPO^•^ radical. The lowest two exchange states are likely split by >0.32 cm^–1^ as **4-Sm** is EPR silent.

For **4-Tm**, the room temperature χ*T* value of 6.70 cm^3^ K mol^–1^ is within the expected range for [Tm^3+^ + radical] (Table 2). The difference between **4-Tm** and **2-Tm** is nonlinear but centered around +0.375 cm^3^ K mol^–1^ between 100–300 K Figure S29, with a value of +0.29 cm^3^ K mol^–1^ at room temperature, again supporting that the CF is modified going from **2-Tm** to **4-Tm**. Here, the exchange coupling is evident below 100 K, so it is likely larger than in **4-Sm** consistent with a larger dipolar interaction with the more magnetic Tm^3+^ ion. The nonoverlapping reduced magnetization indicates low energy exchange states, but the magnetization data could not be fit with an Ising-like coupling between the effective spin 1/2 of the pseudodoublet and the radical *S* = 1/2 (Figure S31). We also attempted to simulate the χ*T* and magnetization curves using a Lines model of exchange between the full *S* = 1, *L* = 5 of Tm^3+^, using crystal field parameters defined by CASSCF calculations defined on the electronic structure without the radical (**[4-Tm]**^**0**^, see below), and the radical *S* = 1/2 with either isotropic or Ising (*J*_*z*_) exchange (Figures S32 and S33) but these were unable to reproduce the experimental data.

For **4-Yb**, the room temperature χ*T* value of 2.41 cm^3^ K mol^–1^ is less than expected for [Yb^3+^ + radical], and less than the value for **2-Yb** (Table 2); this anomalously low value may warrant further investigation in the future. The χ*T* value for **4-Yb** gradually decreases below 50 K and rapidly decreases below 7 K, suggesting antiferromagnetic coupling. The isothermal reduced magnetization plots have a large initial magnetization slope and a magnetization approaching saturation at high field and do not overlap (Figure 6), suggesting closely spaced exchange states populated at low temperature. This is consistent with exchange states separated by ∼0.32 cm^–1^ as observed in the EPR spectrum. As all magnetization isotherms for **4-Yb** are lower than those for **2-Yb**, this again supports an antiferromagnetic exchange interaction. Reproducing the magnetization data with isotropic coupling or Ising-like coupling between the effective spin 1/2 of the Kramer’s doublet and the radical *S* = 1/2 was not possible (Figures S34 and S35). Simulation of the χ*T* and magnetization curves was performed using a Lines model of exchange between the full *S* = 1/2, *L* = 3 of Yb^3+^, using crystal field parameters defined by CASSCF calculations defined on the electronic structure without the radical (**[4-Yb]**^**0**^, see below), and the radical *S* = 1/2 with either isotropic or Ising (*J*_*z*_) exchange (Figures S36 and S37). While a reasonable simulation of the magnetization could be obtained with *J*_*z*_ = −19.8 cm^–1^, the temperature-dependence of χ*T* could not be reproduced.

None of **2-Tm**, **4-Tm**, **2-Yb**, or **4-Yb** display peaks in the out-of-phase magnetic susceptibility (χ″) in zero field (1–1000 Hz) and so are not SMMs (Figures S38–S41). **2-Sm** and **4-Sm** were not investigated because of their weakly magnetic ground states. However, **4-Yb** is slow to equilibrate in field, showing a divergence of the zero-field-cooled (ZFC) and field-cooled (FC) susceptibilities below 44 K (Figure S42, peak in ZFC trace at 26 K) and a spindle-shaped hysteresis at 2 K (Figure S43), in contrast to **2-Yb** which shows no slow magnetization dynamics despite the equatorial coordination environment. We further investigated the dynamic behavior of **4-Yb** in a 1 kOe applied dc field and observed frequency dependence of χ″ only at 2 K and below with no observable peaks in χ″ in the observable frequency range (Figure S44). We attribute the high-temperature divergence of ZFC/FC to slow thermalization,^45^ which was observed directly during measurements, while the slow relaxation in **4-Yb** at 2 K and 1 kOe occurs within the ground exchange states (splitting ∼0.32 cm^–1^).

### Computational Characterization

To better understand magnetic, optical and EPR data, complete active space self-consistent field (CASSCF) calculations were performed on **2-Ln** in OpenMolcas^46,47^ using the solid-state XRD structures above (see SI for details). An active space of 7 × 4f orbitals was used for **2-Ln**.

**Figure 7:**
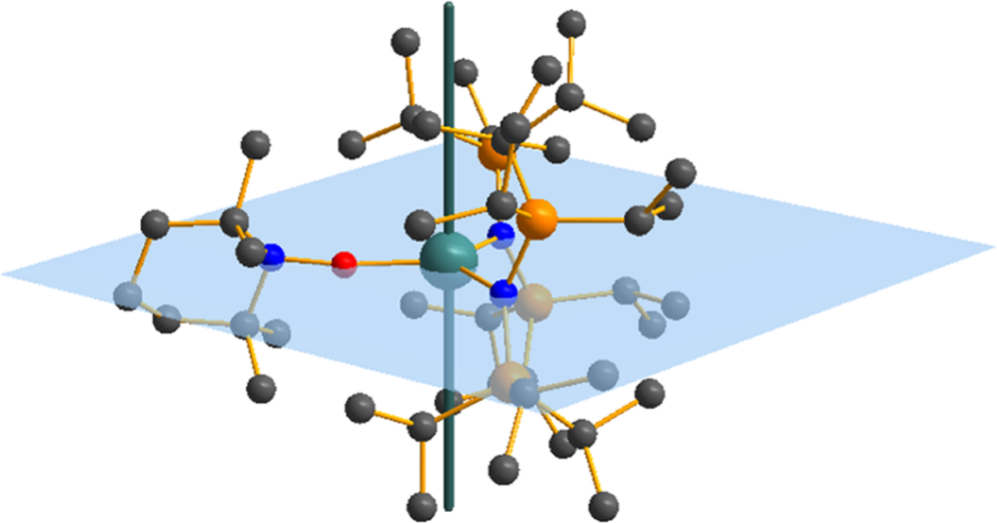
Molecular structure of **2-Yb** showing the *g*_1_ main magnetic axis calculated with XMS-CASPT2 (green) and plane through N_2_O coordinating atoms (pale blue).

CASSCF calculations on **2-Yb** predict an almost pure ground state of 99.93% *m*_*J*_ = |±7/2⟩ which is stabilized by >1000 cm^–1^ (Table 3). The ground state composition agrees with EPR data; however, transverse *g*-values are underpredicted (<0.09). Including dynamic correlation effects with extended multistate complete active space second-order perturbation theory (XMS-CASPT2) reproduces the magnetic data (Figures 5 and 6), and gives calculated *g*-values of 7.927, 0.336 and 0.324, that are closer to the experimental values of 7.755, 0.512–0.540, 0.484–0.499 (Table 3).^48,49^ The XMS-CASPT2 method was used over the regular MS-CASPT2 as it behaves better in the case of near-degenerate states, present in Ln^3+^ complexes.^50,51^ As expected for an equatorial coordination motif,^3^ the ground state is dominated by prolate *m*_*J*_ = |±7/2⟩ (99.7%) with the *g*_1_ quantization axis almost perpendicular (0.6° to normal) to the ON_2_ plane (Figure 7). The mixing in the ground state and large transverse *g*-values underpin the absence of slow relaxation in **2-Yb**, despite the highly magnetic ground state being stabilized by 1400 cm^–1^. The large CF is echoed in the ^2^F_5/2_ states, which are calculated at 10,500/10,600 cm^–1^ (CASSCF/XMS-CASPT2), 11,700/12,100 and 12,100/12,500 cm^–1^ for *m*_*J*_ = |±5/2⟩, |±3/2⟩, and |±1/2⟩ (Table S6), in excellent agreement with the observed transitions.

**Table 3:** Selected Computational Results for **2-Ln**

		ground (pseudo)-doublet				
	method	*g*_1_	*g*_2_	*g*_3_	composition	first excited state (cm^–1^)
**2-Yb**	CASSCF	7.963	0.087	0.085	99.93% |±7/2⟩	1054
**2-Yb**	XMS-CASPT2	7.927	0.336	0.324	99.7% |±7/2⟩	1400
**2-Tm**	CASSCF	13.95			49.98% |±6⟩ + 49.98% |∓6⟩	393
**2-Sm**	CASSCF	0.645	0.043	0.011	n/a^a^	636

a *J* is a poor quantum number to use due to mixing with low-lying ^6^H_7/2_ and ^6^H_9/2_ multiplets

For non-Kramers **2-Tm**, CASSCF predicts a highly pure *m*_*J*_ = 49.98% |±6⟩ + 49.98% |∓6⟩ ground pseudodoublet split by 0.043 cm^–1^ and stabilized by 393 cm^–1^. The ground state has *g*_1_ = 13.95 (*g*_1_ = 14 for pure *m*_*J*_ = |±6⟩, Table 3) orientated perpendicular to the coordination plane (0.3° to normal, Figure S48). Calculations slightly overestimate the room temperature χ*T* value (Figure 5) and replicate a gradual decrease in χ*T* from 300–10 K but do not reproduce the sharp downturn below 10 K or *M*_sat_ (Figure 6). The low-temperature drop observed experimentally indicates a nondegenerate ground state in **2-Tm** (χ*T* should drop to 0 for singlet ground state). The ground state splitting is underestimated by computational methods (0.043 cm^–1^), while the absence of an EPR signal for **2-Tm** suggests the pseudodoublet is split by >0.32 cm^–1^. Although we note that a highly pure doublet ground state could also be EPR silent. Computational methods are unable to achieve this sub-wavenumber level of accuracy and likely underestimate the level of mixing in the ground state. XMS-CASPT2 was computationally prohibitive due to the large number of states for Tm^3+^ in an already large molecule, the computational limits were already being reached for **2-Yb**. Intermolecular interactions are not included in these calculations and could also impact the low temperature behavior and agreement with magnetic data for both **2-Tm** and **2-Yb**.

CASSCF calculations on **2-Sm** indicate a ground state with *g*-values of 0.645 (0.6° to normal of N_2_O coordination plane), 0.043 and 0.011 (Figure S49). Static magnetic data are very well reproduced (Figures 5 and 6) by CASSCF, which also confirms many low-lying ^6^H and ^6^F states up to 14,000 cm^–1^, as observed in UV–vis-NIR spectroscopy (Table S10). The ^6^H_7/2_ states in particular are very low in energy, with the third and fourth Kramer’s doublets separated by only 110 cm^–1^. This results in significant *J*-mixing, such that *J* is a poor quantum number for this complex and the *g*-values of the lowest three states are changed from those expected in a ^6^H_5/2_ multiplet.^52^

We aimed to use CASSCF calculations to provide information about the exchange spectra of **4-Ln**, mainly focusing on **4-Yb**. However, *ab initio* calculations on the cations of **4-Ln** did not accurately model the low-temperature magnetic data for **4-Yb** and **4-Tm** (Figures S46 and S47), indicating an incorrect exchange spectrum. These attempts are detailed in the SI.

CASSCF calculations on **4-Ln** using an active space of 7 × 4f orbitals and one TEMPO^•^ N–O π* orbital (Figure S45) were able to provide good reproductions of UV–vis-NIR data and CF splitting, as the energy scale is much larger than the exchange interactions. For **4-Yb**, ^2^F_7/2_ → ^2^F_5/2_ transitions are predicted at 10,500, 11,400, and 12,200 cm^–1^ (Table S6), in excellent agreement with experiment (10,080 and 11,460 cm^–1^) and showing a similar splitting due to the CF as **2-Yb**. The energies of excited ^3^H_4_ and ^3^F_4_ states seen in UV–vis-NIR spectra are confirmed to be virtually unchanged from **2-Tm** to **4-Tm** (Table S8). The near-continuum of excited ^6^H- and ^6^F-derived states up to 13,500 cm^–1^ are also reproduced for **2-Sm** and **4-Sm** (Table S10). Furthermore, the states derived from the Yb^3+^^2^F_7/2_ and Tm^3+ 3^H_6_ ground terms span a similar energy range in **2-Ln** and **4-Ln**. Comparison to calculations using the coordinates of the **4-Ln** cations but treated as a neutral species with artificial Ln^3+^-TEMPO^–^ charge distributions (**[4-Ln]**^**0**^) can distinguish between the effects of changing the geometry and changing the TEMPO^–^ anion to a TEMPO^•^ radical.^53^ Comparing **2-Ln** and **[4-Ln]**^**0**^ indicates geometry changes contribute only a minor increase in mixing and do not affect the approximate energies or ordering of the states; most CF parameters are changed by less than 1 order of magnitude (Table S11). The introduction of the radical (**4-Ln** vs **[4-Ln]**^**0**^) has a more significant effect on the CF: the energy of the first excited exchange multiplet is lowered by 18% for Sm and Tm and 15% for Yb.

## Discussion

The isolation of the series of Ln^3+^ complexes **2-Ln** and **4-Ln** has allowed for a structural study comparing the TEMPO^–^ and TEMPO^•^ ligands in nearly identical coordination environments. The structural analysis has revealed the subtle changes expected for the coordination of the ligand in different oxidation states and specifically highlighted the near-linear Ln–O–N coordinate bond in **4-Ln**. The impact of the altered coordination on the Ln ions was investigated through CASSCF/CASPT2 calculations employed to the **2-Ln** and **[4-Ln]**^**0**^ structures and revealed remarkably similar overall electronic structures. Despite the increase in the Ln–O bond lengths between **2-Ln** and **4-Ln** pairs, the commensurate decrease in the Ln–N bonds results in a comparable CF environment in the artificial **[4-Ln]**^**0**^ systems. While attempts were made to directly compare the electronic structure between **2-Ln** and **4-Ln**, the inconsistent nature of the CAS(X,8) tests does not allow for the meaningful study of the exchange spectrum. Nevertheless, weak exchange between the Ln and radical electrons is expected.^19,54^ The first excited multiplet is at a lower energy in **4-Ln** compared to **[4-Ln]**^**0**^, consistent with the overall CF contribution being reduced in **4-Ln** by the weaker donor nature of the TEMPO^•^.

The magnetic measurements for **2-Ln** and **4-Ln** were as expected at room temperature when accounting for contributions from the Ln and radical (where appropriate) with **4-Yb** being the only outlier. The degree of exchange strength can be approximated by comparison of the low temperature drop in χ*T*, with |*J*_Tm_| > |*J*_Yb_| ≈ |*J*_Sm_|, in agreement with the dipolar coupling strength expected from the magnetic moment for the given *J* terms (*g*_*J*_·*J* = 7 > 4 > 5/7). Exchange interactions in **4-Yb** are antiferromagnetic and result in ground exchange states separated by ∼0.32 cm^–1^. Exchange interactions are estimated to be no more than tens of wavenumbers, but magnetic data could not be reproduced with simple exchange models, consistent with a recent INS study on an Er-radical mononuclear complex that found a complex exchange interaction involving a high level of mixing between Ln 4f and radical-π electrons.^54^ The simple exchange models may also have failed because of an inaccurate crystal field in the absence of the radical, consistent with the poor reproduction of **2-Yb** and **2-Tm** low temperature magnetic data by calculations and a change in the crystal field with a negative vs. neutral TEMPO ligand. Simple scaling of the CF parameters does not improve the **4-Ln** simulations, suggesting a more complex change in the crystal field.

The relatively large CFs in **2-Yb** and **2-Tm** result from the well-defined equatorial coordination environment of **2-Ln** stabilizing the prolate electron density. Particularly important are the short out-of-plane Ln···N_2_O distances (*e.g.*, **2-Yb**: 0.075(2) Å), which result in a higher energy first excited state and overall CF splitting for **2-Yb** (CASSCF/CASPT2:1054/1400 and 1853/2296 cm^–1^) than the related trigonal pyramidal complex [Yb^III^{N(SiMe_3_)_2_}_3_] (768 and 1464 cm^–1^ by luminescence) which has a Yb···N_3_ distance of 0.556(1) Å.^55^ As has been a common trend in the literature, magnetic data reveal another Yb^3+^ equatorial coordination complex that does not demonstrate magnetic memory effects.^55−60^ The lack of SMM behavior is consistent with computational calculations and EPR experimental data, both of which indicate significant transverse *g*-values and rapid QTM rates within the ground Kramers doublet. Recent work allows for an approximation of τ_QTM_ based on the ground state *g*-values and here yields τ_QTM_ ≈ 1 × 10^–7^ s (based on CASPT2 *g*-tensor with 20 mT average field,^61^ τ_QTM_ ≈ 2 × 10^–6^ s from CASSCF *g*-tensor), far faster than observable with ac susceptibility on PPMS or MPMS systems. The aforementioned compound [Yb^III^{N(SiMe_3_)_2_}_3_] displays slow relaxation in an applied field up to 9 K *via* Raman and QTM processes;^55^ we attribute the contrasting lack of in-field SMM behavior in **2-Yb** to the lower symmetry. These results highlight the importance of minimizing transverse *g*-values, regardless of the magnitude of the overall CF splitting.

Coupling of a radical to the Ln ion often shifts QTM away from zero field, resulting in switched-on or improved SMM behavior.^15^ Alternative examples exist, including a tetrahedral Er(III) complex, [Er(TTBP)_3_(THF)] (TTBP = 2,4,6-tri-*tert*-butyl-phenolate) which loses SMM behavior when the THF is substituted with TEMPO^•^.^24^ While **4-Yb** shows evidence of slow relaxation in contrast to **2-Yb**, it is not a result of exchange biasing because the hysteresis is spindle-shaped and still closed at zero field. We conclude that there are relaxation processes other than QTM that prevent **2-Yb** and **4-Yb** from being SMMs. The hysteresis and frequency dependence of the ac susceptibility under the 1 kOe dc field in **4-Yb** are attributed to processes occurring within the exchange states.

## Conclusions

A series of approximately trigonal planar heteroleptic Ln^3+^ bis(silyl)amide complexes containing either TEMPO^•^ radicals or TEMPO^–^ anions have been synthesized and analyzed. The ideal equatorial coordination geometry of **2-Ln** greatly stabilizes pure, magnetic ground states with prolate electron density in **2-Yb** (>1000 cm^–1^) and **2-Tm** (>390 cm^–1^). Stabilization of the ground state is reduced as the charge of the anionic coordination sphere decreases in **4-Ln**. Despite the idealized geometry in **2-Yb**, dynamic correlation effects contribute to large transverse *g*-values and the absence of slow magnetic relaxation. The introduction of the radical in **4-Ln** switched on slow relaxation but without peaks in the out-of-phase susceptibility under a 1 kOe dc field and with the 2 K hysteresis loop remaining closed in the important zero field region. Exchange interactions are no more than tens of wavenumbers in **4-Ln**, affecting magnetic data below 50–100 K; however, CASSCF and simple exchange models are unable to reproduce the exchange coupling in **4-Ln** accurately. Magnetization and EPR measurements indicate closely spaced exchange ground states in **4-Yb** and **4-Tm**, with exchange states split by ∼0.32 cm^–1^ for **4-Yb**. This work has shown that SMM behavior is not guaranteed, even when approaching the limit of maximizing CF splitting in a Yb^3+^ complex. We have highlighted other factors such as the experimentally validated transverse *g*-values, which must also be controlled in the pursuit of Yb^3+^ zero-field SMMs.

## Experimental Section

### General Methods

All manipulations were carried out under argon with the exclusion of water and oxygen using standard Schlenk line and glovebox techniques. Toluene, hexane, and benzene were dried by refluxing over potassium, degassed, and stored over potassium mirrors. Dichloromethane (DCM) and fluorobenzene (FB) were dried over CaH_2_, degassed, and stored over 4 Å molecular sieves. C_6_D_6_ and CD_2_Cl_2_ were dried by refluxing over potassium or stirring over CaH_2_, respectively, before being vacuum distilled and freeze–pump–thaw degassed three times prior to use. **1-Ln**,^9,10^**2-Sm**,^10^ and **3-Ln**(37) were prepared by the literature methods; TEMPO^•^ was purchased from Sigma-Aldrich and purified by sublimation prior to use. ^1^H (400 and 500 MHz), ^13^C{^1^H} (100 and 126 MHz), ^11^B (128 and 160 MHz), ^19^F (376 and 470 MHz), and ^29^Si (79.5 and 99 MHz) DEPT NMR spectra were obtained on Avance III 400 or 500 MHz spectrometers at 298 K. These were referenced to the residual solvent resonance where present, or otherwise to external SiMe_4_ (^1^H, ^13^C, ^29^Si), H_3_BO_3_/D_2_O (^11^B), or trifluorotoluene/CDCl_3_ (^19^F). UV–vis-NIR spectroscopy was performed on samples in Young’s tap-appended 10 mm path-length quartz cuvettes on an Agilent Technologies Cary Series UV–vis-NIR spectrophotometer in the range 175–3300 nm. Fourier Transform infrared (FTIR) spectra were recorded as microcrystalline powders using a Bruker Tensor 27 spectrometer (ATR) or as Nujol mulls in KBr disks on a PerkinElmer Spectrum RX1 spectrometer located within a nitrogen-filled glovebox. The FTIR spectra were normalized to 0.4 absorbance for the peak at ∼1460 cm^–1^ (100–39.8% transmittance). Elemental analysis samples were prepared in an argon-filled glovebox and analyses were carried out by Martin Jennings and Anne Davies at the Microanalytical service, School of Chemistry, The University of Manchester.

#### [Tm^III^{N(Si^i^Pr_3_)_2_}_2_(TEMPO^–^)] (**2-Tm**)

A precooled (−78 °C) solution of TEMPO^•^ (0.039 g, 0.25 mmol) in toluene (5 mL) was added to a stirring, precooled (−78 °C), suspension of **1-Tm** (0.207 g, 0.25 mmol) in toluene (5 mL). The reaction mixture was allowed to warm to room temperature, becoming a yellow solution over time. After 16 h, the volatiles were removed *in vacuo* and the product redissolved in hexane (2 mL). Storage at −35 °C gave **2-Tm** as yellow crystals (0.136 g, 55%). Anal. calcd for C_45_H_102_N_3_OSi_4_Tm: C, 55.01; H, 10.46; N, 4.28. Found: C, 54.00; H, 10.69; N, 4.06. χ*T* product = 5.83 cm^3^ mol^–1^ K (Evans method). ^1^H NMR (C_6_D_6_, 400 MHz): δ −16.04 (br, ν_1/2_ = 100 Hz), −4.84 (br, ν_1/2_ = 100 Hz), −0.39 (br, ν_1/2_ = 50 Hz), 0.21 (br, ν_1/2_ = 50 Hz), 0.88 (br, ν_1/2_ = 50 Hz), 4.59 (br, ν_1/2_ = 100 Hz), 9.16 (br, ν_1/2_ = 50 Hz), 21.37 (br, ν_1/2_ = 30 Hz), 24.03 (br, ν_1/2_ = 30 Hz). The paramagnetism of **2-Tm** precluded the assignment of its ^1^H NMR spectrum, and no signals were observed in the ^13^C{^1^H} or ^29^Si DEPT NMR spectra that could be assigned to **2-Tm**. FTIR (ATR, microcrystalline): ṽ = 2934 (br), 2865 (m), 1464 (s), 1384 (s), 1358 (s), 1242 (s), 1208 (s), 1179 (s), 1131 (s), 1064 (s), 1011 (m), 993 (m), 965 (m), 946 (m), 913 (s), 877 (s), 792 (w), 727 (m), 697 (s), 657 (m), 627 (s), 542 (s), 508 (s), 469 (s), 415 (s) cm^–1^.

#### [Yb^III^{N(Si^i^Pr_3_)_2_}_2_(TEMPO^–^)] (**2-Yb**)

A precooled (−78 °C) solution of TEMPO^•^ (0.039 g, 0.25 mmol) in toluene (5 mL) was added to a stirring, precooled (−78 °C), deep-red solution of **1-Yb** (0.212 g, 0.25 mmol) in toluene (5 mL). The reaction mixture immediately turned dark green and was allowed to warm to room temperature. After 16 h, the volatiles were removed *in vacuo*, and the product was redissolved in hexane (2 mL). Storage at −35 °C gave **2-Yb** as dark green crystals (0.127 g, 52%). Anal. calcd for C_45_H_102_N_3_OSi_4_Yb: C, 54.78; H, 10.42; N, 4.26. Found: C, 54.36; H, 10.74; N, 4.03. χ*T* product = 1.40 cm^3^ mol^–1^ K (Evans method). ^1^H NMR (C_6_D_6_, 400 MHz): δ −50.02 (br, ν_1/2_ = 750 Hz), −32.23 (br, ν_1/2_ = 200 Hz), −28.87 (br, ν_1/2_ = 130 Hz), −19.89 (br, ν_1/2_ = 200 Hz), −10.54 (br, ν_1/2_ = 300 Hz). The paramagnetism of **2-Yb** precluded the assignment of its ^1^H NMR spectrum, and no signals were observed in the ^13^C{^1^H} or ^29^Si DEPT NMR spectra that could be assigned to **2-Yb**. FTIR (ATR, microcrystalline): ṽ = 2962 (s), 2866 (s), 1463 (m), 1412 (m), 1258 (s), 1061 (s), 1011 (s), 945 (s), 910 (s), 875 (s), 791 (s), 728 (s), 697 (s), 659 (s), 627 (s), 472 (s) cm^–1^.

#### [Sm^III^{N(Si^i^Pr_3_)_2_}_2_(TEMPO^•^)][B(C_6_F_5_)_4_] (**4-Sm**)

TEMPO^•^ (0.078 g, 0.5 mmol) in benzene (10 mL) was added dropwise to a solution of **3-Sm** (0.743 g, 0.5 mmol) in benzene (10 mL). The reaction mixture immediately turned red and was stirred for 16 h at room temperature. The volatiles were removed *in vacuo*, and the product was extracted with DCM (3 mL). Storage at 5 °C gave **4-Sm** as red crystals (0.152 g, 18%). Anal. calcd for C_69_H_102_BF_20_N_3_OSi_4_Sm: C, 50.44; H, 6.26; N, 2.56. Found: C, 48.78; H, 6.11; N, 2.25. χ*T* product was 0.59 cm^3^ mol^–1^ K (Evans method). ^1^H NMR (CD_2_Cl_2_, 400 MHz): δ −4.98 (br, ν_1/2_ = 260 Hz, 12 H, C*H*(CH_3_)_2_), −2.28 (br, ν_1/2_ = 31 Hz, 2 H, *p*-C*H*_2_), −0.42 (s, 12 H, C_5_H_6_N*Me*_4_), 0.54 (br, ν_1/2_ = 40 Hz, 72 H, CH(C*H*_3_)_2_), 1.92 (m, 4 H, *m*-C*H*_2_). ^11^B NMR (CD_2_Cl_2_, 128 MHz): δ −16.76. ^19^F NMR (CD_2_Cl_2_, 376 MHz): δ −132.80 (Ar–*F*). No signals were observed in the ^13^C{^1^H} or ^29^Si DEPT NMR spectra of **4-Sm** that could be assigned to the complex due to paramagnetic broadening. FTIR (Nujol mull): ṽ = 1641 (m), 1512 (m), 1413 (s), 1395 (m), 1229 (m), 1085 (m), 1063 (s), 1014 (s), 981 (s), 945 (s), 920 (s), 908 (s), 881 (s), 775 (s), 769 (s), 756 (s), 698 (s), 684 (s), 661 (s), 635 (s), 610 (s), 572 (s) cm^–1^.

#### [Tm^III^{N(Si^i^Pr_3_)_2_}_2_(TEMPO^•^)][B(C_6_F_5_)_4_] (**4-Tm**)

TEMPO^•^ (0.039 g, 0.5 mmol) in benzene (5 mL) was added dropwise to a solution of **3-Tm** (0.376 g, 0.5 mmol) in benzene (5 mL). The reaction mixture immediately turned dark red and was stirred for 16 h at room temperature. The volatiles were removed *in vacuo* and the product was extracted with DCM (1 mL). Storage at −35 °C gave **4-Tm** as red crystals (0.275 g, 66%). Anal. calcd for C_69_H_102_BF_20_N_3_OSi_4_Tm: C, 49.88; H, 6.19; N, 2.53. Found: C, 48.63; H, 6.12; N, 2.32. χ*T* product = 6.04 cm^3^ mol^–1^ K (Evans method). ^1^H NMR (CD_2_Cl_2_, 400 MHz): δ −39.16 (br, ν_1/2_ = 100 Hz, 12 H, C*H*(CH_3_)_2_), 25.59 (br, ν_1/2_ = 801 Hz, 72 H, CH(C*H*_3_)_2_); TEMPO^•^ signals not observed. ^11^B{^1^H} NMR (CD_2_Cl_2_, 128 MHz): δ −13.25. ^19^F NMR (CD_2_Cl_2_, 376 MHz): δ −128.78 (Ar–*F*). No signals were observed in the ^13^C{^1^H} or ^29^Si DEPT NMR spectra of **4-Tm** that could be assigned to the complex due to paramagnetic broadening. FTIR (ATR, microcrystalline): ṽ = 2945 (w), 2868 (w), 1642 (m), 1509 (m), 1456 (m), 1383 (w), 1273 (w), 1085 (m), 979 (m), 870 (m), 755 (w), 701 (m), 661 (m), 630 (w), 524 (w), 465 (w), 416 (w) cm^–1^.

#### [Yb^III^{N(Si^i^Pr_3_)_2_}_2_(TEMPO^•^)][B(C_6_F_5_)_4_] (**4-Yb**)

TEMPO^•^ (0.078 g, 0.5 mmol) in benzene (10 mL) was added dropwise to a solution of **3-Yb** (0.797 g, 0.5 mmol) in benzene (10 mL). The reaction mixture immediately turned dark red and was stirred for 16 h at room temperature. The volatiles were removed *in vacuo* and the product was extracted with DCM (1 mL). Storage at 5 °C gave **4-Yb** as purple crystals (0.076 g, 9%). Anal. calcd for C_69_H_102_BF_20_N_3_OSi_4_Yb: C, 49.75; H, 6.17; N, 2.52. Found: C, 48.78; H, 6.02; N, 2.31. χ*T* product = 3.14 cm^3^ mol^–1^ K (Evans method). ^11^B NMR (CD_2_Cl_2_, 128 MHz): δ −15.17. ^19^F NMR (CD_2_Cl_2_, 376 MHz): δ −130.95 (Ar–*F*). No signals were observed in the ^13^C{^1^H} or ^29^Si DEPT NMR spectra of **4-Yb** that could be assigned to the complex due to paramagnetic broadening, and we could not fully interpret the ^1^H NMR spectrum of this complex, though a broad signal at δ_H_: 11.03 ppm (br, ν_1/2_ = 430 Hz) is tentatively assigned to CH(C*H*_3_)_2_, based on this environment being the most abundant. FTIR (ATR, microcrystalline): ṽ = 2959 (w), 2863 (w), 1643 (m), 1513 (m), 1454 (w), 1386 (m), 1270 (w), 1085 (w), 974 (w), 940 (w), 881 (m), 772 (m), 681 (m), 634 (m), 573 (w), 469 (w), 411 (w) cm^–1^.

### Crystallography

The crystal data for complexes **2-Ln** (Ln = Tm and Yb) and **4-Ln** (Ln = Sm, Tm, and Yb) are compiled in Tables S1 and S2. Crystals of **2-Tm**, **2-Yb**, **4-Sm**, and **4-Yb** were examined using a Rigaku XtalLAB AFC11 diffractometer with a CCD area detector and graphite-monochromated Cu Kα (λ = 1.54178 Å) or Mo Kα radiation (λ = 0.71073 Å). Crystals of **4-Tm** were examined using an Oxford Diffraction Supernova diffractometer with a CCD area detector and mirror-monochromated Mo Kα radiation (λ = 0.71073 Å). Intensities were integrated from data recorded on 0.5° frames by ω rotation. Cell parameters were refined from the observed positions of all strong reflections in each data set. A Gaussian grid face-indexed (**2-Tm**, **2-Yb**, **4-Sm**, **4-Tm**) or multiscan (**4-Yb**) absorption correction with a beam profile was applied.^62^ The structures were solved variously using SHELXT (**2-Tm**, **2-Yb**, **4-Sm**, **4-Tm**)^63,64^ or by direct and heavy atom methods using SHELXS (**4-Yb**).^65^ The data sets were refined by full-matrix least-squares on all unique *F*^2^ values, with anisotropic displacement parameters for all non-hydrogen atoms, and with constrained riding hydrogen geometries; *U*_iso_(H) was set at 1.2 (1.5 for methyl groups) times *U*_eq_ of the parent atom. Significantly disordered solvent was observed in most structures (**2-Tm**, **2-Sm**, **4-Tm**, **4-Yb**), which could not be modeled sensibly, and in some cases the solvent identity was unclear. Contributions from disordered solvents were removed using SQUEEZE in Platon^66^ (**2-Tm**, **4-Sm**) or OLEX2^67^ (**4-Tm**, **4-Yb**). This corresponded to one molecule of hexane per complex of **2-Tm**, one molecule of DCM per complex of **4-Sm** and half a molecule of DCM or benzene per complex of **4-Tm** or **4-Yb**. The largest features in final difference syntheses were close to those of heavy atoms and there were of no chemical significance. CrysAlisPro^62^ was used for control and integration, and SHELX^63−65^ was employed through OLEX2^67^ for structure solution and refinement. ORTEP-3^68^ and POV-Ray^69^ were employed for molecular graphics.

### EPR Spectroscopy

Continuous-wave (CW) X-band (9.38 GHz) EPR spectra were collected on a Bruker EMX Plus EPR spectrometer with 1.8 T electromagnet and Stinger closed-cycle helium gas cryosystem or liquid helium cryosystem. Spectra were recorded at 6–9 K. Crystalline samples were finely ground, transferred to quartz tubes in a glovebox, and then sealed under vacuum prior to measurement. Spectra of **2-Sm**, **2-Tm**, **4-Sm**, and **4-Tm** were EPR silent except for a weak radical impurity in Tm samples. Powder spectra were collected at two sample rotations, at approximately 90° to one another to confirm lack of polycrystallinity effects (Figures S27 and S28). Spectra for **2-Yb** were collected at 20 dB attenuation with 0.4 mT modulation amplitude and spectra for **4-Yb** were collected at 15 dB attenuation with 0.2 mT modulation amplitude. The field was corrected by using a strong pitch sample (*g* = 2.0028). Simulations for the **2-Yb** spectrum were performed using the pepper function in *EasySpin* 6.0.2.^43^ Anisotropic Yb *g*-strains were employed to account for all anisotropic line broadening effects. Further simulation details are provided in SI.

### Magnetic Measurements

Magnetic measurements were performed using a Quantum Design MPMS-XL7 superconducting quantum interference device (SQUID) magnetometer (**2-Sm** or **4-Sm**) or an MPMS3 SQUID magnetometer (**2-Tm**, **4-Tm**, **2-Yb**, and **4-Yb**). Crystalline samples with mass ranging between 15 and 40 mg were crushed with a mortar and pestle under an inert atmosphere, and then loaded into a borosilicate glass NMR tube along with *ca*. 20–35 mg powdered eicosane, which was then evacuated and flame-sealed to a length of *ca*. 5 cm. The eicosane was melted by gently heating the tube with a low-power heat gun in order to immobilize the crystallites. The NMR tube was then mounted in the center of a drinking straw by using friction by wrapping it with Kapton tape, and the straw was then fixed to the end of the sample rod. The raw data were corrected for the diamagnetic contribution of the sample holder and eicosane by using calibrated blanks. Data collected on the MPMS3 SQUID were divided by the following correction factors to account for the shape of the sample, as calculated using the Quantum Design MPMS3 Geometry Simulator: 1.037 (**2-Tm**), 1.017 (**4-Tm**), 0.985 (**2-Yb**), 0.997 (**4-Yb**). Finally, the data were corrected for the intrinsic diamagnetic contribution of the sample, estimated as the molecular weight (g/mol) multiplied by 0.5 × 10^–6^ cm^3^ K mol^–1^. The cocrystallized solvent was assumed to be lost on drying and was not included in the molecular weight.

For the weakly magnetic Sm compounds, dc susceptibility measurements were collected on warming in 1 T (**2-Sm**) or 0.5 T (**4-Sm**) dc fields. For Tm and Yb compounds, dc susceptibilities were measured on cooling in a 0.1 T dc field. The magnetic moment of **4-Yb** was slow to equilibrate in field. To measure comparable equilibrium traces for **2-Yb** and **4-Yb**, both were measured with long wait times at low temperatures. Sweep rates between temperatures were 5 K/min for 300–100 K, 2 K/min for 100–10 K, and 1 K/min for 10–1.8 K. The susceptibility between 50 and 1.8 K was measured in 21 log-spaced steps with 10 min waits at 50–30 K, 30 min waits at 30–20 K, and 45 min waits at 20–1.8 K. Zero-field-cooled (ZFC) and field-cooled (FC) susceptibilities were also measured for **2-Yb** and **4-Yb**; the sample was prepared by cooling in zero field from 150 K. The ZFC/FC susceptibilities were measured with a 0.9 K/min constant sweep rate, recording points every 2 K between 2 and 100 K, measured on warming (ZFC) or cooling (FC).

Magnetization measurements were performed between 0 and 7 T at 2, 4, and (for Yb samples) 10 K. For Sm samples, points were recorded at 0.25 T steps between 0 and 1 T, 0.5 T steps between 1 and 2, and 1 T steps between 2 and 7 T. For Tm and Yb samples, points were recorded at 0.2 T steps between 0 and 1 and 0.5 T steps between 1 and 7 T, with 10 min waits at each field point. Hysteresis curves were recorded for **2-Yb** and **4-Yb** at 2 K and between ±7 T. The sweep rates were as follows: 22.0 Oe/s for 0 < |H| < 1 T, 51.9 Oe/s for 1 < |H| < 2 T and 90.6 Oe/s for 2 < |H| < 7 T.

### *Ab Initio* Calculations

CASSCF-SO and XMS-CASPT2 calculations were performed in OpenMolcas using version 18.09 (tag: 790-g23d48b3-dirty) for Tm and Sm complexes, and version 20.10 (tag: 268-g4ac74574-dirty) for Yb complexes.^46,47^ The single crystal XRD coordinates were used with no optimization, in the case of disorder, the major component was selected.^10^ For **2-Ln**, the complete molecule in the asymmetric unit with no local symmetry was selected. For **4-Ln**, calculations were performed on the cationic [Ln^III^{N(Si^i^Pr_3_)_2_}_2_(TEMPO)]^+^ fragment and on the same coordinates with an artificial Ln(III)-TEMPO^–^ charge distribution (**[4-Ln]**^**0**^). Integrals were performed in SEWARD using basis sets from the ANO-RCC library^70,71^ with VTZP quality for Ln atoms, VDZP quality for coordinating N atoms and the N and O atoms of TEMPO, and VDZ quality for all other atoms, employing the second order DKH transformation. Cholesky decomposition of the two-electron integrals with a threshold of 10^–8^ was performed to save disk space and reduce the computational demand.

For complexes **2-Ln** and **[4-Ln]**^**0**^, the active space consisted of seven f orbitals. An unrestricted KS-DFT calculation of the sextet states was performed to obtain initial orbitals for **[4-Sm]**^**0**^. For the cations of **4-Ln**, the active space consisted of the seven f orbitals and one N–O π* orbital of TEMPO (Figure S45). The optimized orbitals of **[4-Ln]**^**0**^ were used as guess orbitals for the calculations on **4-Ln**, with the N–O π* antibonding orbital of TEMPO (HOMO) moved into the active space. The molecular orbitals (MOs) were optimized in state-averaged CASSCF calculations in the RASSCF module, with the number of roots shown in Table S4. For **4-Ln**, averages were performed overall for all configurations arising from the selected Ln free-ion term(s) and TEMPO^•^ radical, not including charge transfer states. The number of roots was varied for **4-Sm** and **4-Tm**, selected based on the Ln(III) free-ion terms involved, and analogous calculations were performed on **[4-Sm]**^**0**^ and **[4-Tm]**^**0**^ for comparison. Calculations with the maximum number of roots were chosen for comparison to experimental data. Orbital images were created using Pegamoid v.2.6.1.^72^

Extended multistate complete active space second-order perturbation theory (XMS-CASPT2) calculations with an IPEA shift of 0.25 were performed in CASPT2 for **2-Yb**, **[4-Yb]**^**0**^ and attempted for **4-Yb** (did not converge).^48,49^ Selected CASSCF spin-free roots (Table S4) or the mixed spin-free wave functions from XMS-CASPT2 were mixed by spin–orbital coupling in RASSI. The magnetic susceptibility and magnetization vs field was calculated in SINGLE_ANISO. For calculations on **2-Ln** and **[4-Ln]**^**0**^ the spin–orbit wave functions were decomposed into a CF Hamiltonian with the *z*-direction defined by the *g*_1_ vector of the ground state doublet or pseudodoublet.

## Data Availability

A preprint of this article was previously deposited on *ChemRxiv*.^73^ Research data files supporting this publication are available from FigShare at DOI: 10.6084/m9.figshare.26212607.

## Supporting Information Available

Additional experimental details, materials, and methods, and data associated with this manuscript (PDF)
