# Supplementary material for: Trigonal Planar Heteroleptic Lanthanide(III) Bis(silyl)amide Complexes Containing Aminoxyl Radicals and Anions
Source: Inorg Chem. 2024 Nov 12;63(47):22422–34. doi: 10.1021/acs.inorgchem.4c03281 (PMC11600508; doi:10.1021/acs.inorgchem.4c03281)
Supplement: Supplementary file 1 — ic4c03281_si_001.pdf [file ic4c03281_si_001.pdf]

Supporting Information

Trigonal planar heteroleptic lanthanide(III) bis(silyl)amide complexes containing aminoxyl radicals and anions

Gemma K. Gransbury,[a,†] Hannah M. Nicholas,[a,†] Siobhan R. Murphy,[a] Jack Emerson-King,[a] Michele Vonci,[a] Conrad A. P. Goodwin,[a] Richard E. P. Winpenny,[a] Nicholas F. Chilton,[a,b] Marcus J. Giansiracusa,[a,c,]* and David P. Mills[a,]*

[a] Department of Chemistry, The University of Manchester, Oxford Road, Manchester, M13 9PL, U.K.

[b] Research School of Chemistry, Australian National University, Building 137, Sullivans Creek Road, Canberra, ACT, 2601, Australia.

[c] School of Chemistry, The University of Melbourne, Parkville, Victoria, 3010, Australia.

[†]Contributed equally as co-first authors.

*Email: david.mills@manchester.ac.uk, marcus.giansiracusa@unimelb.edu.au.

Contents

1. Crystallography....S2

2. Molecular Structures....S4

3. NMR Spectroscopy....S7

4. ATR-IR Spectroscopy....S13

5. UV-vis-NIR Spectroscopy....S16

7. EPR Spectroscopy....S19

8. Magnetism....S22

8. CASSCF-SO Electronic Structure....S32

9. References....S51

1. Crystallography

Table S1. Crystallographic data for **2-Tm**, **2-Yb** and **4-Sm**.

	**2-Tm**	**2-Yb**	**4-Sm**
Empirical formula	$C_{45}H_{102}N_3OSi_4Tm$ + solvent	$C_{45}H_{102}N_3OSi_4Yb$	$C_{69}H_{102}BF_{20}N_3OSi_4Sm$ + solvent
Formula weight	982.58 + solvent	986.69	1643.05 + solvent
Temperature/K	150.01(10)	149.97(10)	150.00(10)
Crystal system	monoclinic	monoclinic	triclinic
Space group	*C*2/*c*	*C*2/*c*	*P*–1
a/Å	19.4385(4)	19.4212(5)	11.4419(2)
b/Å	19.7578(5)	19.7262(5)	16.2597(3)
c/Å	41.2765(10)	41.2030(10)	20.5680(5)
α/°	90	90	93.2417(16)
β/°	99.214(2)	99.151(3)	93.4383(17)
γ/°	90	90	90.2629(15)
Volume/Å^3	15648.1(6)	15584.2(7)	3813.36(13)
Z	12	12	2
ρ_{calc}g/cm^3	1.251	1.262	1.431
μ/mm^{-1}	1.826	1.926	0.924
F(000)	6312	6324	1696
Crystal size/mm^3	0.203 × 0.163 × 0.077	0.115 × 0.079 × 0.040	0.260 × 0.197 × 0.101
Radiation	MoKα (λ = 0.71073)	MoKα (λ = 0.71073)	MoKα (λ = 0.71073)
2Θ range for data collection/°	3.012 to 50.700	3.016 to 50.700	4.578 to 50.698
Index ranges	–23 ≤ h ≤ 23, –22 ≤ k ≤ 23, –49 ≤ l ≤ 48	–23 ≤ h ≤ 23, –17 ≤ k ≤ 23, –49 ≤ l ≤ 40	–13 ≤ h ≤ 13, –19 ≤ k ≤ 19, –24 ≤ l ≤ 24
Reflections collected	50804	45666	38381
Independent reflections	14227 [R_{int} = 0.0302, R_{sigma} = 0.0306]	14202 [R_{int} = 0.0446, R_{sigma} = 0.0549]	13854 [R_{int} = 0.0274, R_{sigma} = 0.0336]
Data/restraints/parameters	12754/1165/780	11427/1210/780	12365/1634/987
Goodness-of-fit on F^2	1.036	1.021	1.036
Final R indexes [I>=2σ (I)]	R_1 = 0.0229, wR_2 = 0.0521	R_1 = 0.0342, wR_2 = 0.0678	R_1 = 0.0276, wR_2 = 0.0654
Final R indexes [all data]	R_1 = 0.0274, wR_2 = 0.0535	R_1 = 0.0514, wR_2 = 0.0720	R_1 = 0.0329, wR_2 = 0.0670
Largest diff. peak/hole / e Å^{-3}	0.422/–0.361	0.946/–0.955	1.453/–0.536

Table S2. Crystallographic data for **4-Tm** and **4-Yb**.

	**4-Tm**	**4-Yb**
Empirical formula	$C_{69}H_{102}BF_{20}N_3OSi_4Tm$ + solvent	$C_{69}H_{102}BF_{20}N_3OSi_4Yb$ + solvent
Formula weight	1661.63 + solvent	1665.74 + solvent
Temperature/K	149.9(3)	150.00(10)
Crystal system	triclinic	triclinic
Space group	*P*–1	*P*–1
a/Å	11.5015(4)	11.51811(18)
b/Å	16.1656(5)	16.17425(19)
c/Å	20.6506(8)	20.6556(2)
α/°	93.294(3)	93.3602(9)
β/°	92.879(3)	92.2499(10)
γ/°	90.168(3)	90.3638(11)
Volume/Å^3	3828.3(2)	3838.36(8)
Z	2	2
ρ_{calc}g/cm^3	1.441	1.441
μ/mm^{-1}	1.313	1.372
F(000)	1710	1746
Crystal size/mm^3	0.244 × 0.154 × 0.104	0.373 × 0.272 × 0.210
Radiation	MoKα (λ = 0.71073)	MoKα (λ = 0.71073)
2Θ range for data collection/°	4.34 to 50.696	3.954 to 51.364
Index ranges	–13 ≤ h ≤ 14, –19 ≤ k ≤ 18, –24 ≤ l ≤ 25	–14 ≤ h ≤ 14, –19 ≤ k ≤ 18, –25 ≤ l ≤ 22
Reflections collected	21780	48742
Independent reflections	14305 [R_{int} = 0.0439, R_{sigma} = 0.1018]	14567 [R_{int} = 0.0317, R_{sigma} = 0.0334]
Data/restraints/parameters	11298/190/979	13239/1577/979
Goodness-of-fit on F^2	1.015	1.033
Final R indexes [I>=2σ (I)]	R_1 = 0.0542, wR_2 = 0.0925	R_1 = 0.0343, wR_2 = 0.0802
Final R indexes [all data]	R_1 = 0.0738, wR_2 = 0.1029	R_1 = 0.0391, wR_2 = 0.0821
Largest diff. peak/hole / e Å^{-3}	1.398/–1.032	2.891/–1.314

2. Molecular Structures

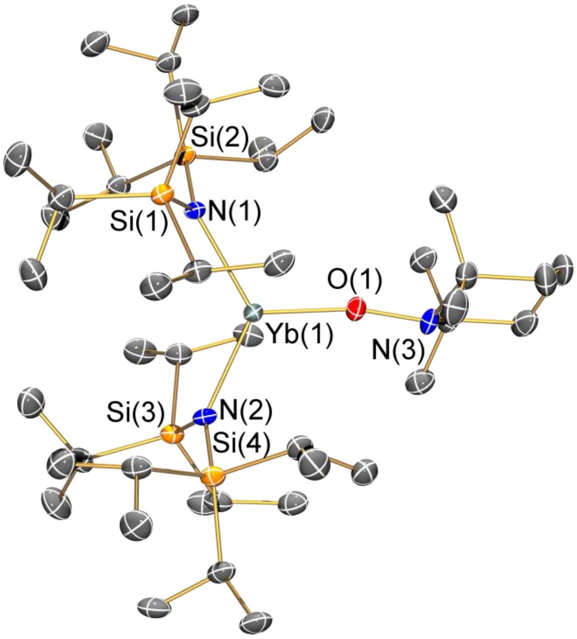

Figure S1. Molecular structure of [$Yb^{III}\{N(Si^{i}Pr_3)_2\}_2(TEMPO^{-})$] (**2-Yb**) with selected atom labeling. Displacement ellipsoids set at 50% probability level, hydrogen atoms are omitted for clarity. There are 1.5 molecules in the asymmetric unit, the molecule with no local symmetry is shown. C atoms are gray, N are blue, O are red and Si are orange. Selected distances and angles: Yb(1)–N(1), 2.285(3) Å; Yb (1)–N(2), 2.250(3) Å; Yb(1)–O(1), 2.010(2) Å; N(3)–O(1), 1.448(3) Å; N(1)–Yb(1)–N(2), 125.69(10)°; N(1)–Yb(1)–O(1), 117.72(9)°; N(2)–Yb(1)–O(1), 116.23(10)°; Yb(1)–O(1)–N(1), 167.5(2)°; Yb(1)···N(1)N(2)O(1) mean plane, 0.075(2) Å; N(3)···O(1)C(37)C(41) mean plane, 0.418(4) Å.

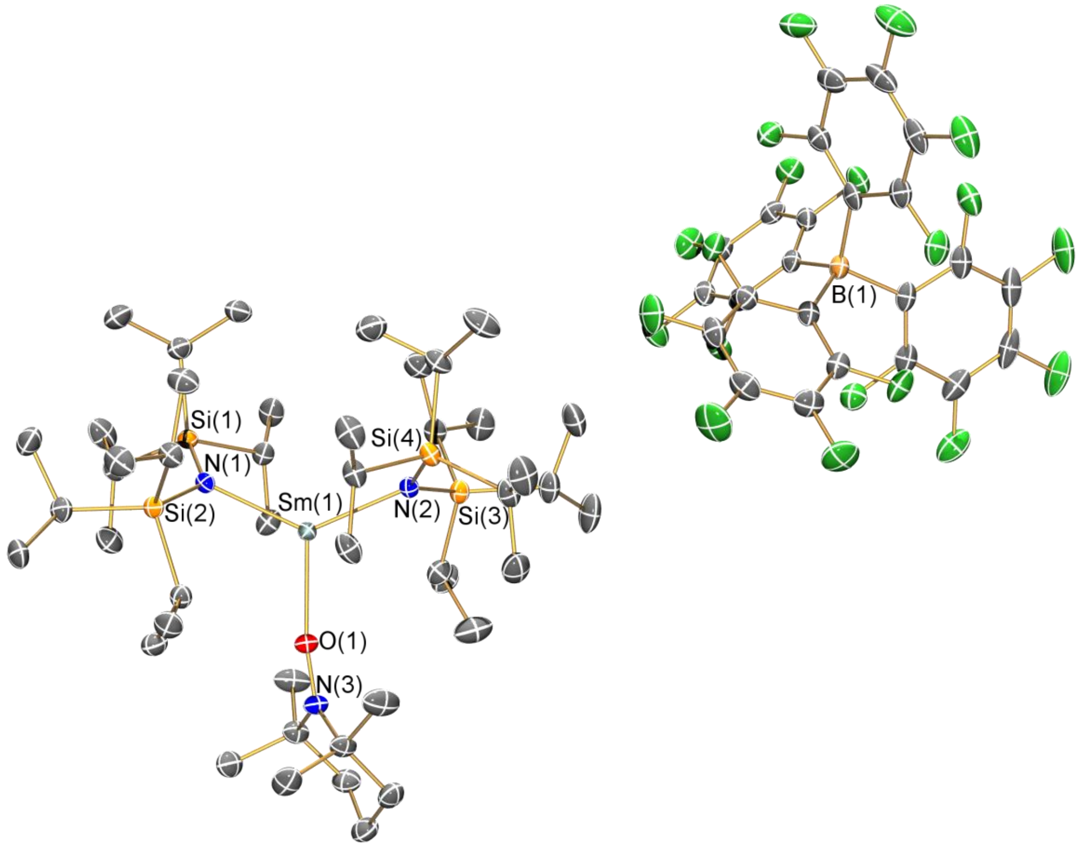

Figure S2. Molecular structure of [$Sm^{III}\{N(Si^iPr_3)_2\}_2$(TEMPO$^•$)][$B(C_6F_5)_4$] (**4-Sm**) with selected atom labeling. Displacement ellipsoids set at 50% probability level, hydrogen atoms are omitted for clarity. C atoms are gray, N are blue, O are red, Si are orange and F atoms are green. Selected distances and angles: Sm(1)–N(1), 2.318(2) Å; Sm(1)–N(2), 2.283(2) Å; Sm(1)–O(1), 2.338(2) Å; N(3)–O(1), 1.296(2) Å; N(1)–Sm(1)–N(2), 130.86(7)°; N(1)–Sm(1)–O(1), 115.39(6)°; N(2)–Sm(1)–O(1), 113.74(6)°; Sm(1)–O(1)–N(1), 170.94(13)°; Sm(1)···N(1)N(2)O(1) mean plane, 0.0120(10) Å; N(3)···O(1)C(37)C(41) mean plane, 0.028(2) Å.

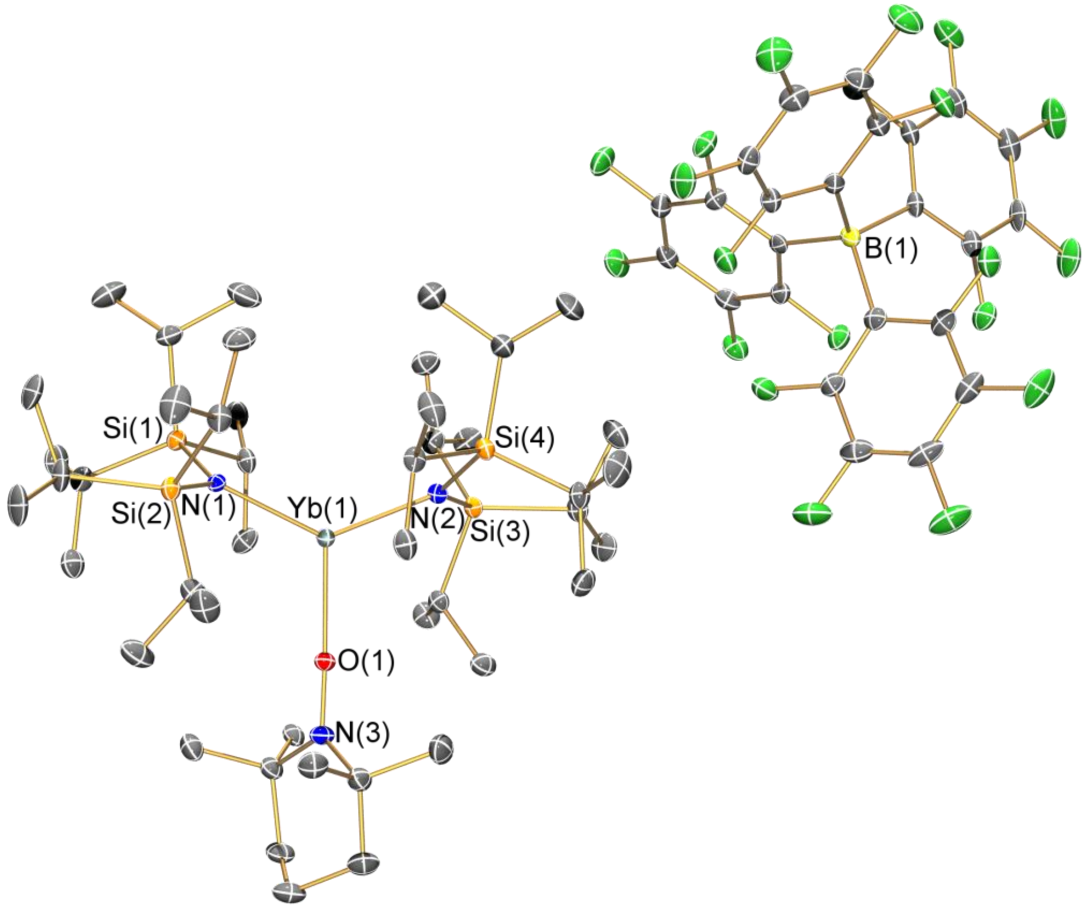

Figure S3. Molecular structure of [$Yb^{III}\{N(Si^iPr_3)_2\}_2$(TEMPO$^\bullet$)][$B(C_6F_5)_4$] (**4-Yb**) with selected atom labeling. Displacement ellipsoids set at 50% probability level, hydrogen atoms are omitted for clarity. C atoms are gray, N are blue, O are red, Si are orange and F atoms are green. Selected distances and angles: Yb(1)–N(1), 2.207(2) Å; Yb(1)–N(2), 2.214(2) Å; Yb(1)–O(1), 2.206(2) Å; N(3)–O(1), 1.305(3) Å; N(1)–Yb(1)–N(2), 134.01(9)°; N(1)–Yb(1)–O(1), 115.21(8)°; N(2)–Yb(1)–O(1), 110.77(8)°; Yb(1)–O(1)–N(1), 177.5(2)°; Yb(1)···N(1)N(2)O(1) mean plane, 0.0151(14) Å; N(3)···O(1)C(37)C(43) mean plane, 0.078(3) Å.

3. NMR Spectroscopy

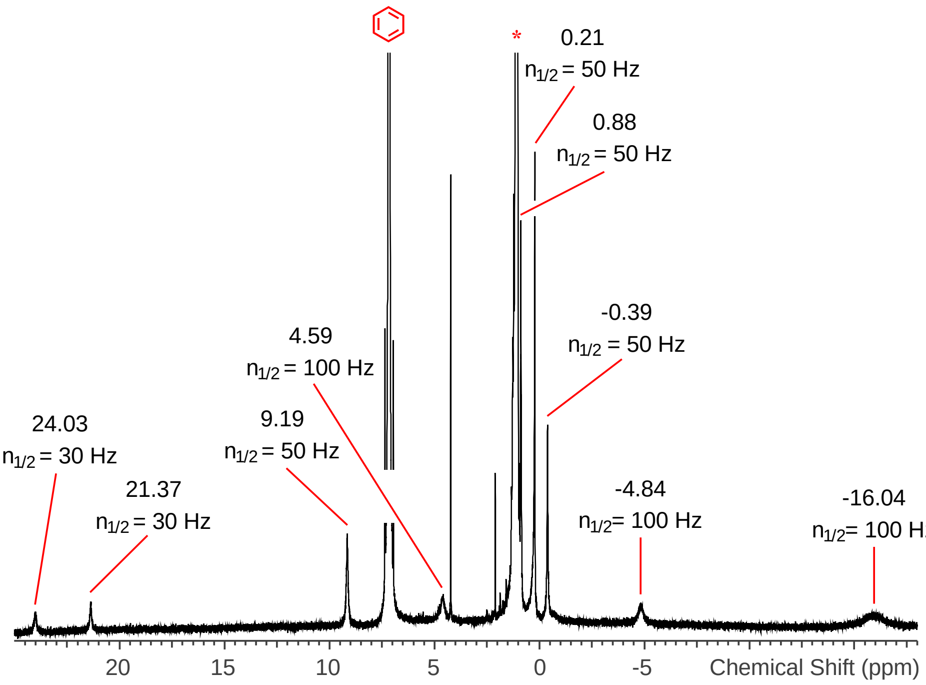

Figure S4. ^{1}H NMR spectrum (400 MHz, swept from +200 to –200 ppm) of **2-Tm** in C_6D_6, zoomed in the region +25 to –18 ppm. * denotes $HN(Si^iPr_3)_2$ impurity, solvent residual denoted.

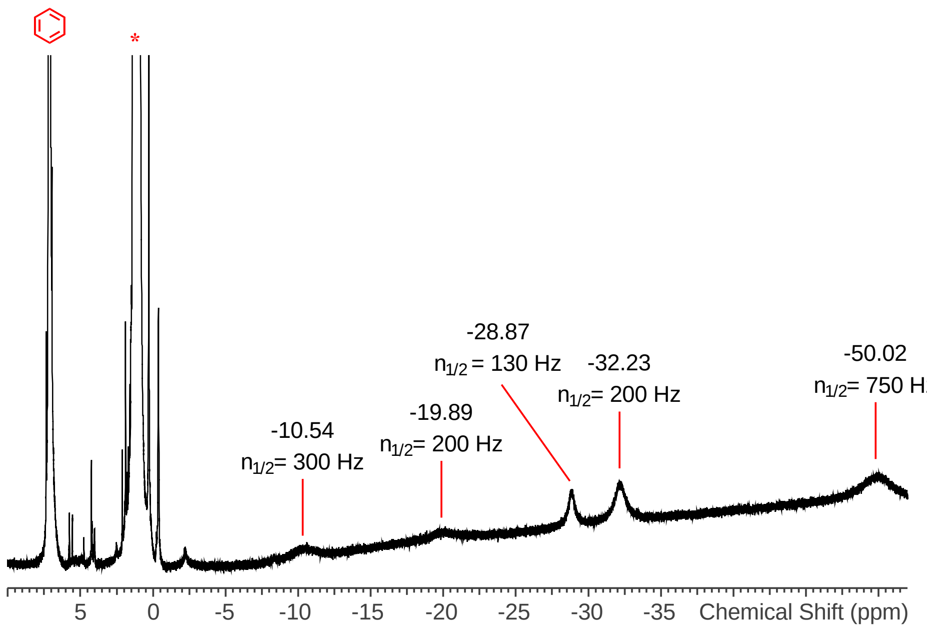

Figure S5. ^{1}H NMR spectrum (400 MHz, swept from +200 to –200 ppm) of **2-Yb** in C_6D_6, zoomed in the region +10 to –52 ppm. * denotes $HN(Si^iPr_3)_2$ impurity, solvent residual denoted.

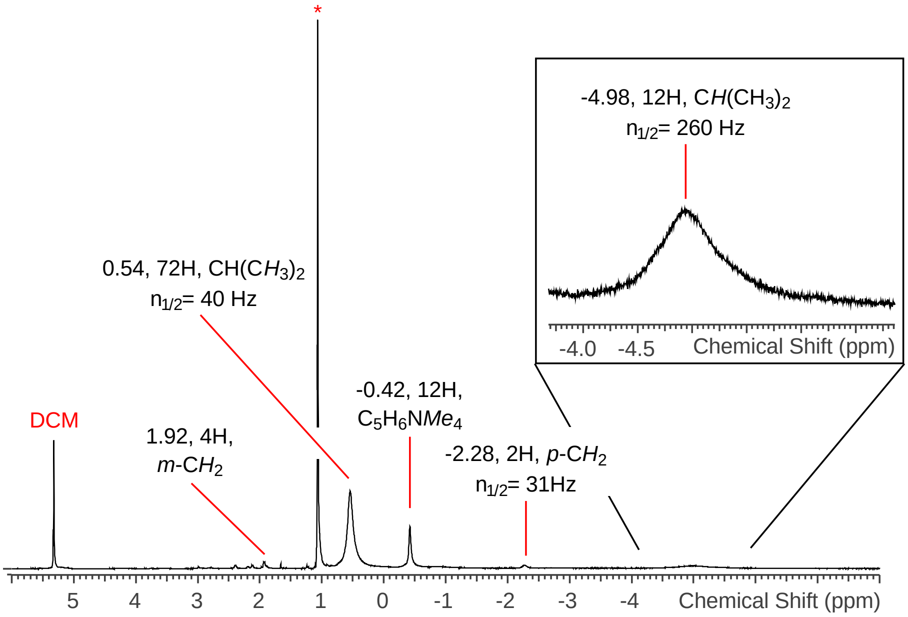

Figure S6. ^{1}H NMR spectrum (400 MHz, swept from +200 to –200 ppm) of **4-Sm** in CD_2Cl_2, zoomed in the region +6 to –8 ppm. * denotes $HN(Si^{i}Pr_3)_2$ impurity, solvent residual denoted.

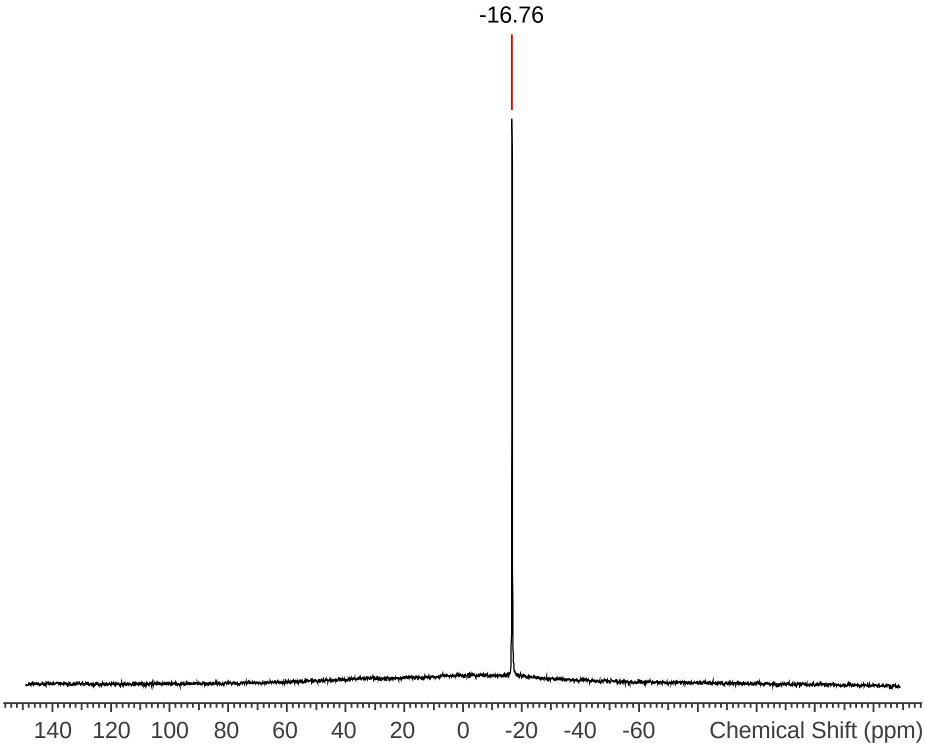

Figure S7. ^{11}B NMR spectrum (128 MHz) of **4-Sm** in CD_2Cl_2.

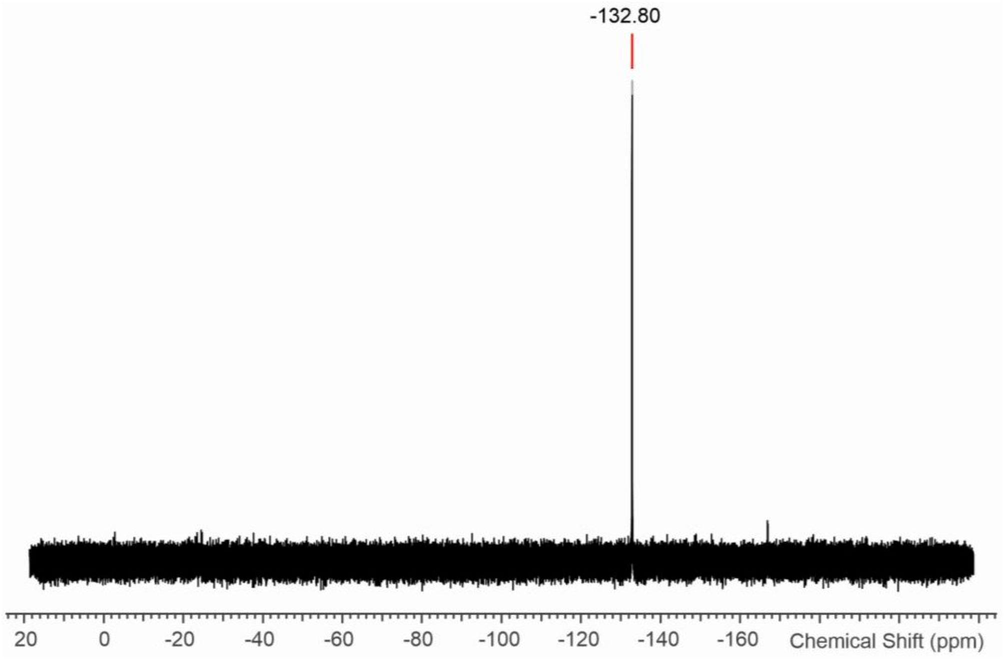

Figure S8. ^{19}F NMR spectrum (376 MHz) of **4-Sm** in CD_2Cl_2.

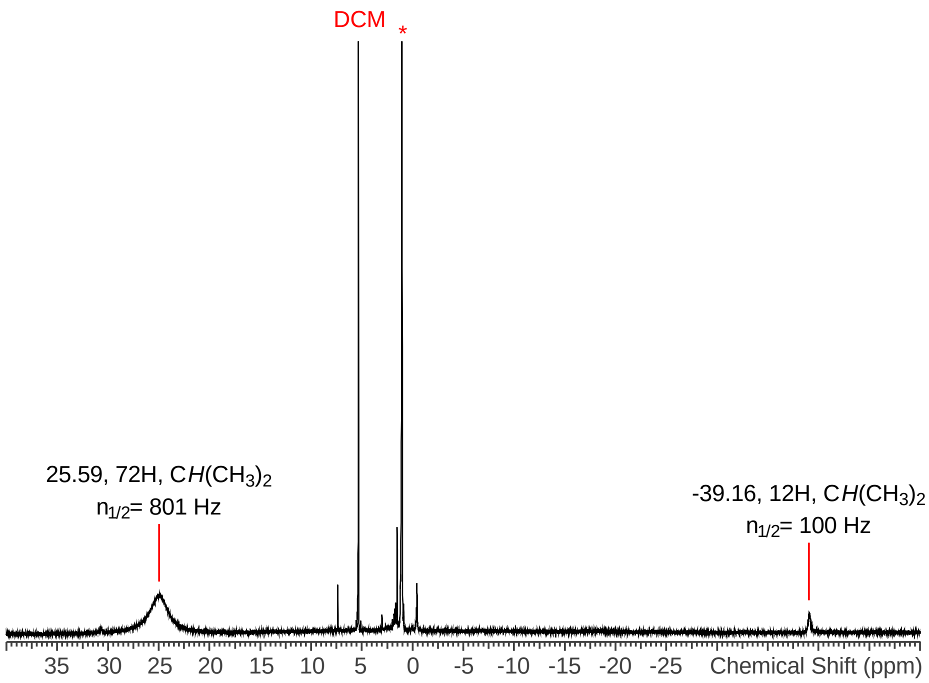

Figure S9. ^{1}H NMR spectrum (400 MHz, swept from +200 to –200 ppm) of **4-Tm** in CD_2Cl_2, zoomed in the region +40 to –50 ppm. * denotes $HN(Si^{i}Pr_3)_2$ impurity, solvent residual denoted.

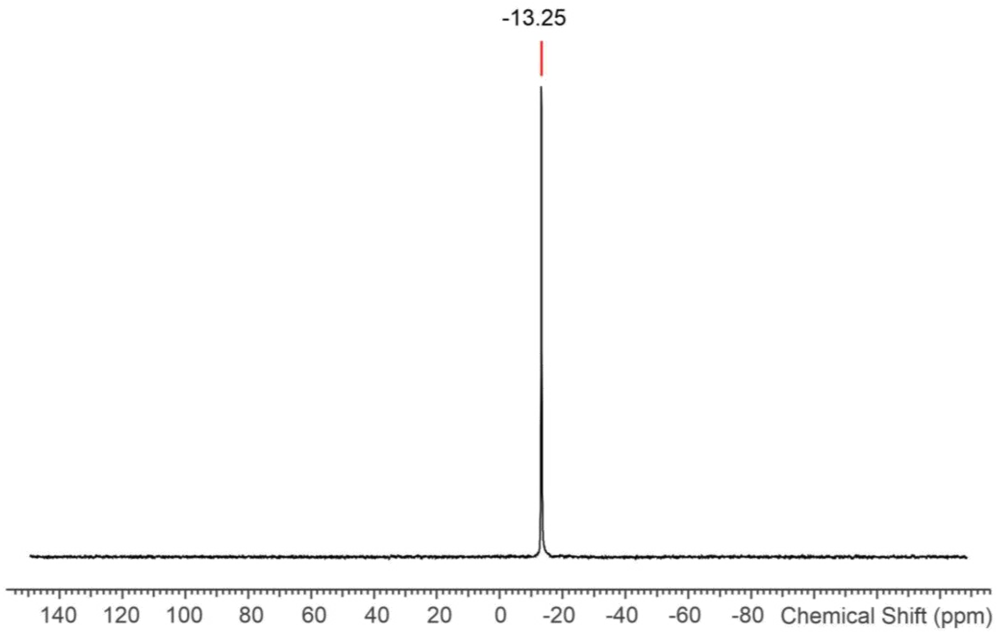

Figure S10. ^{11}B NMR spectrum (128 MHz) of **4-Tm** in CD_2Cl_2.

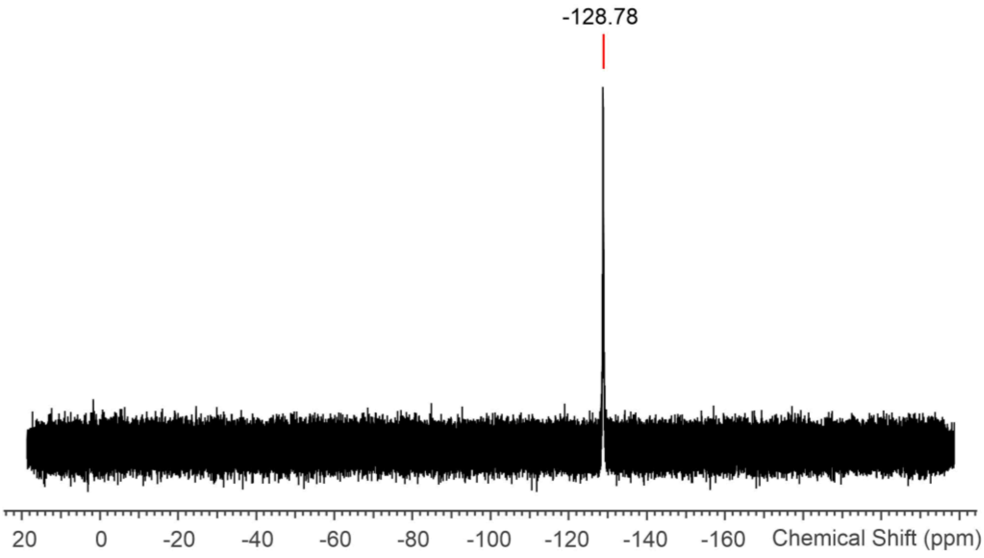

Figure S11. ^{19}F NMR spectrum (376 MHz) of **4-Tm** in CD_2Cl_2.

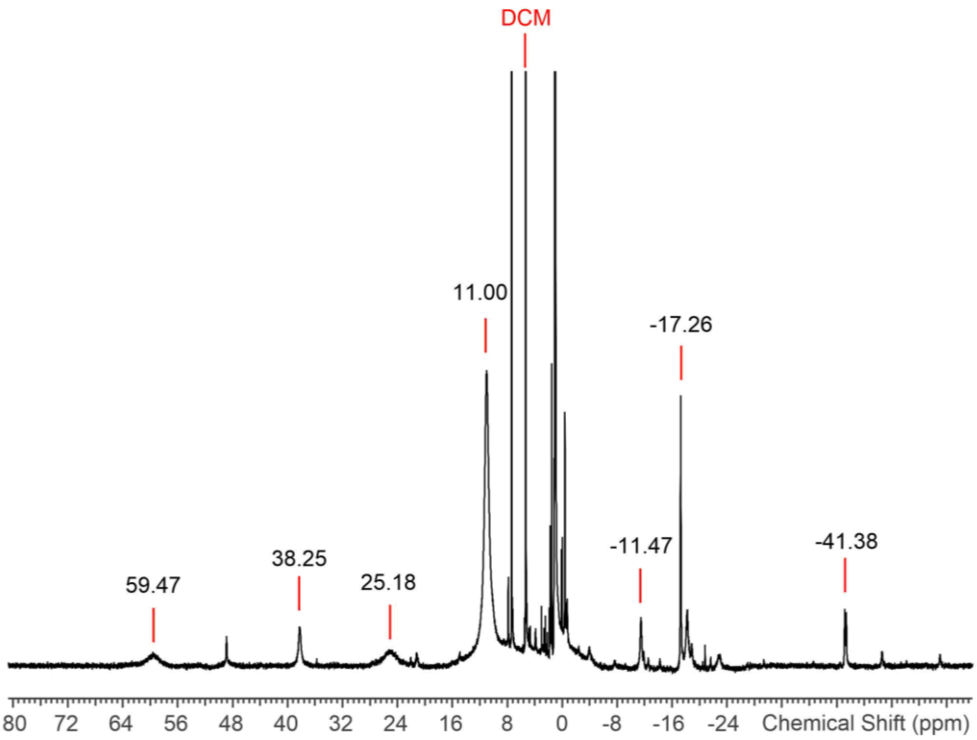

Figure S12. ^{1}H NMR spectrum (400 MHz, swept from +200 to –200 ppm) of **4-Yb** in CD_2Cl_2, zoomed in the region +80 to –60 ppm.

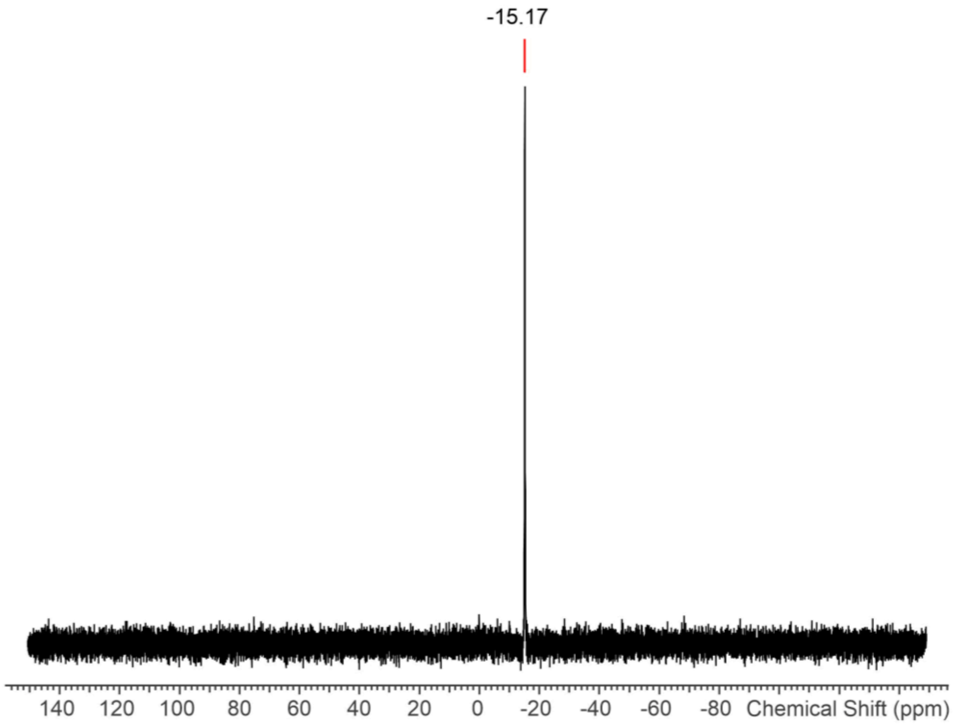

Figure S13. ^{11}B NMR spectrum (128 MHz) of **4-Yb** in CD_2Cl_2.

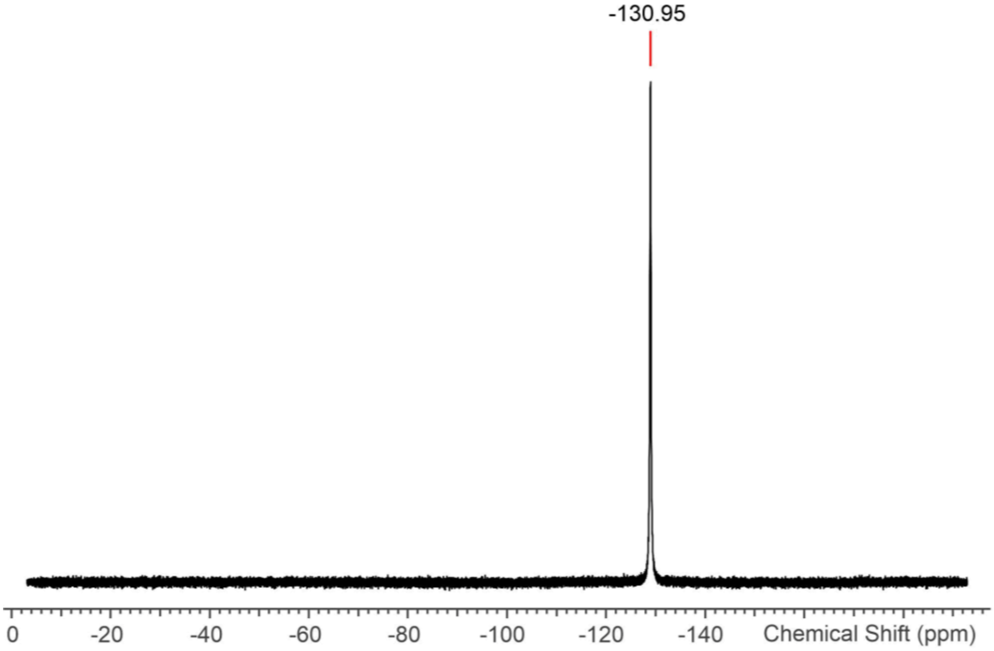

Figure S14. ^{19}F NMR spectrum (376 MHz) of **4-Yb** in CD_2Cl_2.

4. ATR-IR Spectroscopy

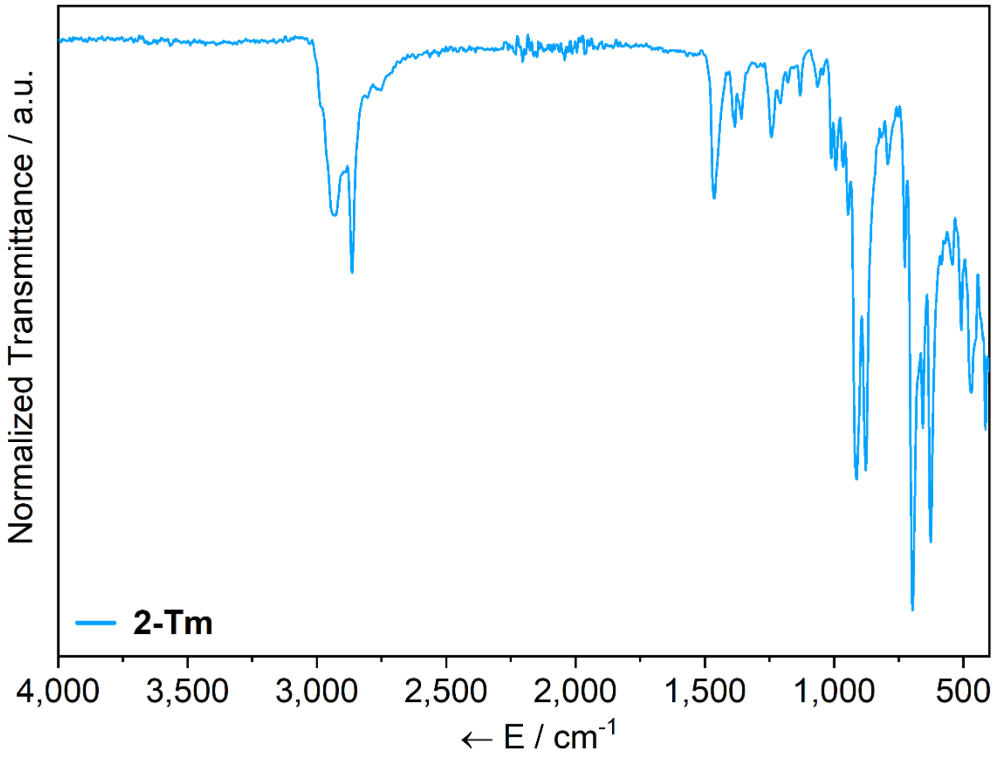

Figure S15. ATR-IR spectrum of **2-Tm** as a microcrystalline powder.

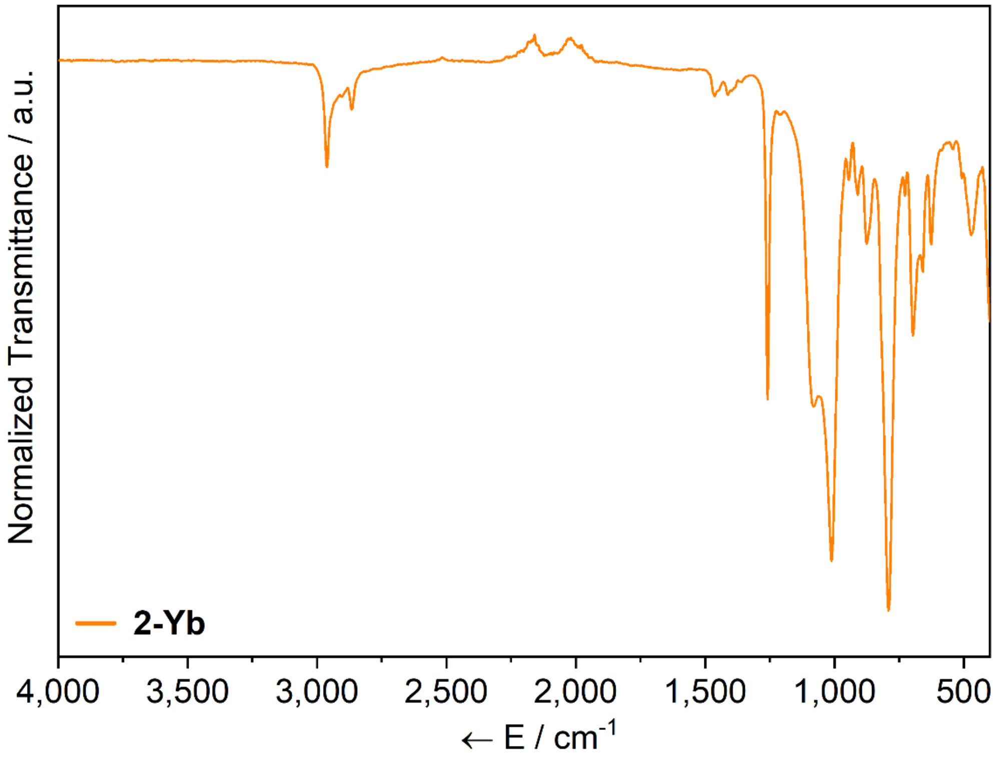

Figure S16. ATR-IR spectrum of **2-Yb** as a microcrystalline powder.

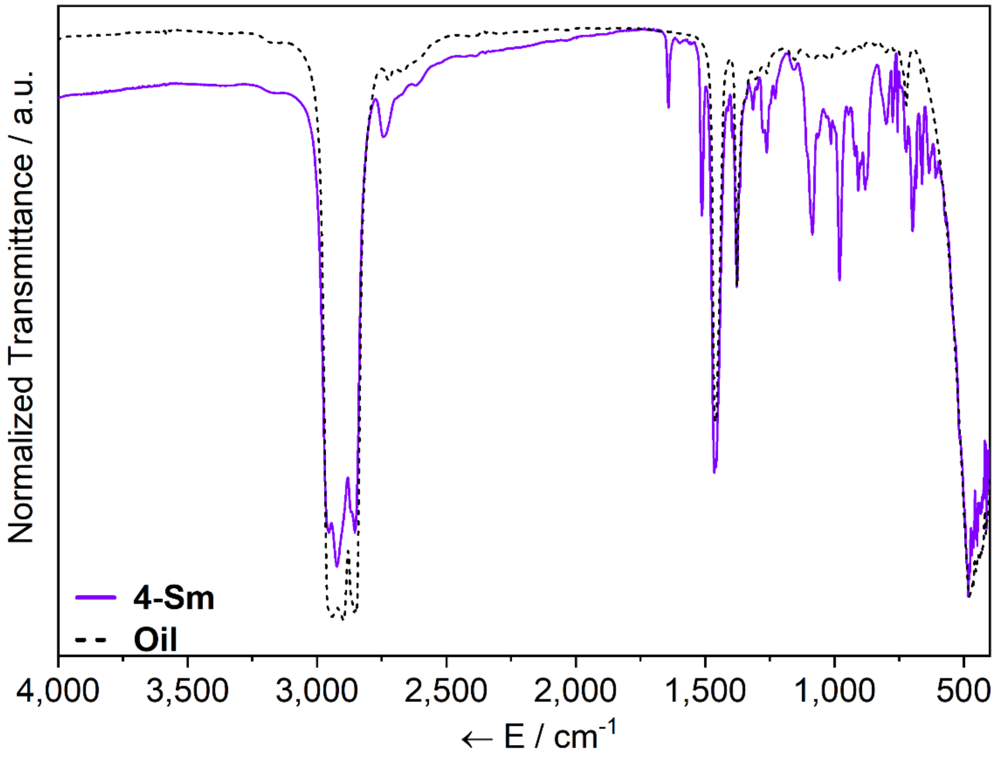

Figure S17. FTIR spectrum of **4-Sm** as a Nujol mull on KBr discs. Oil = Nujol/KBr blank.

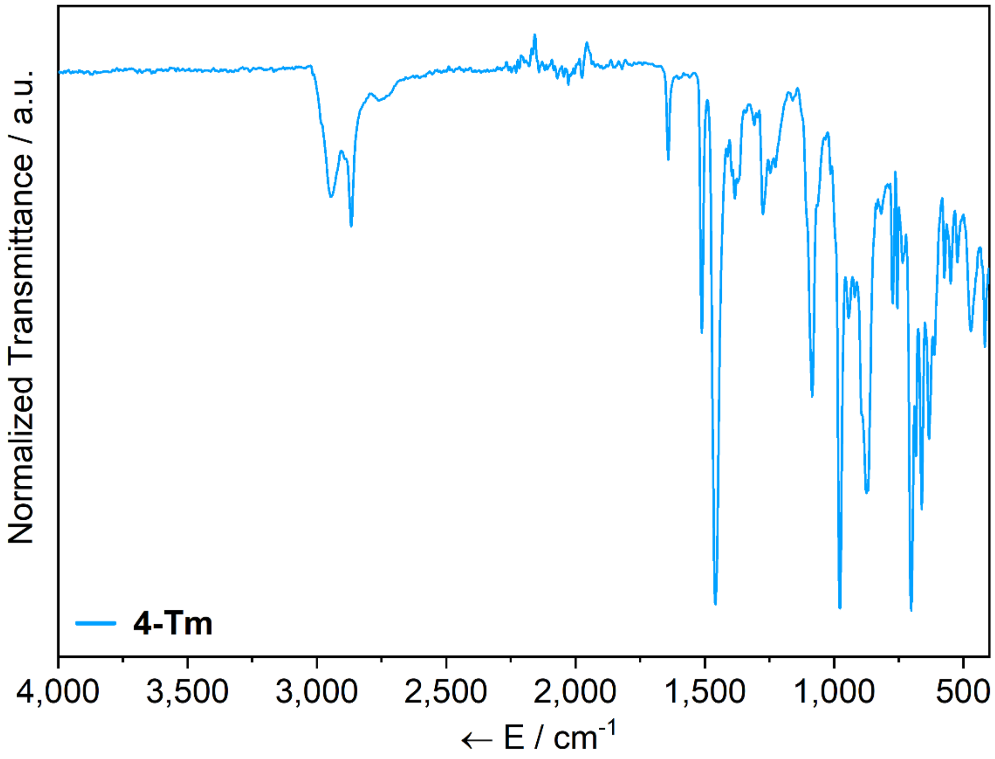

Figure S18. ATR-IR spectrum of **4-Tm** as a microcrystalline powder.

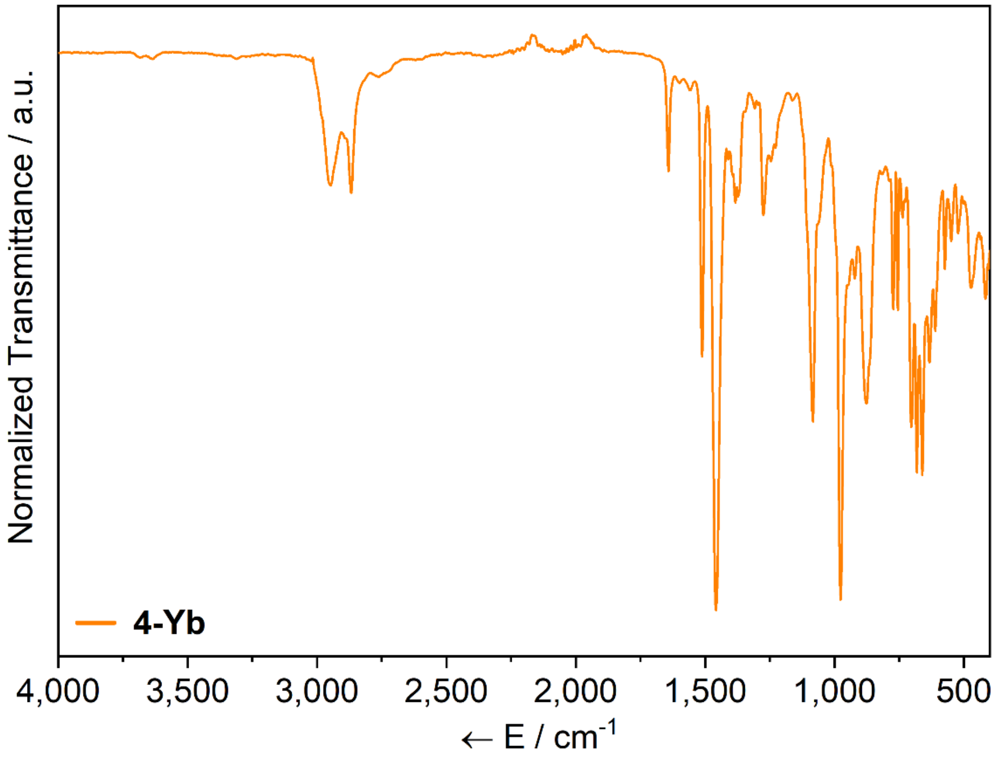

Figure S19. ATR-IR spectrum of **4-Yb** as a microcrystalline powder.

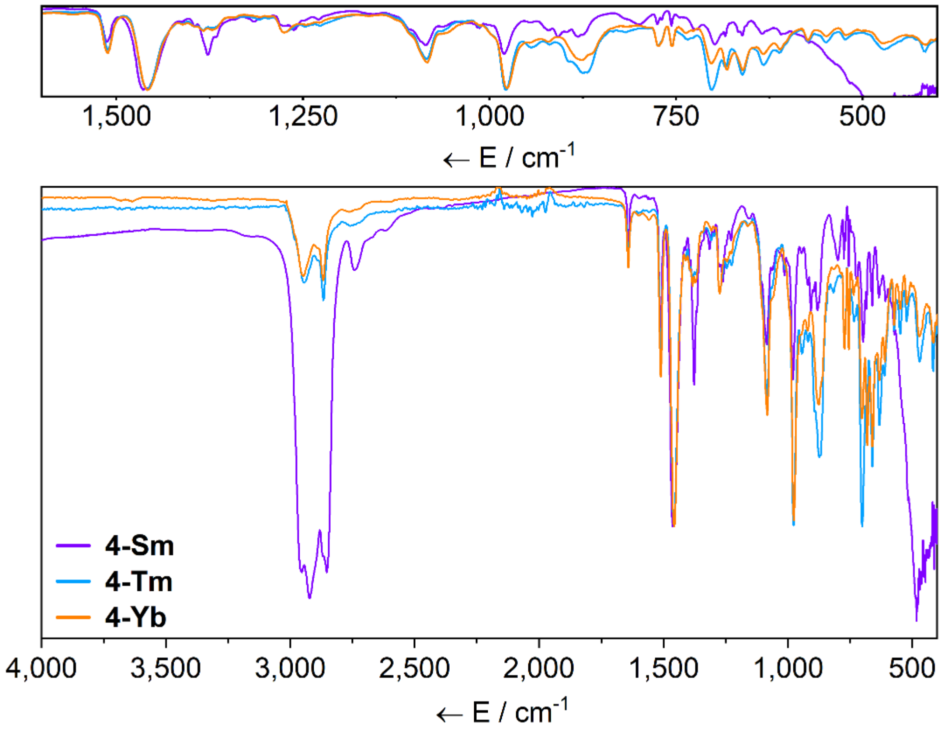

Figure S20. ATR-IR spectrum of **4-Ln** as a microcrystalline powder (**4-Tm** and **4-Yb**) or as a Nujol mull on KBr discs (**4-Sm**). Inset shows fingerprint region between 1,600–400 cm^{-1}.

5. UV-vis-NIR Spectroscopy

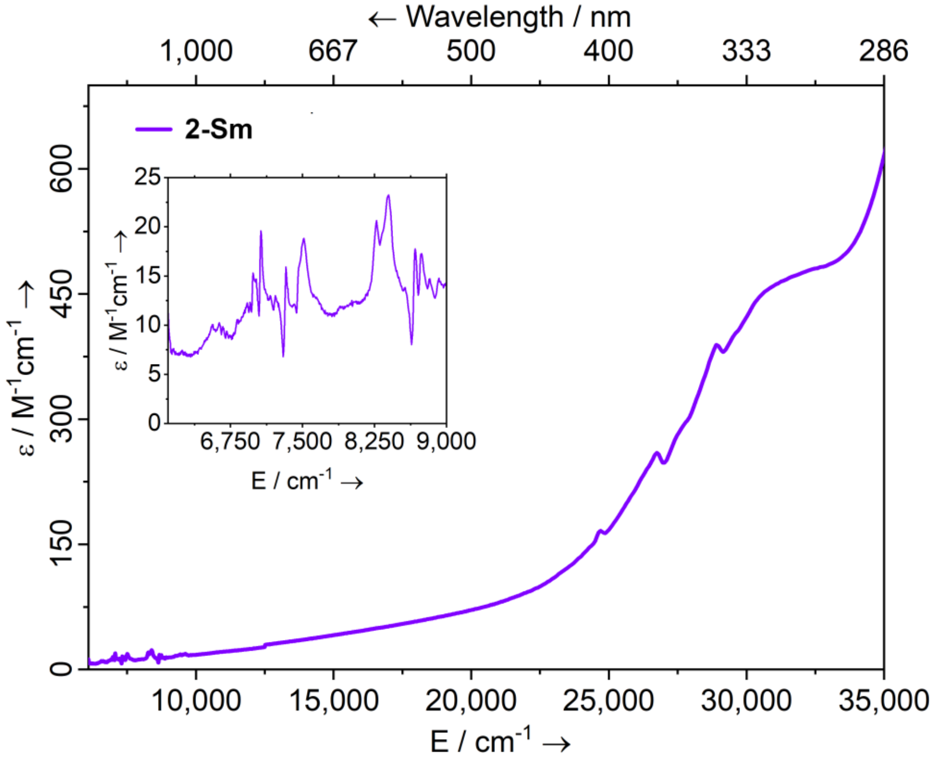

Figure S21. Room temperature UV-vis-NIR spectrum of **2-Sm** (1 mM in DCM) from 6,100–35,000 cm^{-1}.

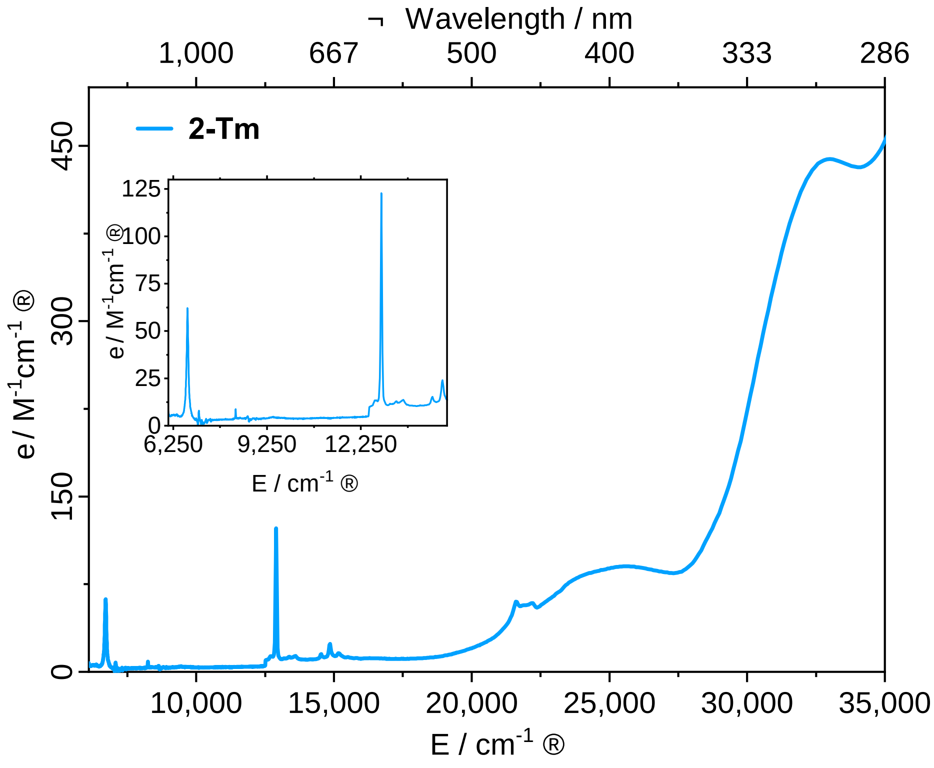

Figure S22. Room temperature UV-vis-NIR spectrum of **2-Tm** (1 mM in DCM) from 6,100–35,000 cm^{-1}. An empirical absorption correction of ε + 1.61 mol^{-1} dm^{3} cm^{-1} has been applied.

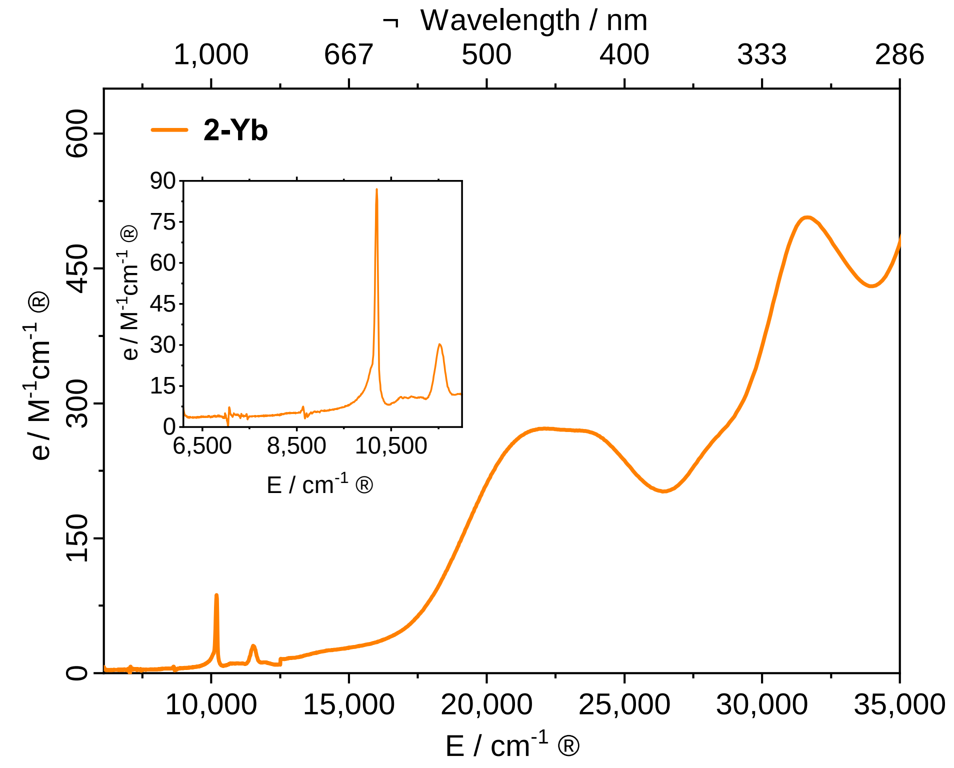

Figure S23. Room temperature UV-vis-NIR spectrum of **2-Yb** (1 mM in DCM) from 6,100–35,000 cm^{-1}. An empirical absorption correction of ε + 1.62 mol^{-1} dm^3 cm^{-1} has been applied.

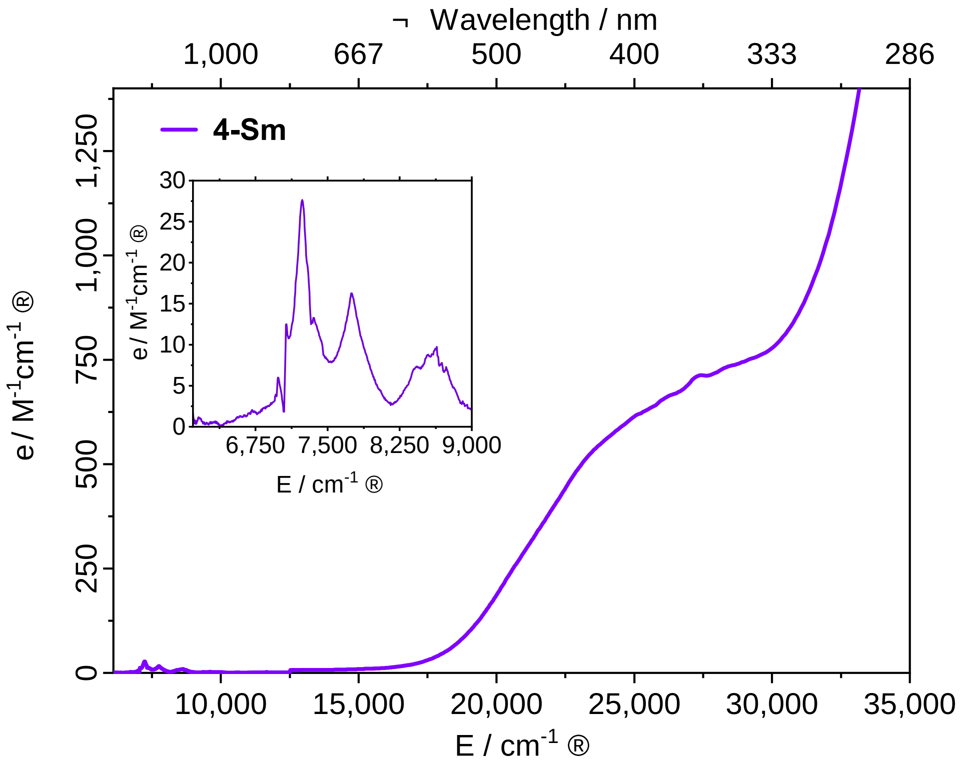

Figure S24. Room temperature UV-vis-NIR spectrum of **4-Sm** (1 mM in DCM) from 6,100–35,000 cm^{-1}. An empirical absorption correction of ε + 1.9 mol^{-1} dm^3 cm^{-1} has been applied.

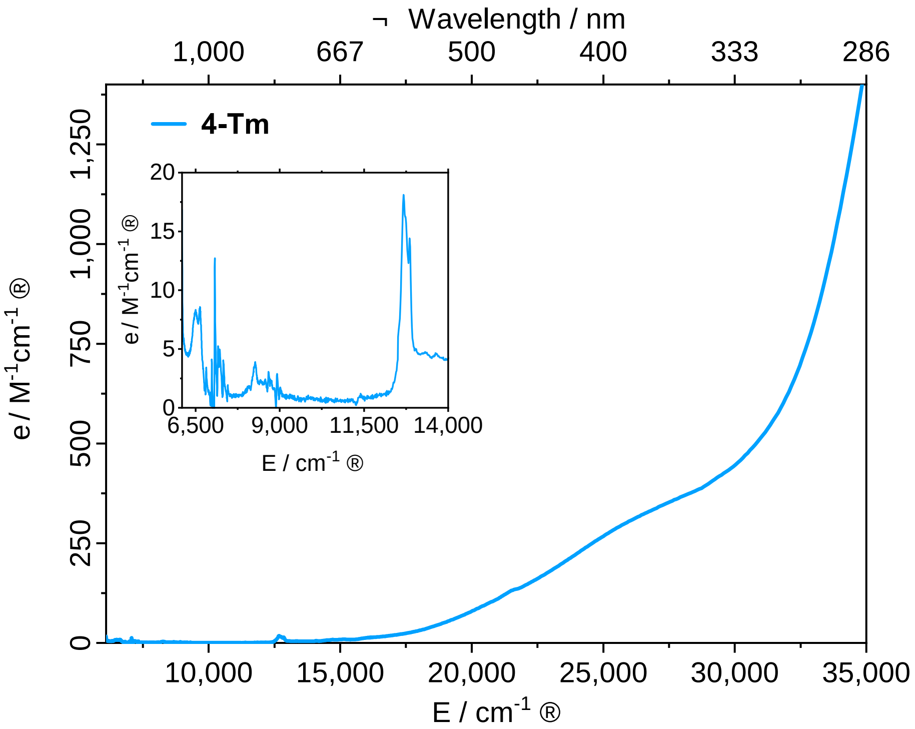

Figure S25. Room temperature vis-NIR spectrum of **4-Tm** (1 mM in DCM) from 6,100–35,000 cm^{-1}. An empirical absorption correction of ε + 1.53 mol^{-1} dm^{3} cm^{-1} has been applied.

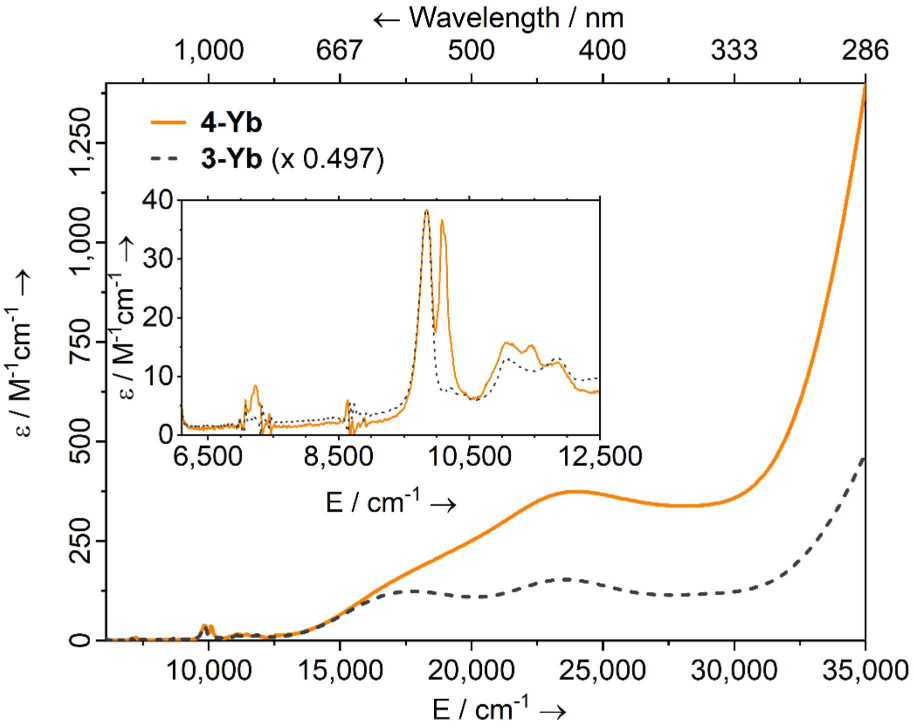

Figure S26. Room temperature vis-NIR spectrum of **4-Yb** (1 mM in DCM) and **3-Yb** (ref [1]) from 6,100–35,000 cm^{-1}. An empirical absorption correction of ε + 0.07 mol^{-1} dm^{3} cm^{-1} has been applied to the **4-Yb** spectrum.

7. EPR Spectroscopy

To model the **2-Yb** X-band EPR spectrum the radical region and the rest of the spectrum (Yb contribution) were simulated separately. The relative contributions of the two crystallographically independent molecules of **2-Yb** (A and B) were fixed at 2/3:1/3. Both $S = ½$ contributions were required to accurately reproduce the inflection at 1275 mT and shoulder at 1380 mT. An *Opt.GridSize* of 361 was used corresponding to 0.25° increments in the powder average. To calculate the TEMPO$^•$ contribution, the simulated Yb contribution was subtracted from the experimental spectrum (both normalized to the minimum at 1346 mT). The resulting spectra, trimmed to 318–351 mT, was used in a least squares fitting using the program esfit in *EasySpin*, normalizing to the maximum TEMPO peak intensity. The TEMPO$^•$ radical was simulated as an anisotropic $S = ½$ with hyperfine coupling to ^{14}N, with a Lorentzian linewidth to model isotropic line broadening (Table S3). The amount of free TEMPO radical relative to **2-Yb** was determined as follows: 1) the Yb and TEMPO$^•$ simulations were performed over the same field positions as the experiment, 2) the Yb simulation was normalized to the experimental minimum at 1346 mT and the TEMPO simulation was multiplied by the same factor 3) an additional weighting factor was applied to the TEMPO$^•$ contribution to represent its abundance in the overall spectrum, 4) the final simulated spectrum was obtained by summing the Yb and TEMPO$^•$ contributions, 5) the TEMPO$^•$ weighting factor was optimized (0.280%) to minimize the sum of squared errors between the final simulation and experimental spectrum. The final parameters are given in Table S3.

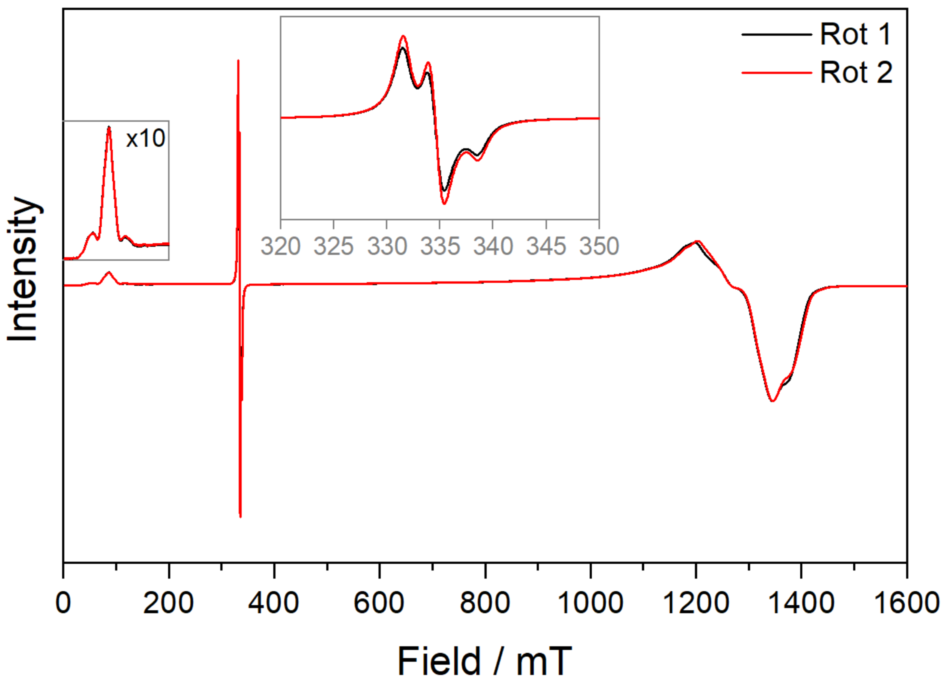

Figure S27. X-band (rot 1: 9.389592 GHz, rot 2: ν = 9.389879 GHz) powder EPR spectra of **2-Yb** at 8 K recorded for two approximately perpendicular sample rotations. Insets show low field region and radical region.

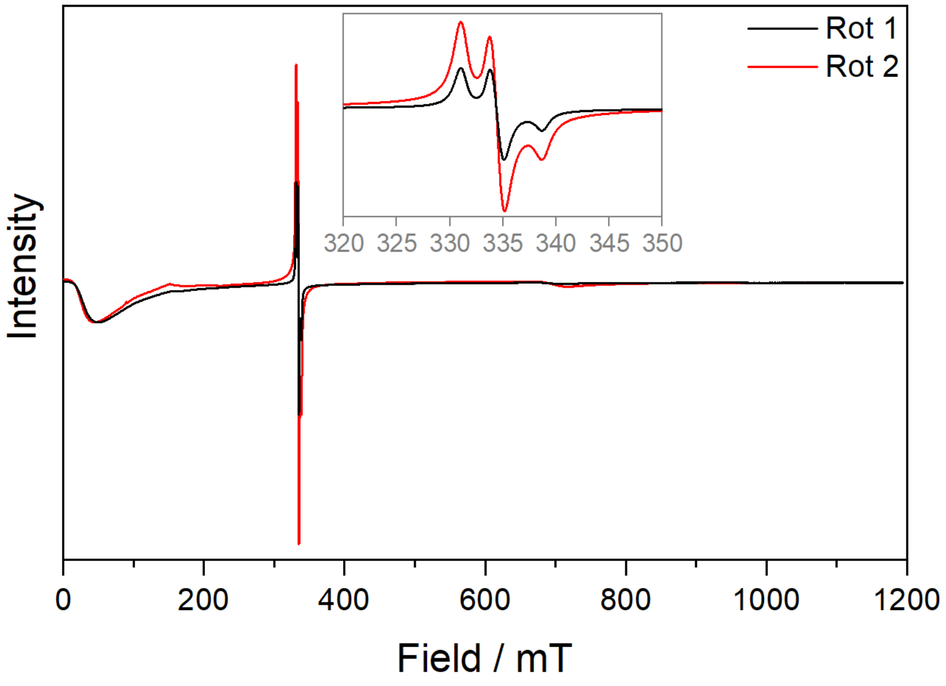

Figure S28. X-band powder EPR spectrum of **4-Yb** at 6 K recorded for two approximately perpendicular sample rotations (ν = 9.384964, 9.38488 GHz for rot 1 and 2). Inset shows radical region.

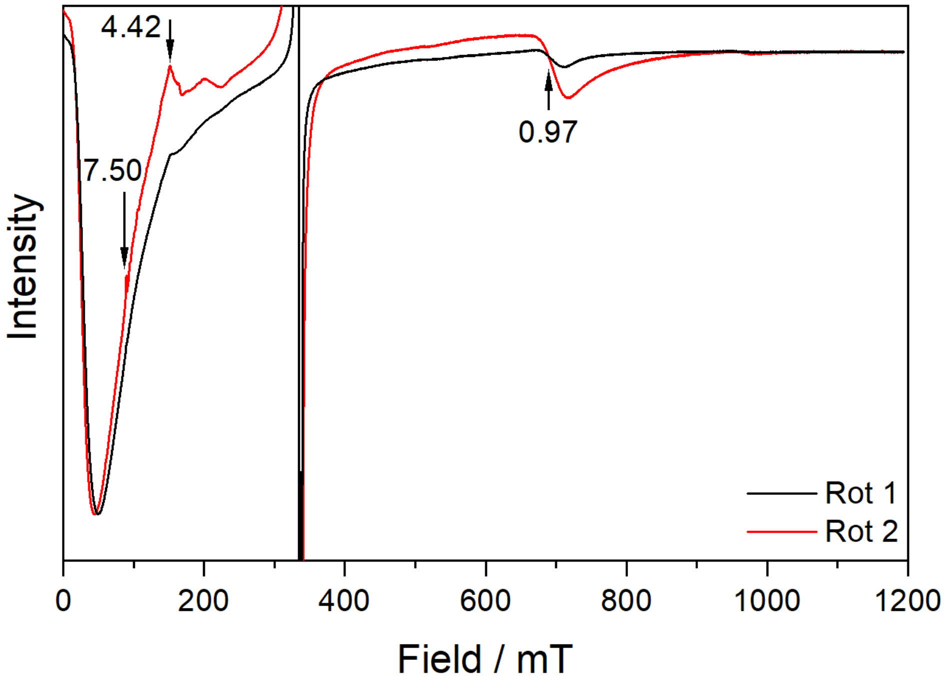

Figure S29. X-band powder EPR spectrum of **4-Yb** at 6 K recorded for two approximately perpendicular sample rotations showing impurities in the baseline.

Table S3. Simulation parameters for X-band EPR spectra of **2-Yb** with TEMPO$^{\bullet}$ impurity.

**Parameter**	**2-Yb** (molecule A)	**2-Yb** (molecule B)	**TEMPO$^{\bullet}$**
g_1	7.755	7.755	2.0091
g_2	0.540	0.512	2.0059
g_3	0.499	0.484	2.0017
Δg_1	1.67	1.67	n/a
Δg_2	0.0325	0.019	n/a
Δg_3	0.018	0.013	n/a
Lorentzian linewidth	n/a	n/a	1.245
A_1 (^{171}Yb/^{173}Yb) or ^{14}N (MHz)	6784 / 1781	6784 / 1781	24.1
A_2 (^{171}Yb/^{173}Yb) or ^{14}N (MHz)	409 / 107	384 / 101	13.4
A_3 (^{171}Yb/^{173}Yb) or ^{14}N (MHz)	372 / 98	359 / 94	99.1
Weighting	2/3	1/3	0.00280

8. Magnetism

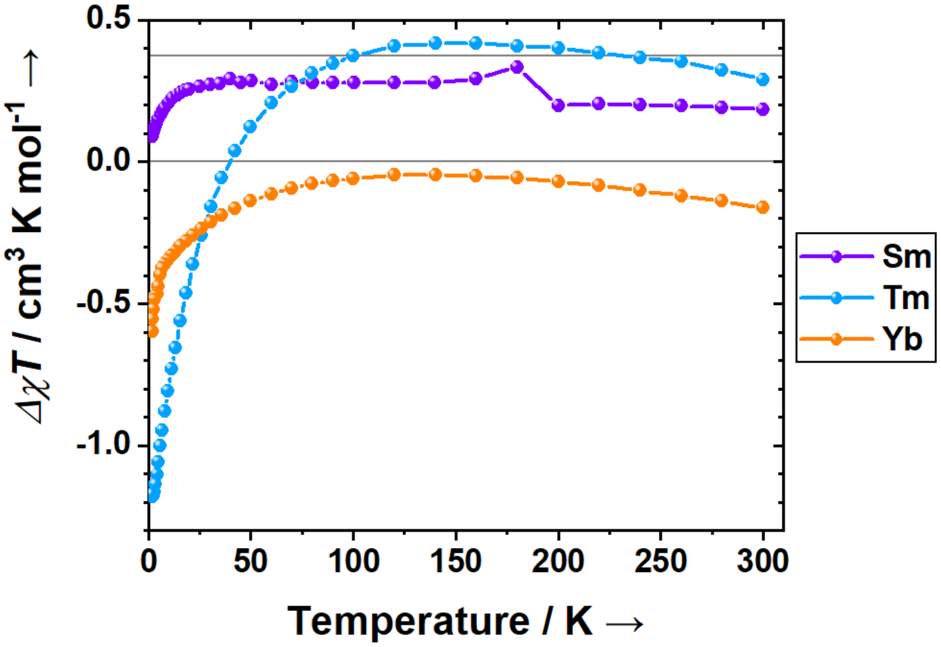

Figure S30. Temperature-dependence of the difference in χT between **4-Ln** and **2-Ln**. Horizontal lines at 0 and 0.375 cm^3 K mol^{-1} (S = ½).

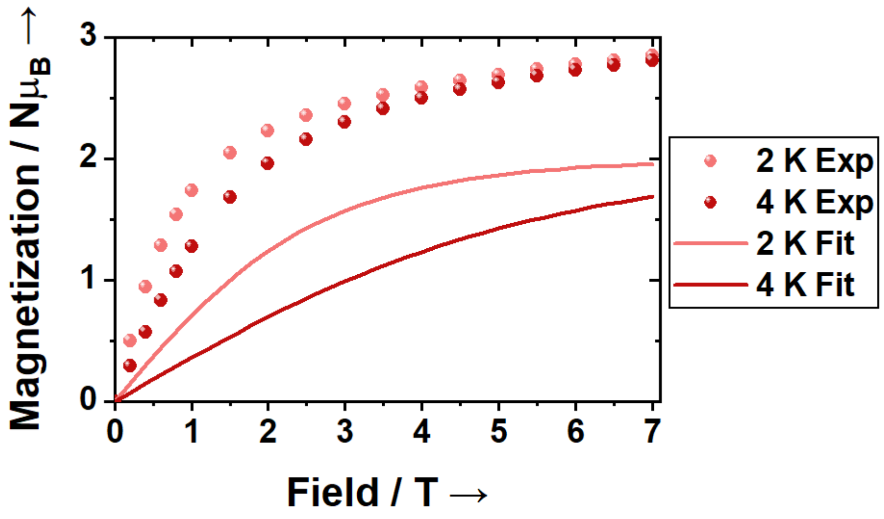

Figure S31. Magnetization *vs.* field for **4-Tm** fitted with an effective S_{eff} = ½ (g_z = 13.9388, g_x = g_y = 0 fixed from CASSCF on **[4-Tm]^0**), radical S = ½ and Ising exchange with J_z optimized to +1.85 cm^{-1}.

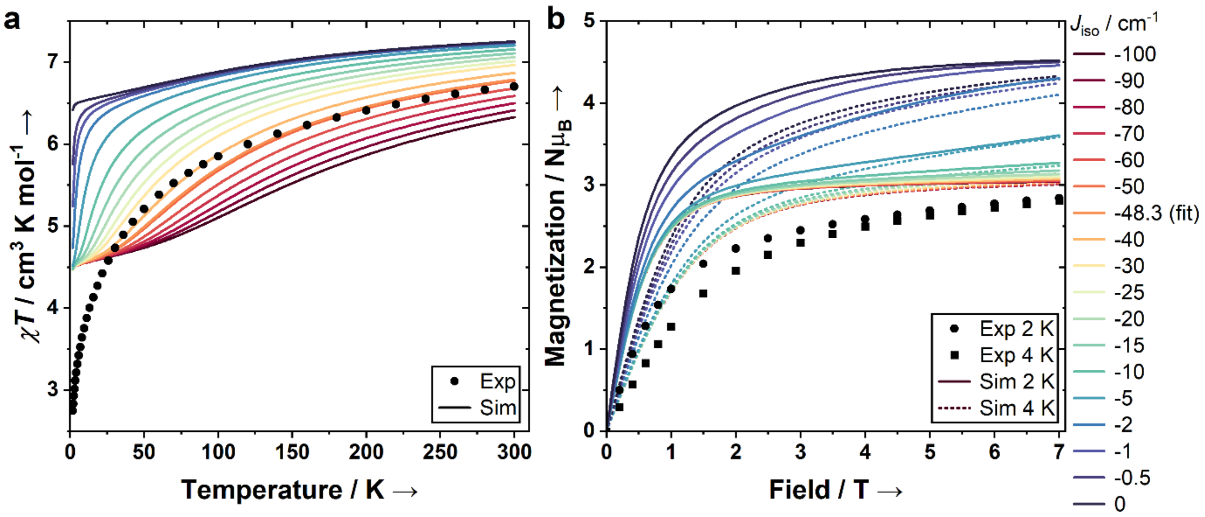

Figure S32. (a) χT *vs.* temperature and (b) magnetization *vs.* field for **4-Tm** showing simulated curves using a S = 1, L = 5 model for Tm^{3+} (crystal field parameters fixed from CASSCF on **[4-Tm]^0**), radical S = ½ and isotropic Lines exchange with J_{iso} between 0 and –100 cm^{-1} (optimized value –48.3 cm^{-1}).

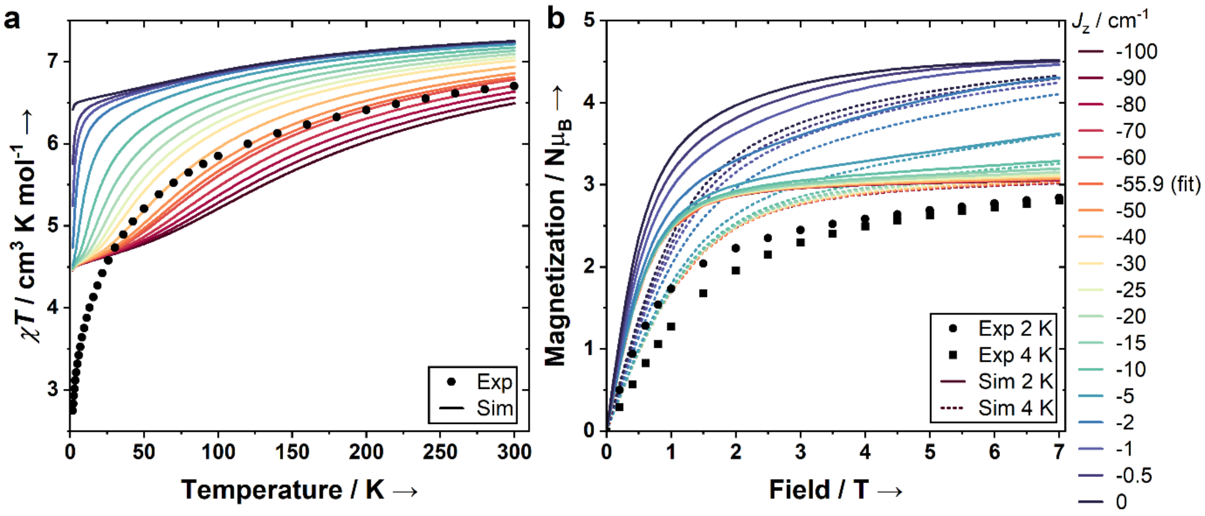

Figure S33. (a) χT *vs.* temperature and (b) magnetization *vs.* field for **4-Tm** showing simulated curves using a S = 1, L = 5 model for Tm^{3+} (crystal field parameters fixed from CASSCF on **[4-Tm]^0**), radical S = ½ and Ising Lines exchange with J_z between 0 and –100 cm^{-1} (optimized value –55.9 cm^{-1}).

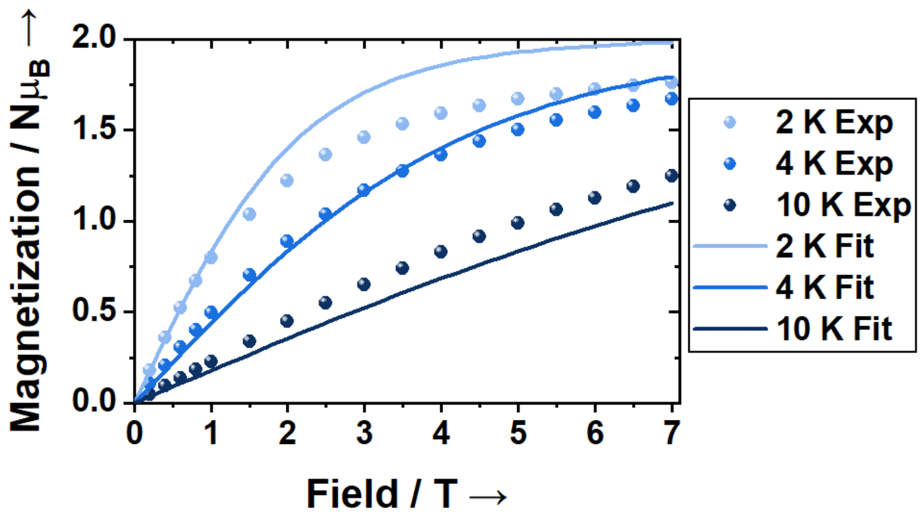

Figure S34. Magnetization *vs.* field for **4-Yb** fitted with an effective S_{eff} = ½ (g_z = 7.755, g_y = 0.5306667, g_x = 0.494 fixed from weighted **2-Yb** experimental values), radical S = ½ and isotropic exchange with J_{iso} optimized to +77.5 cm^{-1}.

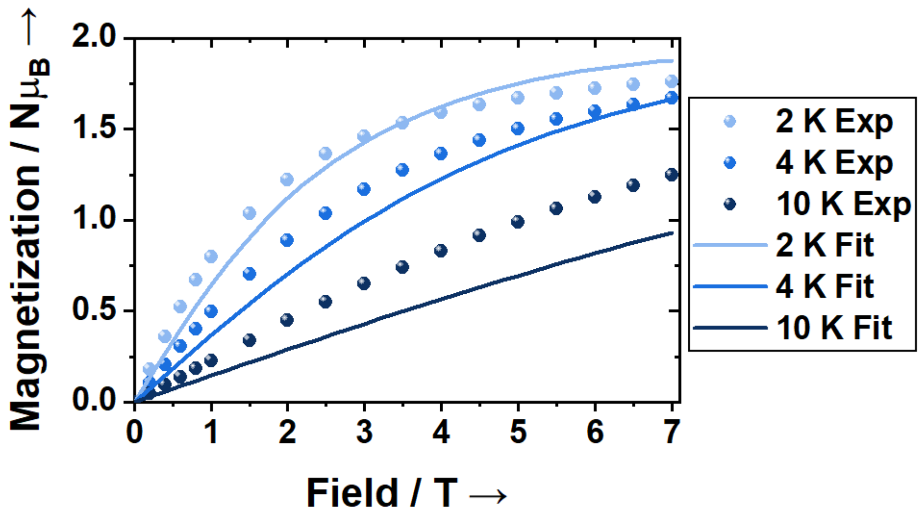

Figure S35. Magnetization *vs.* field for **4-Yb** fitted with an effective S_{eff} = ½ (g_z = 7.755, g_y = 0.5306667, g_x = 0.494 fixed from weighted **2-Yb** experimental values), radical S = ½ and Ising exchange with J_z optimized to +4.57 cm^{-1}.

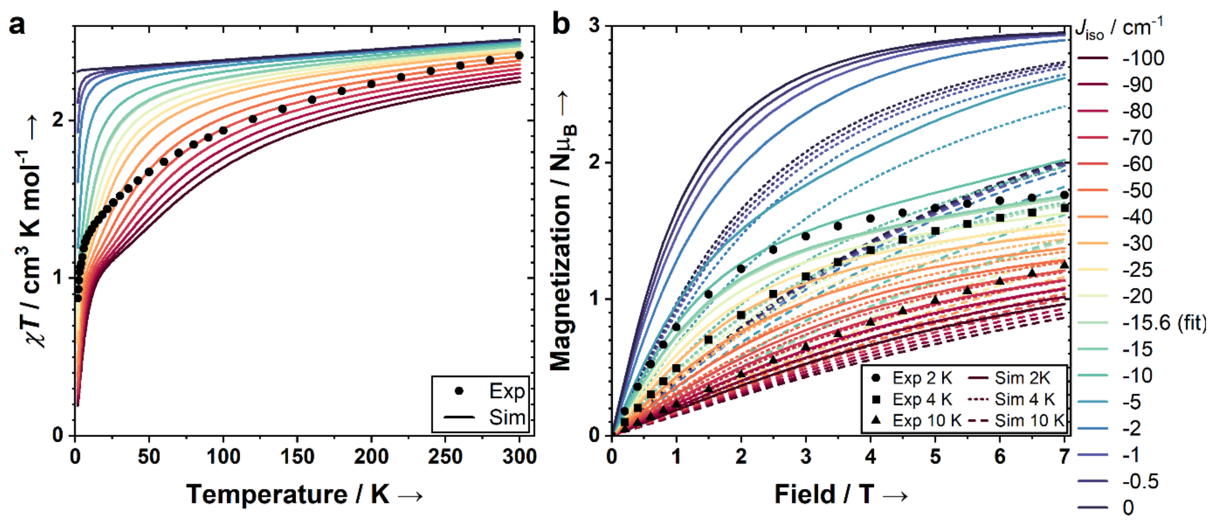

Figure S36. (a) χT *vs.* temperature and (b) magnetization *vs.* field for **4-Yb** showing simulated curves using a S = ½, L = 3 model for Yb^{3+} (crystal field parameters fixed from CASSCF on **[4-Yb]^0**), radical S = ½ and isotropic Lines exchange with J_{iso} between 0 and –100 cm^{-1} (optimized value –15.6 cm^{-1}).

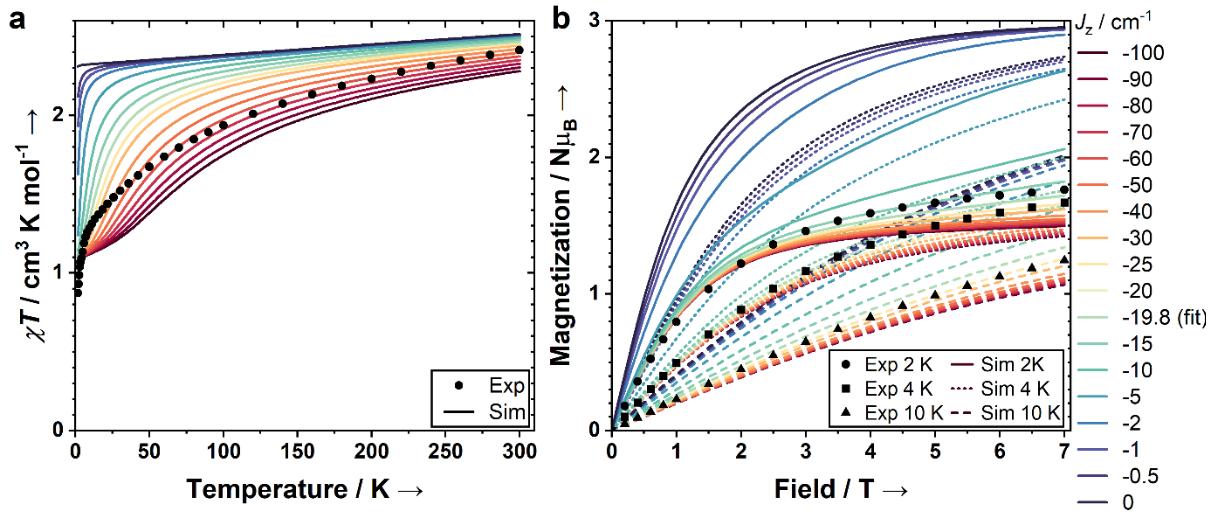

Figure S37. (a) χT *vs.* temperature and (b) magnetization *vs.* field for **4-Yb** showing simulated curves using a S = ½, L = 3 model for Yb^{3+} (crystal field parameters fixed from CASSCF on **[4-Yb]^0**), radical S = ½ and Ising Lines exchange with J_z between 0 and –100 cm^{-1} (optimized value –19.8 cm^{-1}).

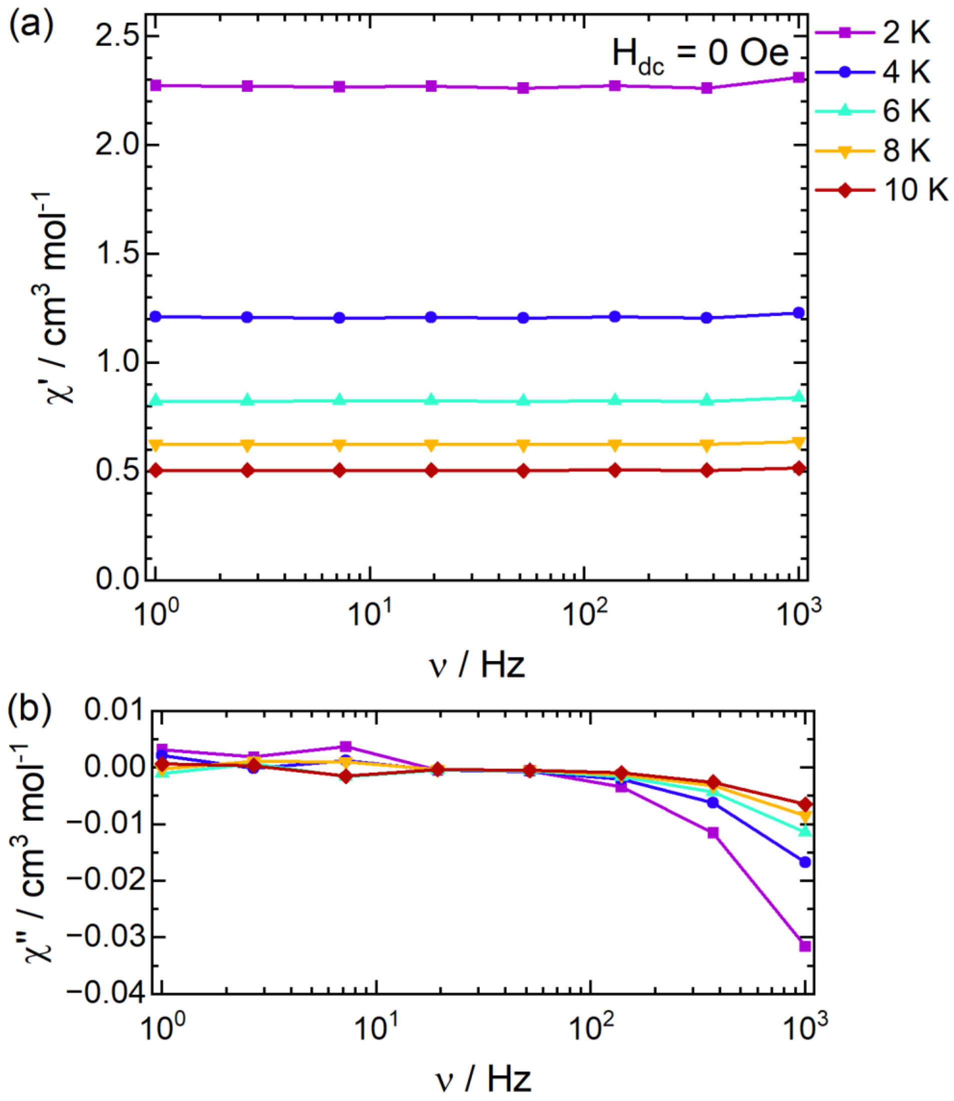

Figure S38. Frequency- and temperature-dependence of (a) in-phase susceptibility, χ′ and (b) out-of-phase susceptibility χ″ for **2-Tm** in zero applied dc field.

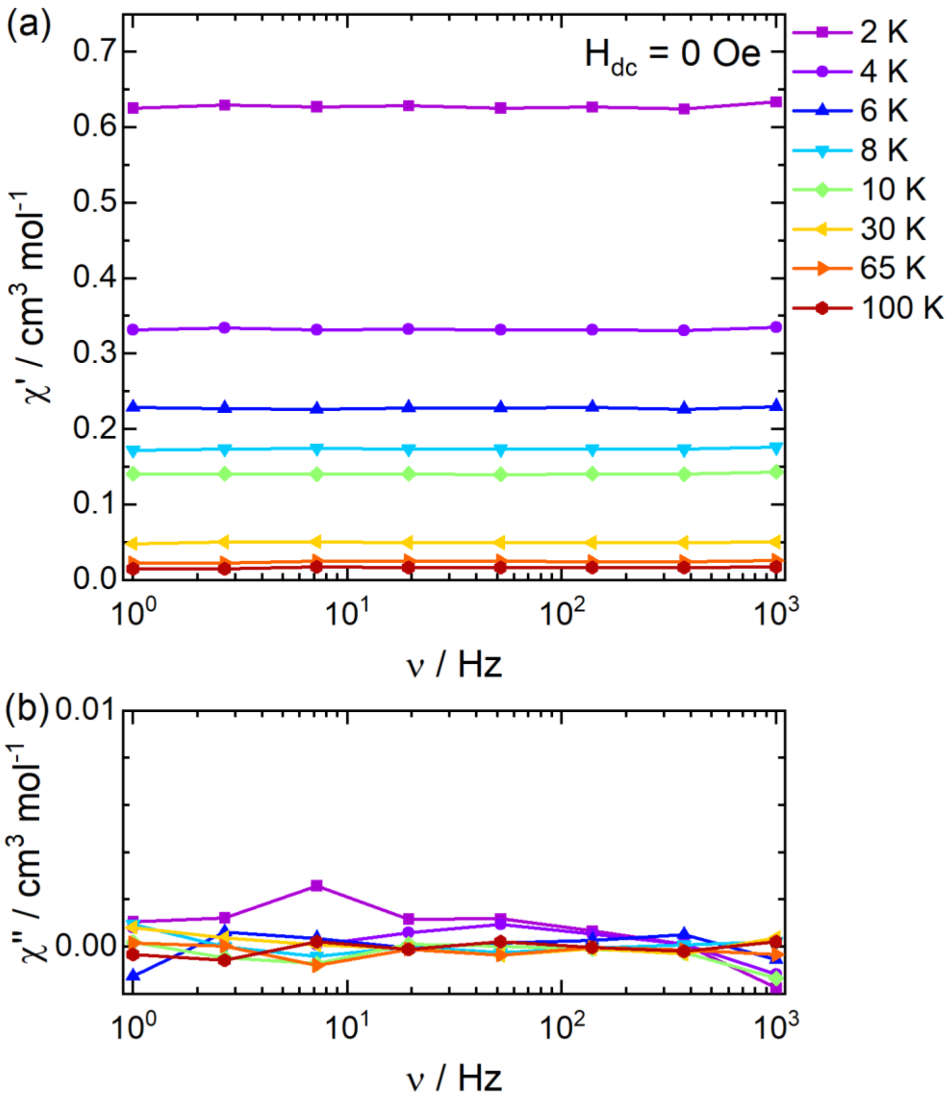

Figure S39. Frequency- and temperature-dependence of (a) in-phase susceptibility, χ′ and (b) out-of-phase susceptibility χ″ for **2-Yb** in zero applied dc field.

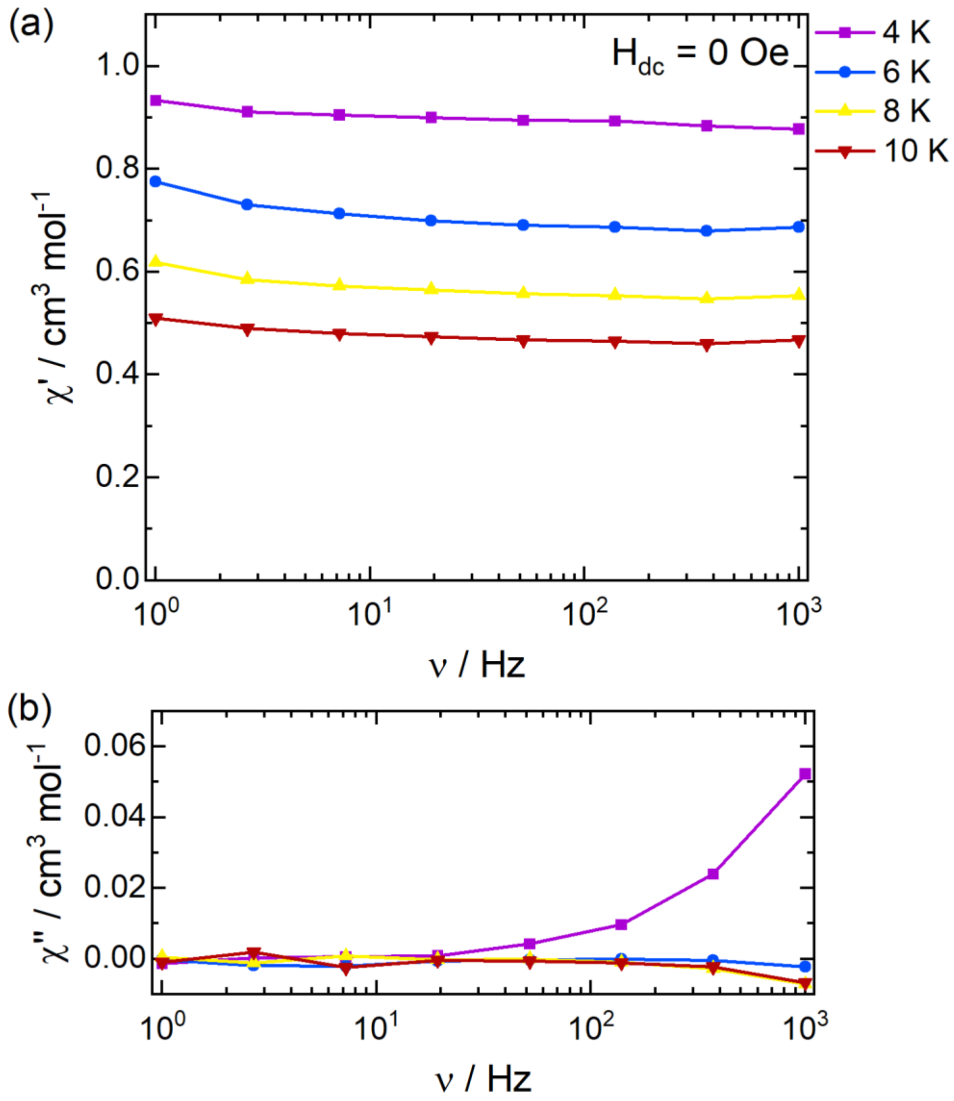

Figure S40. Frequency- and temperature-dependence of (a) in-phase susceptibility, χ′ and (b) out-of-phase susceptibility χ″ for **4-Tm** in zero applied dc field.

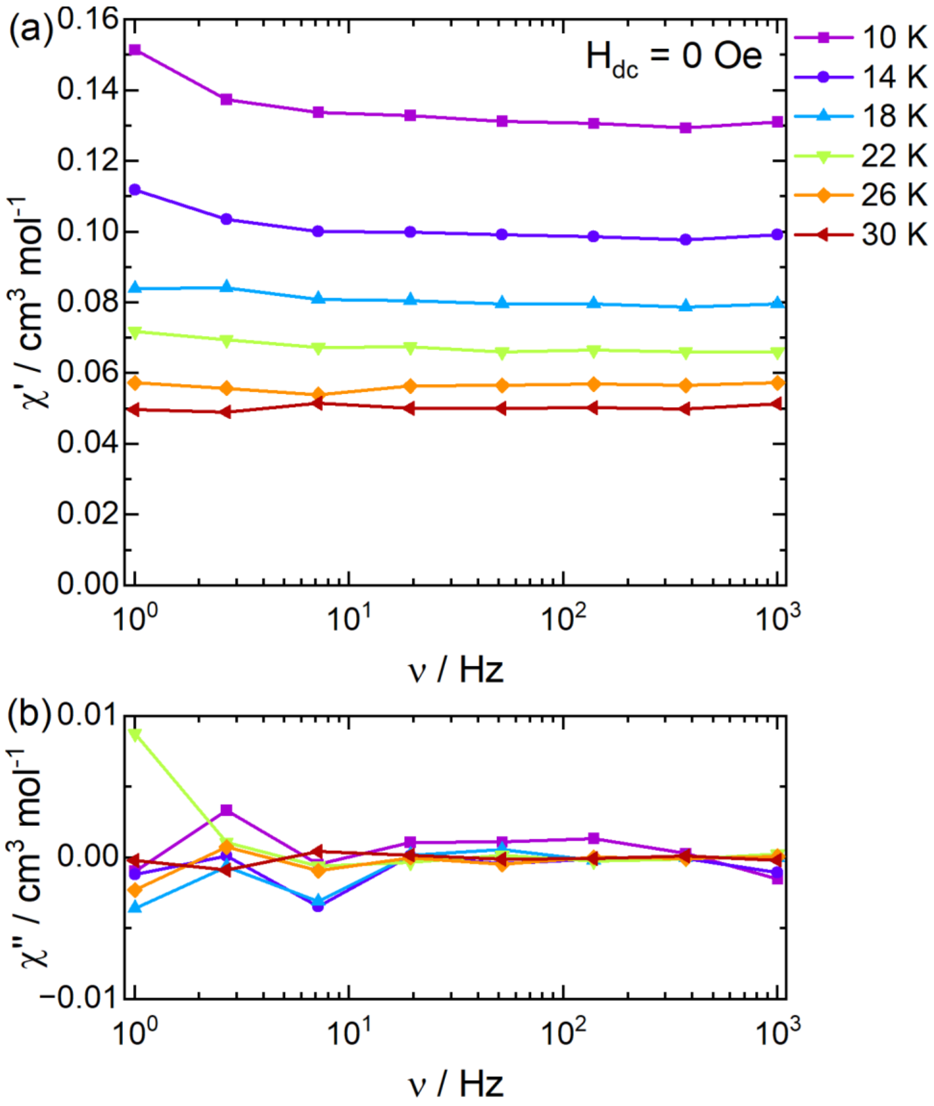

Figure S41. Frequency- and temperature-dependence of (a) in-phase susceptibility, χ′ and (b) out-of-phase susceptibility χ″ for **4-Yb** in zero applied dc field.

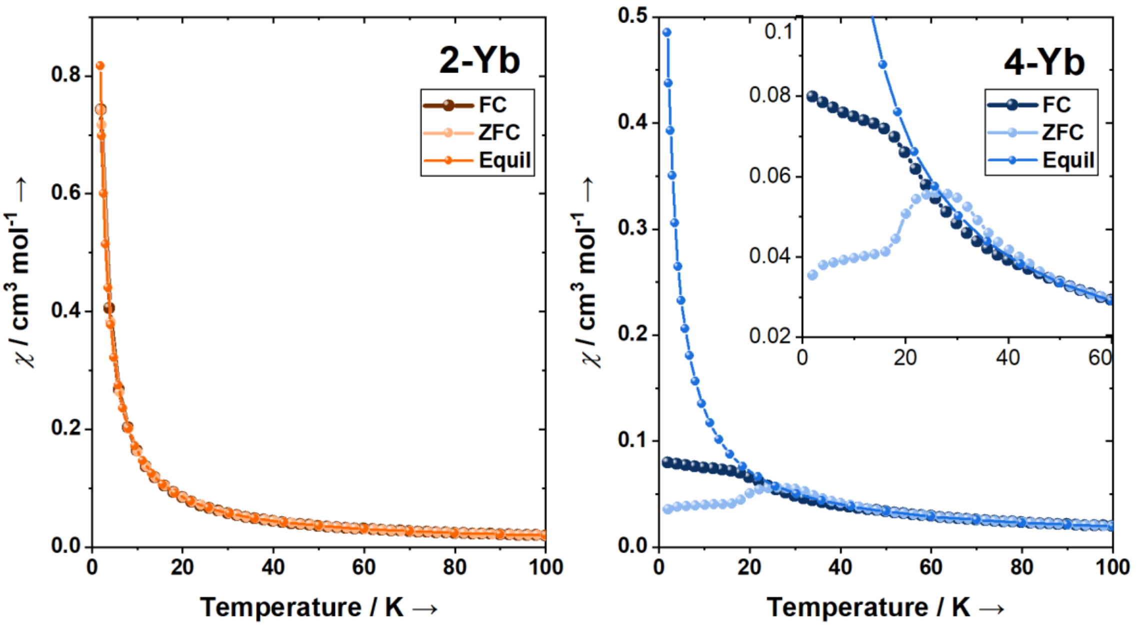

Figure S42. Zero-field cooled (ZFC), field-cooled (FC) and equilibrium susceptibility measurements for **2-Yb** and **4-Yb** in 0.1 T dc field, swept at 0.9 K/min.

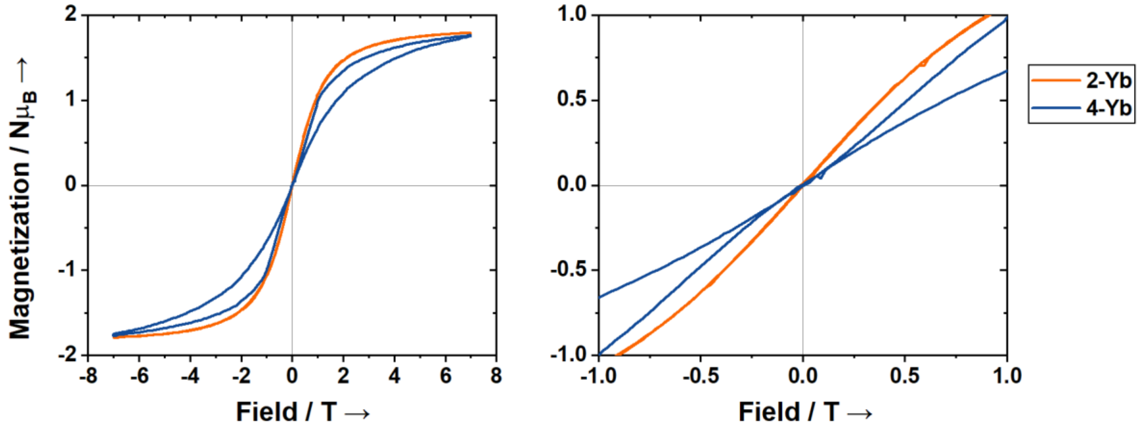

Figure S43. Magnetic hysteresis vs field for **2-Yb** (orange) and **4-Yb** (blue) at 2 K, swept at 22.0 Oe/s for 0 < |H| < 1 T, 51.9 Oe/s for 1 < |H| < 2 T and 90.6 Oe/s for 2 < |H| < 7 T. Right panel shows the closed loops in the low field region.

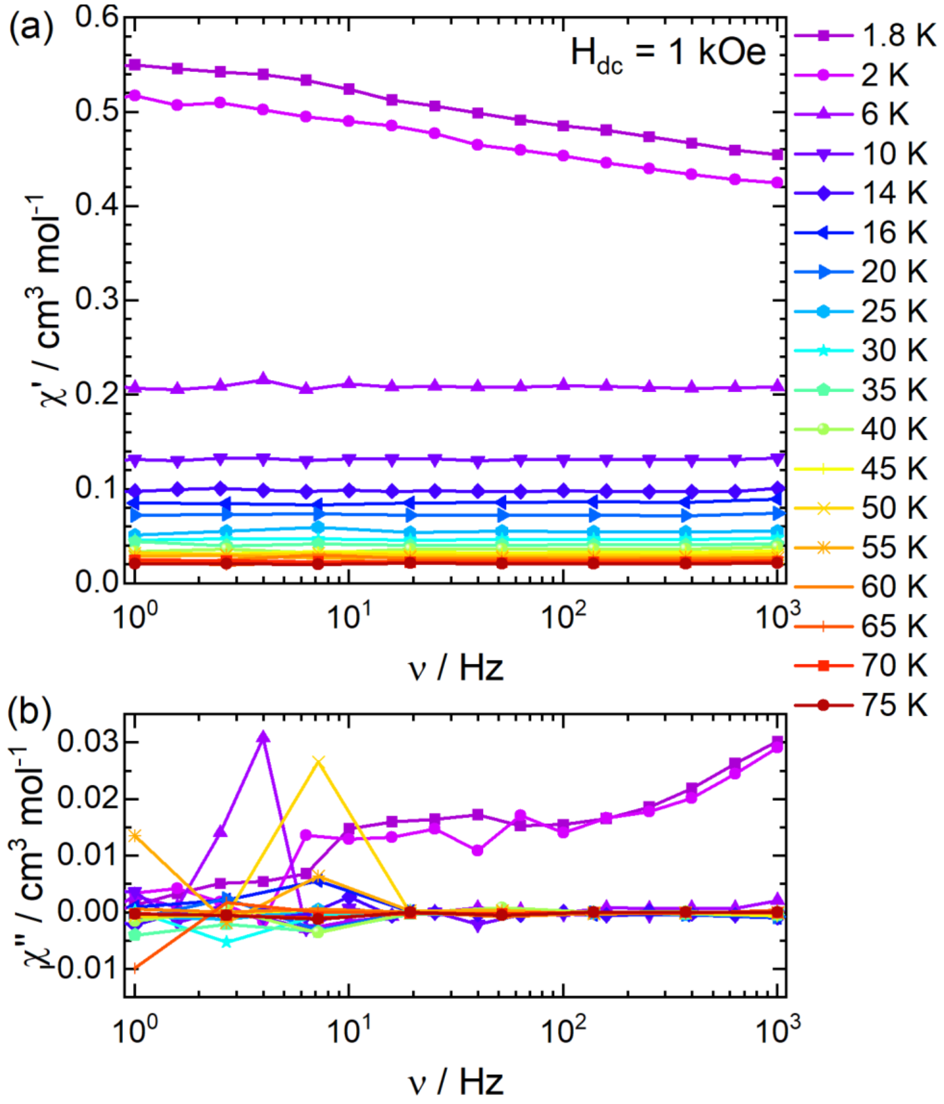

Figure S44. Frequency- and temperature-dependence of (a) in-phase susceptibility, χ′ and (b) out-of-phase susceptibility χ″ for **4-Yb** in 1 kOe applied dc field.

8. CASSCF-SO Electronic Structure

We focused computational efforts to elucidate exchange **4-Yb**, as there is a single ^2F free ion term for Yb(III) which that fixes the number of states to seven singlets and seven triplets for **4-Yb**. XMS-CASPT2 calculations did not converge for **4-Yb**. Calculations on **4-Yb** were attempted to be improved by extending the active space using RAS-probing. Potentially interesting inactive and virtual orbitals were introduced to the RAS1 or RAS3 subspaces respectively in a state-averaged RASSCF calculation (7 triplets or 7 singlets).[2] Up to doublet excitations were allowed into RAS3 and out of RAS1. Yb and TEMPO orbitals were introduced in calculations of **4-Yb**, but the occupancies of these orbitals were ≥1.97 or ≤0.03 and the calculated magnetization was no closer to experiment. RASCI calculations were also performed by localizing the inactive and virtual spaces in the LOCALISATION module using Pipek-Mezey or Pao[3-4] and then moving selected orbitals into RAS1 or RAS3 spaces and performing a CI only calculation. No significant change in magnetic properties or the pattern of exchange states was observed with any tried combination of Yb or TEMPO orbitals.

We attempted to explicitly include configurations with the radical residing in orbitals other than N–O π* to see if this would affect the magnetic properties by simultaneously expanding the active space and increasing the number of averaged states. The $(\text{Yb f})^{13}(\text{O2 p})^1(\text{N–O }\pi^*)^2$ configurations are ~24,000–26,000 cm^{-1} above the ground state, as identified in a CAS(16,9) calculation, averaging over 14 triplets or 14 singlets with an active space including the Yb 4f orbitals, N–O π* antibonding orbital and non-bonding O 2p orbital. The $(\text{Yb f})^{13}(\text{N–O }\pi)^1(\text{O2 p})^2(\text{N–O }\pi^*)^2$ configurations with the radical in the N–O π bonding orbital are ~49,000–51,000 cm^{-1} above the ground state, identified in a CAS(18,10) calculation, averaging over 21 triplets or 21 singlets with an active space as above with the addition of the N–O π bonding orbital. Including any of these additional states did not change the pattern of splitting in the ground state or improve the simulated magnetic properties.

Calculations on **4-Tm** and **4-Sm** were attempted averaging over a smaller number of states i.e. only the states arising from the ^{3}H Tm(III) ground term, ^{6}H Sm(III) ground term or ^{6}H and ^{6}F Sm(III) terms; however, this did not provide better agreement with experimental magnetic data.

All CASSCF calculations support weak exchange in **4-Ln** with states spread over <30 cm^{-1} (Tables S6, S8 and S10). However, CASSCF is unable to accurately determine the exchange couplings in these complexes. CASSCF overpredicts antiferromagnetic exchange for **4-Yb**. The calculated ground state for **4-Yb** is singly degenerate and isolated, inconsistent with EPR and magnetization data. The calculated magnetization has a too small initial slope (χ), inverted temperature dependence (larger magnetization values at higher temperatures) and wrong curvature (slope increases with increasing field) compared to experiment (Figure S47). Calculations on **4-Tm** predict a dominant ferromagnetic interaction. However, the predicted ground state is too magnetic (Figures S46 and S47, Table S8), irrespective of states included in the averaging procedure. Magnetic susceptibility data is well reproduced for **4-Sm**; however, the magnetization data is overpredicted (Figures S46 and S47). The calculated exchange spectra for **4-Sm** were within 11 cm^{-1} with a pseudo-doublet ground state; however, as **4-Sm** is EPR silent, the ground pseudo-doublet is likely split by more than 0.32 cm^{-1}.

Table S4. Computational details for CASSCF-SO calculations.

	Active electrons	Orbitals	Ln(III) free ion terms	Spin multiplicity	Roots	States mixed by spin-orbit coupling
**2-Yb**	13	7	^{2}F	2	7	7
**[4-Yb]^{0}**	13	7	^{2}F	2	7	7
**4-Yb**	14	8	^{2}F	3, 1	7, 7	7, 7
**2-Tm**	12	7	^{3}H, ^{3}F, ^{3}P, ^{1}G, ^{1}I, ^{1}D[a]	3, 1	21, 27	21, 27
**[4-Tm]^{0}**	12	7	^{3}H, ^{3}F, ^{3}P, ^{1}G, ^{1}I, ^{1}D[a]	3, 1	21, 27	21, 27
			^{3}H	3	11	11
**4-Tm**	13	8	^{3}H, ^{3}F, ^{3}P, ^{1}G, ^{1}I, ^{1}D[a]	4, 2	21, 48	21, 48
			^{3}H	4, 2	11, 11	11, 11
**2-Sm**	5	7	All possible	6, 4, 2	21, 224, 490	21, 128, 130
**[4-Sm]^{0}**	5	7	All possible	6, 4, 2	21, 224, 490	21, 128, 130
			^{6}H, ^{6}F	6	18	18
			^{6}H	6	11	11
**4-Sm**	6	8	^{6}H, ^{6}F	7, 5	18, 18	18, 18
			^{6}H	7, 5	11, 11	11, 11

[a] ^{1}S term excluded as it is at >80,000 cm^{-1} compared to <40,000 cm^{-1} for all other states.

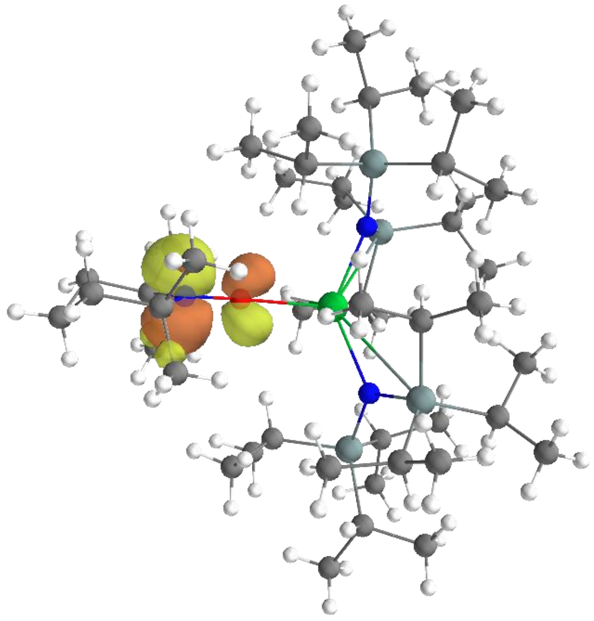

Figure S45. N–O π* orbital (orange/yellow) included in active space for **4-Yb**. Color code: green (Yb), blue (N), red (O), blue-grey (Si), grey (C), white (H). Isosurface is 0.05 e Å^{-3}.

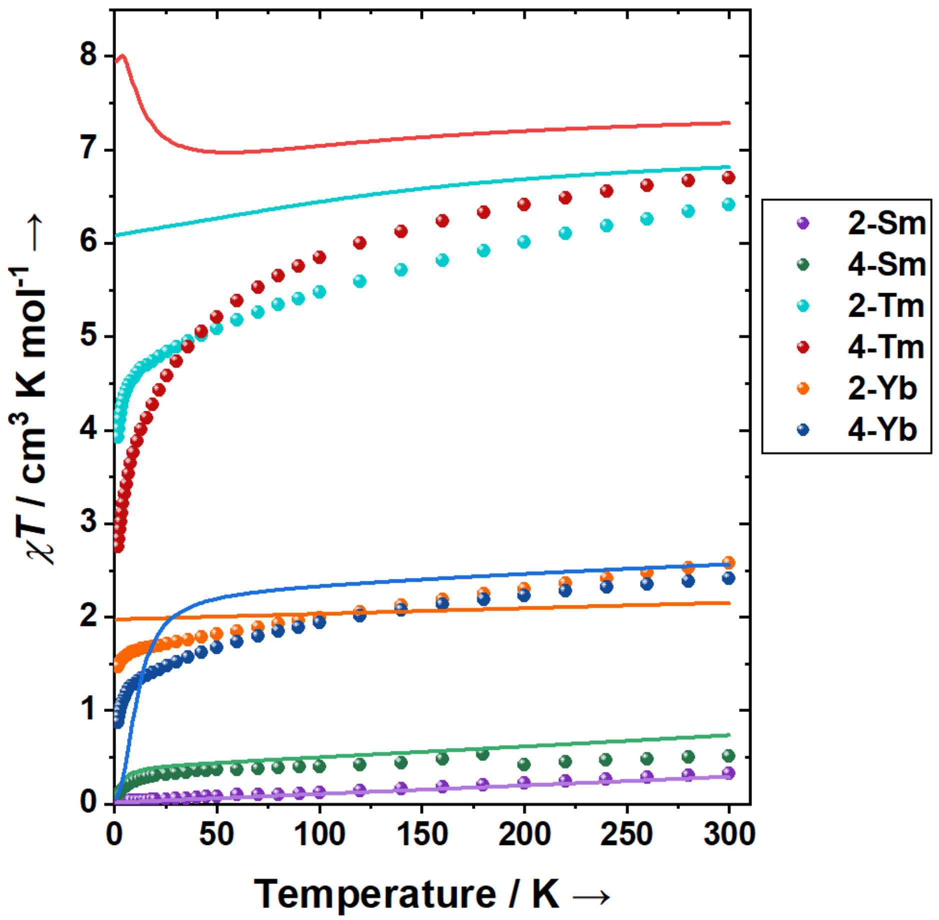

Figure S46. Temperature-dependence of χT for **2-Ln** and **4-Ln**, showing experimental data (points) and CASSCF/RASSI-SO or CASSCF/XMS-CASPT2/RASSI-SO (**2-Yb**) curves. Where multiple calculations were performed the calculation with the maximum number of roots has been selected: 18 septets + 18 quintets for **4-Sm**, 21 triplets + 48 singlets for **4-Tm**.

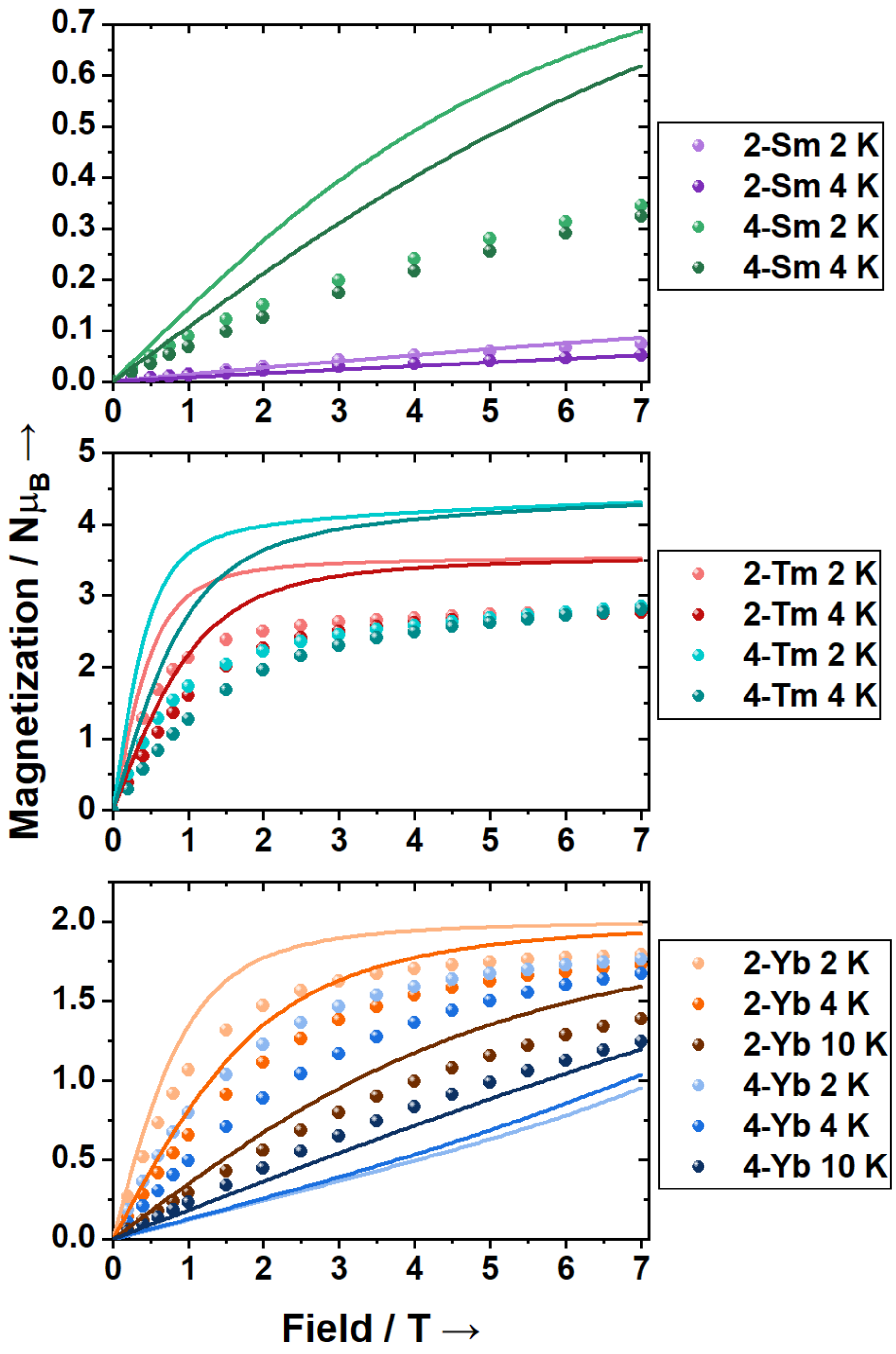

Figure S47. Magnetization vs field plots for **2-Ln** and **4-Ln** (points) with CASSCF/RASSI-SO or CASSCF/XMS-CASPT2/RASSI-SO (**2-Yb**) calculated curves (lines). Where multiple calculations were performed the calculation with the maximum number of roots has been selected: 18 septets + 18 quintets for **4-Sm**, 21 triplets + 48 singlets for **4-Tm**.

Table S5. Composition of $^{2}F_{7/2}$ ground states for **[2-Yb]** & **[4-Yb]⁰**.

**Energy / cm⁻¹**	g_1	g_2	g_3	**Angle / °**	**Wavefunction**	**<Jz>**
**[2-Yb] 7 doublets CASSCF**						
0	7.9628	0.0871	0.0849	-	99.93%|±7/2⟩	±3.498
1054.1231	5.6039	0.5098	0.3610	0.9	99.6%|±5/2⟩	±2.485
1572.2811	3.2744	1.7281	0.8072	4.0	97%|±3/2⟩ + 2%|∓1/2⟩	±1.448
1852.5253	5.7456	3.2302	1.0295	89.4	98%|±1/2⟩ + 2%|∓3/2⟩	±0.461
**[2-Yb] 7 doublets XMS-CASPT2**						
0	7.9272	0.3359	0.3243	-	99.7%|±7/2⟩	±3.483
1399.7448	5.5021	1.0198	0.3725	1.7	98.8%|±5/2⟩	±2.451
1994.3014	3.2684	1.8858	0.4216	8.6	97%|±3/2⟩ + 2%|∓1/2⟩	±1.436
2295.6302	5.6268	3.3382	1/0415	89.3	98%|±1/2⟩ + 2%|∓3/2⟩	±0.469
**[4-Yb]⁰ 7 doublets CASSCF**						
0	7.8932	0.1446	0.1076	-	98%|±7/2⟩ + 1.4%|±3/2⟩	±3.466
1010.3130	5.1850	1.3221	1.1744	0.2	92%|±5/2⟩ + 7%|±1/2⟩ +1.6%|∓3/2⟩	±2.298
1492.3376	4.1854	2.5960	2.1804	89.0	76%|±3/2⟩ + 17%|∓1/2⟩ +5%|∓5/2⟩ + 1.3%|±7/2⟩	±0.964
1979.6919	7.6522	0.5089	0.2946	89.7	76%|±1/2⟩ + 21%|∓3/2⟩ +3%|±5/2⟩	±0.135
**[4-Yb]⁰ 7 doublets XMS-CASPT2**						
0	0.3169	0.4371	7.8341	-	98%|±7/2⟩ + 2%|±3/2⟩	±3.441
1368.509	1.2934	1.5614	5.0057	0.9	90%|±5/2⟩ + 7%|±1/2⟩ +2%|∓3/2⟩	±2.230
1924.338	2.0135	2.4542	4.3912	88.9	74%|±3/2⟩ + 18%|∓1/2⟩ +7%|∓5/2⟩ + 1.3%|±7/2⟩	±0.890
2469.518	0.2290	0.3792	7.6901	89.8	74%|±1/2⟩ + 22%|∓3/2⟩ +3%|±5/2⟩	±0.107

Table S6. Spin-Orbit energies (cm^{-1}) for **2-Yb** and **4-Yb**.

**2-Yb**<br>**CAS(13,7)**<br>**CASSCF**<br>**7 doublets**	**2-Yb**<br>**CAS(13,7)**<br>**XMS-CASPT2**<br>**7 doublets**	**[4-Yb]^0**<br>**CAS(13,7)**<br>**CASSCF**<br>**7 doublets**	**[4-Yb]^0**<br>**CAS(13,7)**<br>**XMS-CASPT2**<br>**7 doublets**	**4-Yb**<br>**CAS(14,8)**<br>**CASSCF**<br>**7 triplets + 7 singlets**
0	0	0	0	0
0	0	0	0	16.5868
				16.6372
				28.6647
1054.1231	1399.7448	1010.3130	1368.5088	872.9699
1054.1231	1399.7448	1010.3130	1368.5088	874.3352
				876.3282
				877.9749
1572.2811	1994.3014	1492.3376	1924.3383	1362.3524
1572.2811	1994.3014	1492.3376	1924.3383	1369.1725
				1369.3802
				1374.4399
1852.5253	2295.6302	1979.6919	2469.5178	1995.4031
1852.5253	2295.6302	1979.6919	2469.5178	1995.7143
				1997.2075
				2003.7863
10498.0039	10582.0921	10495.2117	10585.5108	10470.8193
10498.0039	10582.0921	10495.2117	10585.5108	10484.8427
				10485.5496
				10494.2389
11661.7585	12084.6285	11589.6392	12019.0942	11434.6669
11661.7585	12084.6285	11589.6392	12019.0942	11436.4584
				11439.4484
				11442.1614
12090.2255	12527.9147	12174.5660	12654.9960	12163.8280
12090.2255	12527.9147	12174.5660	12654.9960	12166.7119
				12168.5355
				12172.9987

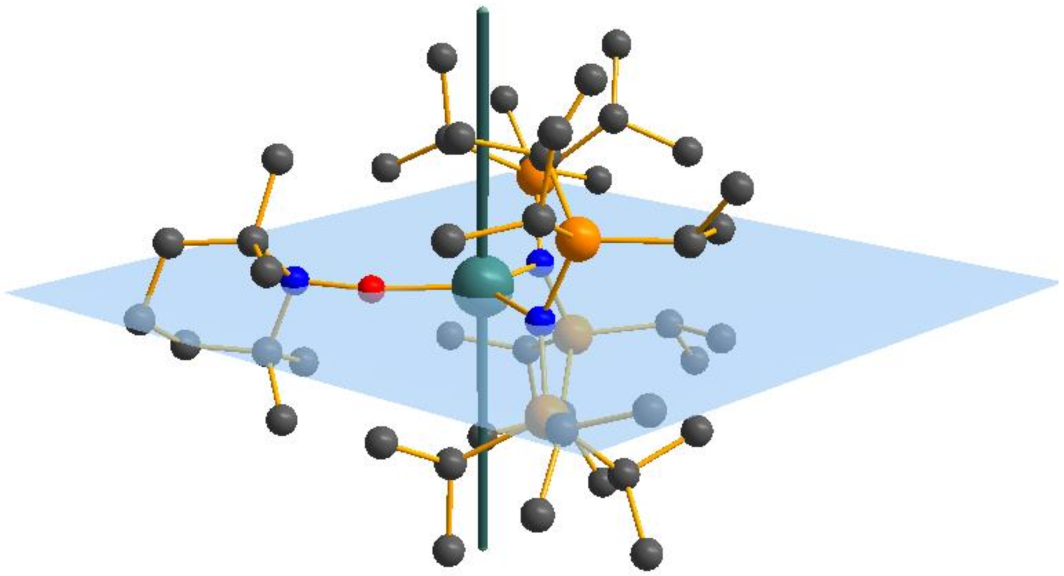

Figure S48. Molecular structure of **2-Tm** showing g_1 main magnetic axis calculated with CASSCF/RASSI-SO (green) and plane through N_2O coordinating atoms (pale blue).

Table S7. Composition of $^3H_6$ states for **[2-Tm]** & **[4-Tm]⁰**.

Energies / cm^{-1}	Wavefunction	g_1
**[2-Tm] 21 triplets + 27 singlets**		
0	$49.98\%\|+6\rangle + 49.98\%\|-6\rangle$	13.9478
0.0430	$49.98\%\|-6\rangle + 49.98\%\|+6\rangle$	
392.7425	$49.88\%\|+5\rangle + 49.88\%\|-5\rangle$	11.5813
393.3278	$49.91\%\|-5\rangle + 49.91\%\|+5\rangle$	
847.0538	$49.5\%\|+4\rangle + 49.5\%\|-4\rangle$	9.1980
852.0131	$49.8\%\|-4\rangle + 49.8\%\|+4\rangle$	
1216.0175	$49.1\%\|+3\rangle + 49.1\%\|-3\rangle$	6.8618
1255.6599	$49.6\%\|-3\rangle + 49.6\%\|+3\rangle$	
1492.1880	$48.3\%\|+2\rangle + 48.3\%\|-2\rangle + 2.3\%\|0\rangle$	4.5221
1534.3082	$49.1\%\|-2\rangle + 49.1\%\|+2\rangle + 1.3\%\|0\rangle$	
1653.5473	$49.2\%\|+1\rangle + 49.2\%\|-1\rangle$	2.3284
1709.4964	$49.5\%\|-1\rangle + 49.5\%\|+1\rangle$	
1739.9790	$95.8\%\|0\rangle + 1.9\%\|+2\rangle + 1.9\%\|-2\rangle$	-

**Energies / cm^{-1}**	**Wavefunction**	$\boldsymbol{g_1}$
**[4-Tm]^0 21 triplets + 27 singlets**		
0	$49.89\%\vert+6\rangle + 49.89\%\vert-6\rangle$	13.9388
0.0487	$49.89\%\vert-6\rangle + 49.89\%\vert+6\rangle$	
367.2698	$49.1\%\vert+5\rangle + 49.1\%\vert-5\rangle$	11.4991
367.8042	$49.1\%\vert-5\rangle + 49.1\%\vert+5\rangle$	
793.8646	$47\%\vert+4\rangle + 47\%\vert-4\rangle + 3\%\vert+2\rangle + 3\%\vert-2\rangle$	8.9145
802.2220	$47\%\vert-4\rangle + 47\%\vert+4\rangle + 3\%\vert-2\rangle + 3\%\vert+2\rangle$	
1131.3400	$35\%\vert+3\rangle + 35\%\vert-3\rangle + 14\%\vert+1\rangle + 14\%\vert-1\rangle$	6.0303
1167.6892	$45\%\vert+3\rangle + 45\%\vert-3\rangle + 4\%\vert+1\rangle + 4\%\vert-1\rangle$	
1318.6231	$37\%\vert0\rangle + 29\%\vert+2\rangle + 29\%\vert-2\rangle + 3\%\vert+4\rangle + 3\%\vert-4\rangle$	-
1531.4414	$47\%\vert-2\rangle + 47\%\vert+2\rangle + 3\%\vert-4\rangle + 3\%\vert+4\rangle$	-
1555.2126	$36\%\vert+1\rangle + 36\%\vert-1\rangle + 14\%\vert+3\rangle + 14\%\vert-3\rangle$	-
1943.9268	$46\%\vert-1\rangle + 46\%\vert+1\rangle + 4\%\vert-3\rangle + 4\%\vert+3\rangle$	-
1944.7287	$62\%\vert0\rangle + 18\%\vert+2\rangle + 18\%\vert-2\rangle$	-
**[4-Tm]^0 11 triplets**		
0	$49.91\%\vert+6\rangle + 49.91\%\vert-6\rangle$	13.9563
0.0417	$49.91\%\vert-6\rangle + 49.91\%\vert+6\rangle$	
445.499	$49.2\%\vert+5\rangle + 49.2\%\vert-5\rangle$	11.5148
446.0182	$49.2\%\vert-5\rangle + 49.2\%\vert+5\rangle$	
945.0075	$47\%\vert+4\rangle + 47\%\vert-4\rangle + 3\%\vert+2\rangle + 3\%\vert-2\rangle$	8.6030
953.6105	$47\%\vert-4\rangle + 47\%\vert+4\rangle + 3\%\vert-2\rangle + 3\%\vert+2\rangle$	
1341.6961	$37\%\vert+3\rangle + 37\%\vert-3\rangle + 12\%\vert+1\rangle + 12\%\vert-1\rangle$	6.1040
1376.0468	$45\%\vert+3\rangle + 45\%\vert-3\rangle + 4\%\vert+1\rangle + 4\%\vert-1\rangle$	
1559.7244	$36\%\vert0\rangle + 29\%\vert+2\rangle + 29\%\vert-2\rangle + 2\%\vert+4\rangle + 2\%\vert-4\rangle$	-
1784.5726	$47\%\vert+2\rangle + 47\%\vert-2\rangle + 3\%\vert+4\rangle + 3\%\vert-4\rangle$	-
1815.6914	$37\%\vert+1\rangle + 37\%\vert-1\rangle + 13\%\vert+3\rangle + 13\%\vert-3\rangle$	-
2240.671	$46\%\vert+1\rangle + 46\%\vert-1\rangle + 4\%\vert+3\rangle + 4\%\vert-3\rangle$	-
2241.7752	$63\%\vert0\rangle + 18\%\vert+2\rangle + 18\%\vert-2\rangle$	-

Table S8. Spin-Orbit state energies (cm^{-1}) below 30,000 cm^{-1} for **2-Tm** and **4-Tm**

**2-Tm CAS(12,7) 21 triplets + 27 singlets**	**[4-Tm]^0 CAS(12,7) 21 triplets + 27 singlets**	**4-Tm CAS(13,8) 21 quartets + 48 doublets**	**[4-Tm]^0 CAS(12,7) 11 triplets**	**4-Tm CAS(13,8) 11 quartets + 11 doublets**
0	0	0	0	0
0.0430	0.0487	0	0.0417	0
		12.1368		0.9916
		12.1368		0.9916
392.7425	367.2698	303.64	445.499	365.3954
393.3278	367.8042	303.64	446.0182	365.3954
		312.1086		367.1589
		312.1086		367.1589
847.0538	793.8646	652.8407	945.0075	774.4121
852.0131	802.2220	652.8407	953.6105	774.4121
		679.0757		797.1452
		679.0757		797.1452
1216.0175	1131.3400	876.8166	1341.6961	1038.4675
1255.6599	1167.6892	876.8166	1376.0468	1038.4675
		1037.9346		1205.7255
		1037.9346		1205.7255
1492.1880	1318.6231	1085.4222	1559.7244	1267.3233
		1085.4222		1267.3233
1534.3082	1531.4414	1454.547	1784.5726	1673.6514
		1454.547		1673.6514
1653.5473	1555.2126	1475.1332	1815.6914	1685.0027
		1475.1332		1685.0027
1709.4964	1943.9268	1986.8405	2240.6710	2260.1535
		1986.8405		2260.1535
1739.9790	1944.7287	2001.6441	2241.7752	2262.9986
		2001.6441		2262.9986
7679.0833	7649.3412	7533.2299	8126.4413	8104.9412
		7533.2299		8104.9412
7699.7235	7649.4771	7542.0402	8126.4884	8105.6599
		7542.0402		8105.6599
7729.7895	7704.0344	7608.6009	8667.6150	8557.0731
		7608.6009		8557.0731
7740.3171	7716.3928	7618.3294	8668.9346	8564.5475
		7618.3294		8564.5475
7765.8125	7771.8768	7688.4074	9141.9808	8903.5942
		7688.4074		8903.5942
7777.6004	7781.9823	7694.2219	9159.7437	9008.4659
		7694.2219		9008.4659
7792.0185	7810.3508	7742.4667	9445.0184	9154.1891
		7742.4667		9154.1891
7852.1540	7854.2980	7779.0139	9633.2088	9519.1553
		7779.0139		9519.1553
7864.2357	7860.2150	7793.6032	9701.4834	9533.6313
		7793.6032		9533.6313

8377.5147	8375.0633	8364.8362	10140.5025	10153.0781
		8364.8362		10153.0781
8379.8028	8375.1627	8367.4224	10144.1045	10155.2006
		8367.4224		10155.2006
8949.4842	8917.0550	8807.2393	14845.7112	14812.6759
		8807.2393		14812.6759
8951.1834	8918.9838	8822.2844	14847.7281	14817.2831
		8822.2844		14817.2831
9388.8146	9320.0542	9111.2711	15492.9785	15326.3516
		9111.2711		15326.3516
9397.4455	9346.3786	9222.5712	15508.7334	15379.3996
		9222.5712		15379.3996
9675.4424	9533.6787	9303.3092	15996.8970	15703.6991
		9303.3092		15703.6991
9712.7936	9693.2833	9607.2126	16154.9916	16042.6407
		9607.2126		16042.6407
9862.1554	9737.5071	9616.0742	16311.1837	16098.8330
		9616.0742		16098.8330
9921.6608	10127.1455	10158.7379	16791.0603	16806.7976
		10158.7379		16806.7976
9965.5566	10130.2342	10161.6356	16804.1103	16810.6081
		10161.6356		16810.6081
13842.3869	13832.4945	13775.1973		
		13775.1973		
13843.7113	13832.8531	13776.9517		
		13776.9517		
14063.7179	14035.9807	13928.8356		
		13928.8356		
14107.5841	14058.2033	13952.4274		
		13952.4274		
14384.1219	14300.2098	14122.0016		
		14122.0016		
14409.8746	14391.3218	14316.4338		
		14316.4338		
14520.9467	14449.1094	14325.4457		
		14325.4457		
14543.5547	14650.5772	14632.8605		
		14632.8605		
14570.5726	14653.9521	14635.4365		
		14635.4365		
17075.5711	17039.9829	16924.3490		
		16924.3490		
17092.8600	17041.0452	16935.4293		
		16935.4293		
17104.0286	17143.5415	17103.4477		
		17103.4477		
17320.3920	17317.2823	17218.1860		
		17218.1860		
17329.9604	17358.1250	17315.3793		
		17315.3793		

17659.3386	17650.2032	17536.0896		
		17536.0896		
17672.8168	17659.0884	17538.9550		
		17538.9550		
18239.7808	18229.9118	18152.0252		
		18152.0252		
18500.4564	18390.1487	18246.6926		
		18246.6926		
18535.6362	18650.3221	18646.0338		
		18646.0338		
19077.6571	19078.9939	18960.0026		
		18960.0026		
19089.8661	19082.0227	18980.1592		
		18980.1592		
22688.7199	22679.0428	22596.8381		
		22596.8381		
22693.7850	22682.2746	22602.7356		
		22602.7356		
22728.2692	22726.1498	22653.3885		
		22653.3885		
22758.5988	22746.4377	22662.3173		
		22662.3173		
22849.5432	22842.5608	22720.5804		
		22720.5804		
22911.0168	22849.3280	22731.3350		
		22731.3350		
23068.1904	22964.4298	22812.4975		
		22812.4975		
23127.6536	23221.8956	23178.8774		
		23178.8774		
23198.0232	23244.2182	23190.8664		
		23190.8664		

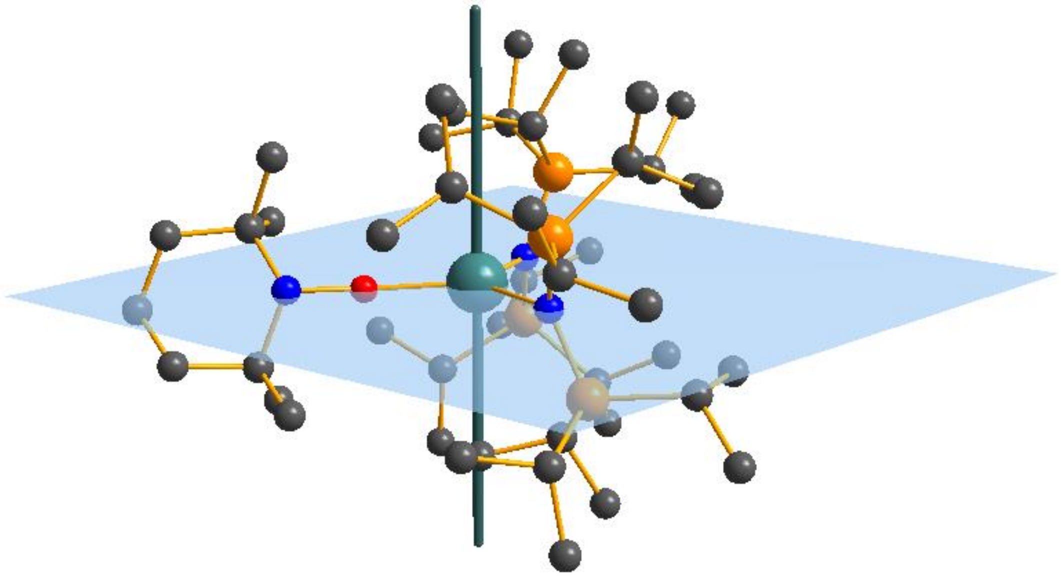

Figure S49. Molecular structure of **2-Sm** showing g_1 main magnetic axis calculated with CASSCF/RASSI-SO (green) and plane through N_2O coordinating atoms (pale blue).

Table S9. Composition of $^6H_{5/2}$ states for **[2-Sm]** & **[4-Sm]⁰**.[a]

**Energy / cm⁻¹**	g_1	g_2	g_3	**Angle / °**	**Wavefunction** [a]	**<J$_z$>** [a]
**[2-Sm] CAS(5,7) 21 sextets + 128 quartets + 130 doublets**						
0	0.6449	0.0433	0.0110	-	98%|±1/2⟩ + 2%|±3/2⟩	±0.519
636.1058	0.7455	0.2312	0.0042	0.5	98%|±3/2⟩ + 2%|±1/2⟩	±1.473
1145.6356	4.6962	0.1221	0.0950	0.8	99.8%|±5/2⟩	±2.492
**[4-Sm]⁰ CAS(5,7) 21 sextets + 128 quartets + 130 doublets**						
0	0.6635	0.0341	0.0146	-	92%|±1/2⟩ + 8%|±3/2⟩	±0.585
595.5478	0.6755	0.6661	0.3203	27.2	90%|±3/2⟩ + 8%|±1/2⟩ +2%|∓5/2⟩	±1.362
1128.2429	3.5742	0.7757	0.0466	2.3	98%|±5/2⟩ + 1%|∓3/2⟩	±2.432
**[4-Sm]⁰ CAS(5,7) 18 sextets**						
0	0.5209	0.0289	0.0121	-	99.7%|±1/2⟩	±0.501
585.1663	0.8994	0.6247	0.2438	1.1	98%|±3/2⟩ + 2%|∓5/2⟩	±1.407
948.1146	4.6071	0.1234	0.0087	0.7	98%|±5/2⟩ + 2%|∓3/2⟩	±2.408
**[4-Sm]⁰ CAS(5,7) 11 sextets**						
0	0.4525	0.0235	0.0085	-	99.9%|±1/2⟩	±0.503
680.9715	0.9746	0.5636	0.2037	1.3	98%|±3/2⟩ + 2%|∓5/2⟩	±1.430
966.1112	4.5635	0.0791	0.0073	0.5	98%|±5/2⟩ + 2%|∓3/2⟩	±2.433

[a] The crystal field parametrization assumes a J = 5/2 ground term, which may not be accurate due to J-mixing from low-lying excited terms.

Table S10. Spin-Orbit state energies (cm^{-1}) below 20,000 cm^{-1} for **2-Sm** and **4-Sm**.

**2-Sm CAS(5,7) 21 sextets + 128 quartets + 130 doublets**	**[4-Sm]^{0} CAS(5,7) 21 sextets + 128 quartets + 130 doublets**	**[4-Sm]^{0} CAS(5,7) 18 sextets**	**4-Sm CAS(6,8) 18 septets + 18 quintets**	**[4-Sm]^{0} CAS(5,7) 11 sextets**	**4-Sm CAS(6,8) 11 septets + 11 quintets**
0	0	0	0	0	0
0	0	0	0.1202	0	0.1233
			6.4731		8.4563
			9.0929		10.8909
636.1058	595.5478	585.1663	472.0779	680.9715	550.3916
636.1058	595.5478	585.1663	475.2275	680.9715	553.5725
			479.4889		558.0678
			483.9322		563.0781
1145.6356	1128.2429	948.1146	934.0911	966.1112	957.6076
1145.6356	1128.2429	948.1146	937.0186	966.1112	960.6122
			938.4586		962.5485
			941.9911		965.7200
1255.6187	1207.6427	1176.1049	1008.9716	1326.5117	1113.2297
1255.6187	1207.6427	1176.1049	1012.7021	1326.5117	1115.4013
			1016.7510		1122.3054
			1019.3927		1126.0683
1705.7705	1727.4423	1560.0454	1494.4366	1676.4362	1592.4526
1705.7705	1727.4423	1560.0454	1496.1133	1676.4362	1594.6410
			1497.4851		1596.0354
			1504.5889		1603.4380
2077.5590	2059.9125	1912.2674	1849.2368	2057.9666	1970.3600
2077.5590	2059.9125	1912.2674	1849.8812	2057.9666	1971.2770
			1852.1399		1971.5006
			1856.6298		1977.6882
2223.5874	2189.5020	2014.0536	1887.8108	2118.1306	2028.6651
2223.5874	2189.5020	2014.0536	1889.3259	2118.1306	2029.7856
			1890.8669		2032.6659
			1892.0150		2032.9998
2425.0049	2422.9012	2088.1119	2080.3260	2196.2330	2108.0410
2425.0049	2422.9012	2088.1119	2081.8044	2196.2330	2110.1183
			2083.7768		2111.7822
			2084.6111		2112.3960
2904.0367	2917.3884	2600.5526	2551.2583	2701.8052	2642.6830
2904.0367	2917.3884	2600.5526	2554.7412	2701.8052	2646.0569
			2560.3286		2652.6426
			2561.2521		2653.5180
3208.7145	3189.5511	2888.0663	2801.5823	3040.6820	2936.2962
3208.7145	3189.5511	2888.0663	2805.2342	3040.6820	2939.8086
			2806.3897		2941.0094
			2807.1783		2941.5991
3400.7406	3363.5100	3062.9367	2980.1837	3235.3806	3120.0732
3400.7406	3363.5100	3062.9367	2983.5879	3235.3806	3123.8997

			2985.8844		3126.1307
			2988.5934		3129.4595
3493.9453	3495.4405	3176.8285	3092.3471	3344.3702	3227.6368
3493.9453	3495.4405	3176.8285	3092.8959	3344.3702	3228.3805
			3094.9529		3230.8633
			3095.2972		3231.2849
3791.7700	3788.9181	3397.0459	3391.8364	3416.6175	3409.2213
3791.7700	3788.9181	3397.0459	3392.1735	3416.6175	3409.5728
			3396.4543		3414.1484
			3396.6394		3414.3778
4212.1303	4221.5297	3850.4025	3806.4545	3937.0503	3883.4600
4212.1303	4221.5297	3850.4025	3808.9832	3937.0503	3886.1447
			3810.0925		3887.5003
			3813.4115		3891.0893
4511.0532	4494.8511	4134.3217	4039.0555	4268.9811	4155.1474
4511.0532	4494.8511	4134.3217	4042.6620	4268.9811	4158.8417
			4043.1930		4159.3915
			4044.1278		4160.1437
4744.1974	4689.5957	4330.3574	4209.5880	4496.3016	4345.8061
4744.1974	4689.5957	4330.3574	4214.2507	4496.3016	4351.1679
			4215.1492		4352.0576
			4216.7171		4353.6915
4889.1374	4851.4773	4494.0633	4427.2622	4669.1509	4574.3429
4889.1374	4851.4773	4494.0633	4428.5086	4669.1509	4575.8675
			4428.7395		4576.4834
			4429.4284		4577.2417
4973.8718	5028.4139	4672.3827	4644.0680	4850.4150	4797.8074
4973.8718	5028.4139	4672.3827	4644.0893	4850.4150	4797.8142
			4645.0408		4799.1476
			4645.0680		4799.2337
5235.8020	5235.2951	4929.9838	4929.3883	4936.8205	4934.6733
5235.8020	5235.2951	4929.9838	4929.4850	4936.8205	4934.7734
			4936.6534		4943.1459
			4936.7096		4943.2562
5585.5766	5591.4587	5304.8771	5268.1195	5374.4604	5328.0514
5585.5766	5591.4587	5304.8771	5269.4854	5374.4604	5329.4810
			5273.8440		5334.2224
			5274.6553		5335.0936
5905.4959	5885.0209	5609.0921	5517.0220	5728.6096	5617.8303
5905.4959	5885.0209	5609.0921	5520.6834	5728.6096	5621.8023
			5522.7599		5623.8618
			5523.7896		5624.9667
6187.8269	6134.3809	5861.3037	5700.1974	6017.2150	5827.3368
6187.8269	6134.3809	5861.3037	5702.9538	6017.2150	5830.7354
			5705.6871		5833.5618
			5706.2774		5834.1168
6387.9273	6305.2257	6034.7851	5913.6208	6208.8790	6058.0838
6387.9273	6305.2257	6034.7851	5915.8682	6208.8790	6060.8786
			5916.0461		6061.1407
			5916.8699		6061.9720

6505.8616	6491.0101	6227.5182	6191.8407	6412.4370	6355.0563
6505.8616	6491.0101	6227.5182	6191.8855	6412.4370	6355.0955
			6196.2315		6359.2194
			6196.6124		6359.6692
6597.5453	6721.9739	6468.9047	6503.9492	6662.8625	6679.7471
6597.5453	6721.9739	6468.9047	6503.9632	6662.8625	6680.0267
			6505.2915		6681.2398
			6505.2945		6681.3598
6747.6467	6757.7451	6713.0198	6731.6555	6701.8575	6721.4806
6747.6467	6757.7451	6713.0198	6731.6862	6701.8575	6721.5333
			6740.7569		6733.3302
			6740.7845		6733.4751
6972.4339	6965.3449	6933.8141	6903.1489	6983.1731	6941.9411
6972.4339	6965.3449	6933.8141	6903.3733	6983.1731	6942.1851
			6914.3683		6954.3738
			6915.1365		6955.2752
7356.9472	7331.5716	7307.8542	7209.7494	7418.5515	7300.4887
7356.9472	7331.5716	7307.8542	7212.1549	7418.5515	7302.9435
			7215.9078		7306.6432
			7220.8235		7311.9731
7686.9730	7637.7178	7622.5701	7441.6316	7770.2648	7562.8230
7686.9730	7637.7178	7622.5701	7442.3671	7770.2648	7563.9874
			7450.9880		7572.9779
			7452.6329		7574.8204
7936.6389	7850.1822	7842.9179	7651.0842	8014.7002	7791.8493
7936.6389	7850.1822	7842.9179	7652.8837	8014.7002	7793.9205
			7654.9037		7796.3150
			7658.6358		7800.0831
8111.9226	8023.4315	8022.7822	7935.0635	8208.7946	8097.5459
8111.9226	8023.4315	8022.7822	7935.6027	8208.7946	8098.1726
			7936.9789		8099.6275
			7937.5607		8100.2876
8230.1650	8252.0635	8261.9415	8276.7525	8463.7132	8462.0397
8230.1650	8252.0635	8261.9415	8276.7599	8463.7132	8462.0610
			8278.5371		8463.7548
			8278.5641		8463.7921
8318.1491	8528.5892	8554.1235	8494.2508	8773.6844	8866.4842
8318.1491	8528.5892	8554.1235	8495.3392	8773.6844	8866.4843
			8496.4349		8880.2455
			8499.5796		8880.2462
8697.0659	8676.5037	8577.2948	8659.7906		
8697.0659	8676.5037	8577.2948	8659.7913		
			8673.9987		
			8674.0019		
8967.9531	8985.3825	8964.1529	8924.4512		
8967.9531	8985.3825	8964.1529	8925.0138		
			8926.3056		
			8930.1105		
9253.0140	9246.7525	9222.0249	9143.1509		
9253.0140	9246.7525	9222.0249	9144.8908		

			9145.0595		
			9146.2860		
9580.9534	9597.5630	9653.1048	9600.8745		
9580.9534	9597.5630	9653.1048	9602.6357		
			9603.7375		
			9604.8902		
9720.6097	9705.5249	9745.2664	9663.7042		
9720.6097	9705.5249	9745.2664	9666.0788		
			9668.2615		
			9668.9547		
9790.0231	9793.4892	9830.6054	9773.7300		
9790.0231	9793.4892	9830.6054	9776.0483		
			9776.7182		
			9777.7003		
10576.2376	10540.1024	10541.3059	10431.0634		
10576.2376	10540.1024	10541.3059	10431.3650		
			10432.0297		
			10432.3500		
10606.7230	10604.0080	10622.9435	10569.3756		
10606.7230	10604.0080	10622.9435	10570.3232		
			10571.6501		
			10573.4459		
10628.2718	10640.6829	10656.4514	10604.6627		
10628.2718	10640.6829	10656.4514	10605.5198		
			10607.1526		
			10607.7842		
10661.8277	10687.5274	10703.9722	10658.3111		
10661.8277	10687.5274	10703.9722	10661.2596		
			10662.2453		
			10663.6968		
11715.5929	11661.7285	11548.8371	11428.7958		
11715.5929	11661.7285	11548.8371	11428.9248		
			11429.6328		
			11429.8506		
11756.3815	11768.2842	11665.6267	11606.1316		
11756.3815	11768.2842	11665.6267	11606.8928		
			11609.5897		
			11612.3840		
11843.8192	11876.1502	11777.0217	11753.5482		
11843.8192	11876.1502	11777.0217	11754.8864		
			11756.5215		
			11762.0392		
11985.8779	11997.2195	11894.6149	11838.5439		
11985.8779	11997.2195	11894.6149	11839.1671		
			11839.3586		
			11843.4161		
12092.9530	12089.7155	11977.8791	11899.7828		
12092.9530	12089.7155	11977.8791	11899.8014		
			11902.1378		
			11902.2541		

13045.7170<br>13045.7170	12972.9199<br>12972.9199	12744.3976<br>12744.3976	12619.8084<br>12619.8183<br>12623.4945<br>12623.5401		
13135.8053<br>13135.8053	13174.2921<br>13174.2921	12951.2726<br>12951.2726	12889.8570<br>12889.9983<br>12891.7736<br>12892.3202		
13268.9335<br>13268.9335	13303.3876<br>13303.3876	13081.1089<br>13081.1089	13056.4972<br>13057.8928<br>13058.3150<br>13062.2627		
13436.9391<br>13436.9391	13448.0722<br>13448.0722	13223.1612<br>13223.1612	13180.2022<br>13182.1091<br>13182.7653<br>13185.4962		
13634.6284<br>13634.6284	13631.0218<br>13631.0218	13399.6091<br>13399.6091	13328.4124<br>13328.6894<br>13331.7203<br>13331.9511		
13860.0207<br>13860.0207	13844.1797<br>13844.1797	13604.2112<br>13604.2112	13511.0415<br>13511.0484<br>13512.6889<br>13512.7291		

Table S11. Crystal Field Parameters for **2-Ln**, **[4-Ln]^0** and the ratio **2-Ln/[4-Ln]^0**.

		**Yb: CAS(13,7) XMS-CASPT2, 7 doublets**			**Tm: CAS(12,7), 21 triplets + 27 singlets**			**Sm: CAS(5,7), 21 sextets + 128 quartets + 130 doublets**		
k	*q*	$B_k^q \theta_k$ **2-Yb**	$B_k^q \theta_k$ **[4-Yb]^0**	**2-Yb/ [4-Yb]^0**	$B_k^q \theta_k$ **2-Tm**	$B_k^q \theta_k$ **[4-Tm]^0**	**2-Tm/ [4-Tm]^0**	$B_k^q \theta_k$ **2-Sm**	$B_k^q \theta_k$ **[4-Sm]^0**	**2-Sm/ [4-Sm]^0**
2	-2	-3.26	4.00E-2	-0.01	-1.73	9.80	-5.66	-1.05	-5.02	4.8
2	-1	1.68	-0.297	-0.18	0.845	-0.163	-0.19	-1.44	-18.95	13.1
2	0	-63.4	-62.3	0.98	-16.4	-16.2	0.99	60.06	57.30	1.0
2	1	-1.03	3.01	-2.91	0.378	3.32E-2	0.09	18.71	-25.99	-1.4
2	2	2.29	37.0	16.17	2.28E-2	2.42	106.32	-0.39	-2.61	6.7
4	-4	-0.435	-5.12E-2	0.12	-1.59E-2	1.02E-2	-0.64	0.02	-1.55	-97.8
4	-3	0.742	0.267	0.36	-1.78E-2	5.51E-2	-3.09	-0.21	-1.67	8.0
4	-2	4.28E-2	2.66E-2	0.62	1.63E-3	-2.64E-2	-16.14	0.25	-0.83	-3.3
4	-1	-3.89E-2	-1.55E-2	0.40	-7.21E-3	7.25E-3	-1.01	0.23	3.53	15.1
4	0	-0.194	-0.206	1.06	1.07E-2	1.08E-2	1.01	-0.83	-0.50	0.6
4	1	5.20E-2	0.189	3.63	8.74E-4	8.15E-3	9.32	-3.06	4.89	-1.6
4	2	5.97E-2	0.543	9.09	-3.37E-3	-7.86E-3	2.33	0.12	-0.36	-3.0
4	3	0.360	1.38	3.85	3.19E-2	-1.31E-2	-0.41	-0.10	0.22	-2.1
4	4	5.51E-3	-0.402	-72.95	1.18E-2	-1.22E-2	-1.03	0.82	1.85	2.3
6	-6	6.44E-2	2.27E-2	0.35	6.41E-5	1.35E-4	2.10			
6	-5	-7.14E-2	-1.89E-2	0.26	1.28E-3	-1.25E-4	-0.10			
6	-4	9.69E-3	1.46E-3	0.15	2.66E-4	-2.83E-5	-0.11			
6	-3	3.51E-3	6.40E-3	1.82	-4.35E-4	6.30E-4	-1.45			
6	-2	5.41E-3	9.54E-4	0.18	3.04E-4	-4.55E-4	-1.50			
6	-1	-9.03E-3	5.79E-4	-0.06	-5.99E-5	-1.32E-4	2.20			
6	0	-3.01E-3	-3.45E-3	1.15	6.65E-5	7.74E-5	1.16			
6	1	-4.47E-3	-9.54E-3	2.13	-1.77E-4	-1.82E-4	1.03			
6	2	2.23E-2	2.50E-2	1.12	-1.96E-4	-1.26E-4	0.64			
6	3	2.66E-2	3.66E-2	1.38	4.84E-5	-1.38E-4	-2.85			
6	4	1.66E-2	3.99E-3	0.24	1.45E-4	4.20E-5	0.29			
6	5	3.15E-2	-7.93E-2	-2.52	-5.97E-4	1.71E-3	-2.86			
6	6	3.11E-2	6.12E-2	1.96	5.67E-4	3.57E-4	0.63			

9. References

1. Nicholas, H. M.; Vonci, M.; Goodwin, C. A. P.; Loo, S. W.; Murphy, S. R.; Cassim, D.; Winpenny, R. E. P.; McInnes, E. J. L.; Chilton, N. F.; Mills, D. P., Electronic structures of bent lanthanide(III) complexes with two N-donor ligands. *Chem. Sci.* **2019**, *10* (45), 10493-10502. DOI: 10.1039/C9SC03431E

2. Veryazov, V.; Malmqvist, P. Å.; Roos, B. O., How to select active space for multiconfigurational quantum chemistry? *Int. J. Quantum Chem.* **2011**, *111* (13), 3329-3338. DOI: 10.1002/qua.23068

3. Aquilante, F.; Pedersen, T. B.; Sanchez de Meras, A.; Koch, H., Fast noniterative orbital localization for large molecules. *J. Chem. Phys.* **2006**, *125* (17), 174101. DOI: 10.1063/1.2360264

4. Pipek, J.; Mezey, P. G., A fast intrinsic localization procedure applicable for ab initio and semiempirical linear combination of atomic orbital wave functions. *J. Chem. Phys.* **1989**, *90* (9), 4916-4926. DOI: 10.1063/1.456588
